# Extended Set of GoldenBraid Compatible Vectors for Fast Assembly of Multigenic Constructs and Their Use to Create Geminiviral Expression Vectors

**DOI:** 10.3389/fpls.2020.522059

**Published:** 2020-10-22

**Authors:** Jakub Dusek, Helena Plchova, Noemi Cerovska, Zuzana Poborilova, Oldrich Navratil, Katerina Kratochvilova, Cornelius Gunter, Raygaana Jacobs, Inga I. Hitzeroth, Edward P. Rybicki, Tomas Moravec

**Affiliations:** ^1^Laboratory of Virology, Institute of Experimental Botany of the Czech Academy of Sciences, Prague, Czechia; ^2^Department of Plant Protection, Faculty of Agrobiology, Food and Natural Resources, Czech University of Life Sciences, Prague, Czechia; ^3^Department of Experimental Plant Biology, Faculty of Science, Charles University, Prague, Czechia; ^4^Biopharming Research Unit, Department of Molecular and Cell Biology, University of Cape Town, Cape Town, South Africa

**Keywords:** *Agrobacterium tumefaciens*, cloning, *Nicotiana benthamiana*, plant virus vector, synthetic biology, transient expression

## Abstract

Methods for simple and fast assembly of exchangeable standard DNA parts using Type II S restriction enzymes are becoming more and more popular in plant synthetic and molecular biology. These methods enable routine construction of large and complex multigene DNA structures. Two available frameworks emphasize either high cloning capacity (Modular Cloning, MoClo) or simplicity (GoldenBraid, GB). Here we present a set of novel α-level plasmids compatible with the GB convention that extend the ability of GB to rapidly assemble more complex genetic constructs, while maintaining compatibility with all existing GB parts as well as most MoClo parts and GB modules. With the use of our new plasmids, standard GB parts can be assembled into complex assemblies containing 1, 5, 10 and up to theoretically 50 units in each successive level of infinite loop assembly. Assembled DNA constructs can be also combined with conventional binary GB-assemblies (1, 2, 4, 8… units). We demonstrate the usefulness of our framework on single tube assembly of replicating plant expression constructs based on the geminivirus Bean yellow dwarf virus (BeYDV).

## Introduction

Synthetic biology is an emerging field on the borderlines of several scientific disciplines, combining biology with engineering and computer science (Haseloff and Ajioka, 2009). One of the major challenges in synthetic plant biology is the combinatorial construction of complex multigene structures for functional testing in plant cells. Traditionally, genetic constructs were cloned using naturally occurring or engineered restriction endonuclease sites one DNA fragment at a time. However, traditional approaches have limited usefulness for the construction of multiple complex constructs in parallel. New hierarchical modular cloning techniques are used to build such multigene constructs: individual pre-assembled parts (modules) are assembled together using predetermined rules, and these units are in turn used in the assembly of incrementally more complex higher order structures. This principle offers several advantages, such as speed, versatility - i.e., the ability to share already prepared and tested parts between laboratories for new applications – as well as significant combinatorial capabilities and low cost (Ellis et al., 2011).

In 2008 a novel DNA assembly method – GoldenGate - was invented which exploits the ability of Type II S restriction enzymes to generate pseudo-random short overhangs that can be used to assemble several DNA fragments in the correct order (Engler et al., 2008). Before the DNA fragments can be used, they need to be “domesticated”: this is a process in which all the internal occurrences of the Type II S restriction sites are removed, and the ends are flanked with convergent recognition sites for the selected enzyme. Once the DNA part is verified by DNA sequencing it is immediately available for the incorporation into DNA constructs, and can be repeatedly reused with no additional modification, oligonucleotide synthesis, PCR, purification or sequence verification. Another great advantage of GoldenGate assembly is that parts of very different sizes can be easily assembled. Although GoldenGate started as a tool for assembly of plant transformation vectors, it has recently gained popularity also in synthetic biology of other organisms, including fungi (Hernanz-Koers et al., 2018) and yeasts (Lee et al., 2015; Obst et al., 2017).

The 4 nt overhangs generated by the restriction enzyme are ideal targets for the syntax standardization quickly adopted by the plant community. Two similar platforms using similar syntax have also emerged: these are MoClo (Weber et al., 2011) and GoldenBraid (Sarrion-Perdigones et al., 2011). Most of the parts designed for MoClo can be used without modification in the GB system and vice versa. In fact, in this work we have used multiple pre-made DNA parts from both systems obtained from Addgene. While basic parts are mostly mutually exchangeable between MoClo and GB standards, the philosophy of assembling these basic elements into higher orders structures is different. MoClo uses a complex vector toolkit with multiple domestication vectors, levels 1 and 2 or M and P vectors, and suitable endlinkers. The standard level 2 assembly in MoClo consists of up to 7 transcriptional units (TUs), which is sufficient for most applications, however, no additional parts can be added afterward. If more complex assemblies are desired, a specific linker module with an additional color marker gene and restriction sites needs to be present in the original assembly. This linker then allows cloning of subsequent levels of construct. GB by contrast uses a simple pairwise/binary approach, where more complex structures are assembled through consecutive turns of infinite loop switching between α and Ω level plasmids. For basic functionality only five vectors are needed. An additional four vectors enable cloning of all TUs in both forward and reverse orientations. Design of GB cloning strategies is also greatly simplified by the website www.gbcloning.org, that facilitates automatic design of primers and other functionalities.

In this work, we describe an expanded set of GB-compatible α-level plasmids that allow single reaction assembly of more complex genetic structures, while keeping compatibility with the large collection of finished and tested GB modules. The new plasmids are based on the backbone of the plant expression vector plasmid pGreen (Hellens et al., 2000), which is derived from pDGB1α1. Unlike the original GB standard that uses infinite loop of binary assemblies, the new plasmids enable assembly of up to five TUs in single tube reaction. Users can alternatively use both strategies, depending on their needs. In our design, we have replaced the α1 plasmid with a set of four new plasmids named α11, α12, α13, and α14. An additional fifth plasmid named α13R allows insertion of cassette TUs in the opposite orientation. Plasmids α2, Ω1 and Ω2 as well as universal domestication plasmid pUPD2 were not changed and can be used to combine existing binary-assembled modules with the new 5-piece system.

To demonstrate the feasibility of the novel approach we chose to use the novel five piece assembly standard to create multicomponent replicating plant viral expression vectors based on Bean yellow dwarf virus (BeYDV), and non-replicating vectors based on the hypertranslatable Cowpea mosaic virus (CPMV) 5′/3′ untranslated regions (UTRs) derived from pEAQ vectors.

Bean yellow dwarf virus is a mastrevirus from the *Geminiviridae* family. Its relatively small genome consists of a single-stranded circular DNA of approximately 2.6 kb that can multiply to very high copy numbers in plants (Timmermans et al., 1992; Baltes et al., 2014). The BeYDV genome contains a long intergenic region (LIR), two sense strand open reading frames (ORFs) V1 and V2, a short intergenic region (SIR) and an additional 2 ORFs C1 and C2 in the complementary sense. The virion sense ORFs encode the movement protein and coat protein while the complementary strand ORFs encode two replication associated proteins - Rep and RepA (Liu et al., 1997; Huang et al., 2009) that are produced from a single intron-containing transcript. Replicating BeYDV clones are popular vectors for the overexpression of pharmaceutical proteins (Hefferon and Fan, 2004; Huang and Mason, 2004; Regnard et al., 2010); the one used in this work is derived from a mild strain of the virus (Halley-Stott et al., 2007). Their major advantage is that the replication can substantially increase the transgene DNA and mRNA copy number per cell. BeYDV has also been used in genome editing experiments as a donor of DNA for homologous recombination (Baltes et al., 2014).

The pEAQ (Easy And Quick) vectors are based on hypertranslational properties of CPMV RNA-2 5′ and 3′ UTRs (Sainsbury and Lomonossoff, 2008; Sainsbury et al., 2009). Unlike other viral vectors, pEAQ vectors do not replicate within the host cells but rely on mRNA that is very stable and efficiently binds ribosomes, resulting in massive protein overexpression in the infiltrated area. Both BeYDV-derived and pEAQ vectors are not subject to single-cell/single vector exclusivity seen with plant RNA virus-based vectors. This is an important advantage for many biotechnological applications, where it is necessary to co-express two or more proteins in the same plant cell (Thuenemann et al., 2013). In this study, we have hypothesized that the combination of high DNA copy number obtained by BeYDV replication with the hypertranslational properties of CPMV derived mRNA could lead to even higher expression levels than what is normally achieved with either system alone. The extended GB syntax allowed us to assemble and test multiple relatively large and complex constructs with only a limited number of basic parts.

## Materials and Methods

### Preparation of Novel GB3.0 Compatible α-Level Plasmids

Five novel α-level plasmids named α11, α12, α13, α14, and α13R were based on original GB pDGB1α1 plasmid – a derivative of the pGreen vector (Hellens et al., 2000). The lacZ gene was amplified using specific primers listed in Supplementary Table S1 and Phusion DNA polymerase (Thermo Fisher Scientific, United States). The PCR products were excised from 0.8% agarose gel, purified using gel extraction kit (Qiagen, Germany) and subjected to the second round of PCR using extension primers dB1a-extF and dB1a-extR (Supplementary Table S1) that created 35 nt long overhangs homologous to *Eco*RI digested α1 plasmids on both ends. As a source of vector backbone, we chose to use plasmid with the inserted sequence, since it enabled the use of blue/white screening to identify recombinants. One μg of pDGB1α1 plasmid containing 1 kb long RB7 matrix attachment region (MAR) insert was digested with *Eco*RI HF (New England Biolabs, United States) and 2.6 kb long plasmid backbone was gel purified (Qiagen, Germany). 100 ng of purified PCR product and 50 ng of linearized backbone plasmid were mixed with 1 μl of SLiCE reagent (Zhang et al., 2012) and incubated for 60 min at 37°C. The SLiCE reaction mixture was then transferred to chemically competent *E. coli* Top10 (Thermo Fisher Scientific, United States) cells and plated on solid LB media with 50 μg/ml kanamycin and X-gal (Thermo Fisher Scientific, United States). Two blue colonies were selected from each plate, plasmid DNA purified using the GeneJET Plasmid Miniprep Kit (Thermo Fisher Scientific, United States) and verified by Sanger sequencing (GATC Biotech, Germany).

### DNA Parts

Multigenic DNA constructs were created using existing DNA parts from the GB 2.0 kit obtained from Diego Orzaez (Addgene kit # 1000000076, namely basic UPD, α and Ω plasmids, P19 CDS, NOS terminator), the MoClo Toolkit (obtained from Sylvestre Marillonnet & Nicola Patron, Addgene kit # 1000000044, namely 35S promoter, GUS, GFP and DsRed reporter genes) along with novel parts reported in this work. These novel parts were domesticated either at the Laboratory of Virology IEB, Prague (MAR sequences, short stuffers, novel α 11–14 entry plasmids, pEAQ derived CPMV 5′/3′ UTRs, CCAT-AATG intron) or at the Biopharming Research Unit, University of Cape Town (all BeYDV derived sequences – LIR, SIR, and Rep/RepA). Primers for the domestication of novel standard GB3.0 parts used in this work were designed using GB3.0 automatic primer designer^1^. All primers are listed in Supplementary Table S1. For cloning purposes, we have used Phusion DNA polymerase (Thermo Fisher Scientific, United States). PCR products were column purified either from gel or directly from the 50 μl reactions using High Pure PCR Product Purification Kit (Roche, Switzerland).

For restriction/ligation assembly we used the standard protocol described in Sarrion-Perdigones et al. (2013) with either 45 cycles or 20 cycles. Both *Bsa*I and *Bsm*BI restriction enzymes and T4 ligase were obtained from Thermo Fisher. It is important to note that proper warming and resuspension of the 10× T4 ligase buffer (5 min at 40°C followed by 1 min vortex) prior to making aliquots is necessary. The DTT tends to form an insoluble precipitate in the freshly thawed buffer which leads to insufficient *Bsm*BI cleavage due to low DTT levels.

**FIGURE 1:**
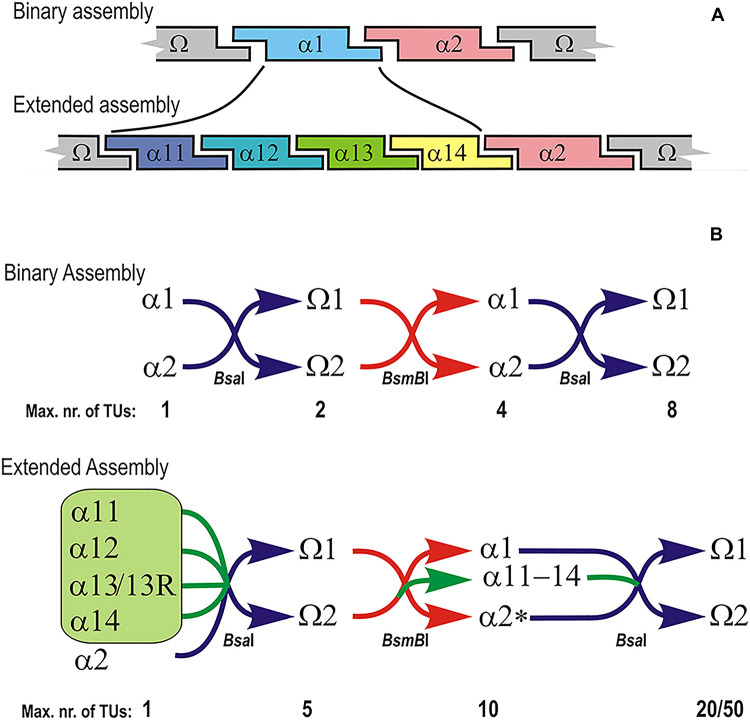
Schematic representation of the design of extended GoldenBraid assembly framework. **(A)** In the extended assembly the original α1 plasmid is replaced with 4 novel α-level plasmids in such a way that the 5′ end of α11 and 3′ end of α14 are identical to the ends of original α1. α2 and both Ω 1 and 2 plasmids are retained from GoldenBraid 2.0 system. **(B)** Examples of infinite loops in classical binary GoldenBraid assembly vs. extended assembly with the maximum number of assembled transcription units (Sarrion-Perdigones et al., 2013).

Sequences of novel parts were verified using Sanger sequencing (GATC Biotech, Germany). The sequences of multi-cassette expression vectors are included in the Supplementary File S1. For sequence verification of larger multigene constructs by Sanger sequencing, we also provide sequencing primers directed outside of MAR domains (Supplementary Table S1). Schematic diagrams of the T-DNA regions of the plasmids pGB-R-DsRed-GFP, pGB-R-GFP-DsRed, pGB-G-GFP-DsRed, pGB-R-DsRed, pGB-R-GFP, pGB-E-DsRed-GFP, pGB-E-GFP-DsRed, pGB-E-MAR-GFP, pGB-E-MAR-GFPR, pGB-E-MAR-GFPi, pGB-E-GFP are shown in Figure 1. The resulting plasmids were transformed into *E. coli* Top10 (Thermo Fisher Scientific, United States), and after isolation and sequence verification they were eventually transformed into *Agrobacterium tumefaciens* EHA105 by the freeze-thaw method (An, 1987).

### Agroinfiltration Procedure

For transient expression of multigene constructs, we used *Agrobacterium tumefaciens* strain EHA105. Agrobacteria were grown in 4 ml of LB medium with appropriate antibiotics (100 μg/ml spectinomycin, 50 μg/ml rifampicin) under agitation (250 rpm) at 26°C overnight. The bacteria were pelleted by centrifugation for 10 min at 3700 × *g* and then resuspended in the infiltration buffer - 10 mM MES pH 5.6, 10 mM MgCl_2_ and 0.1 mM acetosyringone. The optical density (OD_600_) was measured directly in clear polystyrene culture tubes (Gama, Czechia) using a densitometer (DEN-1; Biosan, Latvia). We used bacteria at the concentration of OD = 1 if not indicated otherwise. The resulting bacterial suspensions were injected into fully expanded leaves using a syringe without a needle, either alone or as a mixture of several strains, through a small puncture (Huang and Mason, 2004).

### Plant Growth

For infiltration, we used 5- to 6-week-old *Nicotiana benthamiana* plants grown in growth rooms. The temperature in the growth rooms was set to 24°C throughout the experiment. All plants were grown in a 16/8 h light/dark regimen. Complete fertilizer (Kristalon, Czechia) was applied weekly. Plants were grown under 1:1 mixture of fluorescent tubes 36W/840 G13 MASTER TL-D (Philips, Netherlands) and plant growth specific LED tubes 8BEN-120cm-15W-GL-230V-T-G13 (Frontier Technologies, Czechia) (Janda et al., 2015). The light intensity at the base ranged from 90 to 140 μmol.s (Janda et al., 2015).

### Fluorescence Measurement and Imaging

For each experiment (biological replicate) we inoculated 3–5 leaves of 3–5 plants. Three representative leaves were selected on the basis of being mechanically undamaged, with sufficiently large infiltrated areas and showing visible fluorescence when observed under UV light (approximately 400 nm) from a handheld lamp. In total, we prepared three extracts for each construct. Each extract consisted of three disks, originating from the same leaf. First we determined relative fluorescence readings for each construct on each leaf by comparison with the positive control (pGB-R-GFP, respectively, pGB-E-MAR-GFP for GFP, and pGB-R-DsRed for DsRed). Then we calculated the average fluorescence from multiple leaves. Samples were collected 5 days post inoculation (DPI) and were frozen in liquid nitrogen. The tissue was homogenized in tubes with 1 g of 1.3 mm zirconium milling silica beads in 300 μl PBS with 0.05% Tween-20 and 0.03% sodium azide using a FastPrep-24 (MP Biomedicals, United States). Samples were then normalized to total protein concentration of 1 mg/mL using Pierce^TM^ BCA Protein Assay Kit (Thermo Fisher Scientific, United States). The fluorescence of leaf extracts containing the expressed GFP and DsRed were measured on a Tecan-F200 instrument (Tecan, Austria) using a GFP filter set (485/20 and 535/25 nm excitation/emission, respectively) and a DsRed filter set (535/25 and 586/20 nm). The identity of respective fluorescent proteins was confirmed by western blotting using rabbit polyclonal anti-GFP antibody (EXBIO, Czechia) at 1:1,000 dilution, and rabbit polyclonal anti-RFP antibody (MBL International, United States) at 1:1,000 dilution. The band intensities were quantified with the ImageJ software (Version 1.48). GFP (Abcam, United Kingdom), and DsRed (Fraunhofer IME, Germany) proteins in the range of 0.005–1.0 mg/mL were used to create the standard curves.

Whole plants were imaged using an Olympus E-PL3 digital camera with a Zeiss Jena Pancolar (50/1.8) lens. GFP was visualized using self-assembled UV LED fixtures (approximately 400 nm) and LEE 124 Dark Green filter. Similarly DsRed fluorescence was visualized using green LEDs (approximately 530 nm) and LEE 019 Fire filter (both filters Thomann, Germany). For fluorescent microscopy we used a Leica CTR 5000 instrument with GFP and N2.1 filter cubes (Leica Microsystems, Germany).

### Quantitative PCR

The copy number of geminiviral replicons was estimated by qPCR on a LightCycler^®^ 480 instrument (Roche, Switzerland). We compared the accumulation of vector DNA in infiltrated plant tissue. Two similar constructs were tested: these were pGB-R-GFP-DsRed (which includes functional Rep/RepA) and pGB-G-GFP-DsRed (Rep has been replaced with gene coding for GUS). The scheme of the constructs is shown in Figure 4. For the quantitation, we used primers designed for DsRed (Supplementary Table S1), with normalization performed using Nb actin primers. The *N. benthamiana* leaves were infiltrated with replicating (pGB-R-GFP-DsRed) and non-replicating (pGB-G-GFP-DsRed) constructs. DNA was extracted from infiltrated leaves 5 DPI using salt and SDS extraction (Dellaporta et al., 1983) and used as a template for qPCR. Two samples for each constructs were measured. Each sample contained 3 × 2 leaf disks from upper, middle and lower leaves. Calibration curves based on spiked known concentrations of purified plasmid DNA were constructed.

## Results

### Extended Set of GB3.0 Compatible α-Level Plasmids

We replaced the existing α1 plasmid of the Golden Braid 3.0 system with four new plasmids named α 11 to 14 (Figure 1). The 4 nt overhang generated during the *Bsm*BI reaction (Table 1) on the 5′ end of α1 is identical with the 5′ overhang of the α11 fragment. Similarly, the 3′ end overhang of α1 is identical with that of α14. The engineered ends of plasmids 11 through 14 are shown in Table 1. With the exception of these unique 4 bp overhangs, the vectors are otherwise identical to the parental pDGB1α1 plasmid. Plasmids α2 and all Ω plasmids were retained for compatibility and can be used to shuffle existing modules between assembly systems.

**TABLE 1:** Overhangs generated using *Bsm*BI.

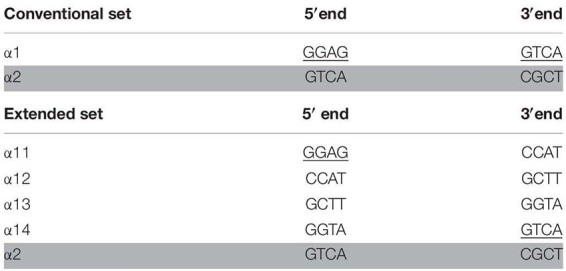

*The 4 nt overhangs generated upon *Bsm*BI digestion of various α-level plasmids. α2 is identical in both sets. Underlined tags should symbolize that the ends α1 plasmid were split into 4 new plasmids.*

The novel α plasmids were generated using PCR and homology based *in vitro* recombination (SLiCE) as described in the Methods section. Plasmid DNA from blue colonies (two colonies per construct) were isolated and verified by sequencing. One of the unique features of the GB plasmid set is that inserts can be easily released by restriction with one common and inexpensive restriction enzyme (RE). This RE is unique for each plasmid, so that in case of uncertainty, both the backbone and the insert can be easily confirmed by restriction digest. In all plasmids of this new set we decided to use the same flanking enzyme recognition site: this was *Eco*RI. The benefit of simpler management of restriction mapping verification of multiple constructs processed in parallel outweighs the impossibility of identifying plasmid backbones by RE digestions.

To increase the design flexibility of the extended set of plasmids we have also prepared a new α13 plasmid with an inverted insertion cassette named α13R. In future, other plasmids in the set (11, 12, and 14) can also be created in inverted orientation, however, at this point we believe that the flexibility offered by the current option of having 2 (α13R and α2R) out of 5 TU in reverse orientation is sufficient.

Since the number of parts in the assembly is fixed to five, there is also an increased need to use spacers or stuffer fragments in projects where more than two but fewer than five TUs are used. For this purpose, we have generated several spacers. As a first option, we have generated two short random DNA fragments of 35 and 55 bp in length that were cloned into pUPD2 as GGAG-CGCT fragments. Since most of our projects are aimed at overexpression of proteins, we have also domesticated four different matrix attachment regions (MARs) that can be used both as spacers and expression enhancers at the same time.

We set up in total 144 5-fragment reaction mixes, of which 115 resulted in plasmids with the expected restriction digestion patterns. Normally we use just two colonies to isolate plasmid DNA; in rare cases when neither corresponds to the expected map, two additional colonies are used for plasmid isolation. If neither of four colonies is correct the whole plate is discarded, and a new reaction is set up. Such failed attempts were recorded 29 times in total. In most of these cases, the reasons that lead to failed reaction could be identified afterward and can be seen in Table 2. Overall, the success rate of the five fragment assembly in our experience is similar to the original binary assembly.

**TABLE 2:** Percentage of correctly assembled clones using either binary or 5-part assembly process.

**Assembly method**	**Total**	**Correct clones identified**	**Failure**
Binary	35	24 (68.6%)	4 (9.5%) B; 7 (16.7%) C; 11 (31.4%) total
Quintuple	144	115 (79.9%)	12 (7.4%) A; 11 (6.7%) B; 6 (3.7%) C; 29 (20.1%) total

*A, competent cells failure, only colonies with no plasmid DNA; B, insufficient DTT levels in the reaction mixture, wrong and random restriction patterns; C, other, unknown.*

### Adaptation of BeYDV-Derived Expression Vector to GB Standard

**FIGURE 2:**
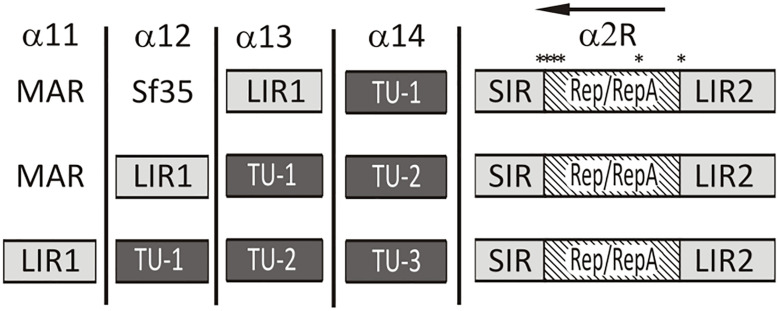
Schematic representation of possible assemblies of GB vectors with BeYDV elements. The mutations inserted into complementary part of BeYDV genome are symbolized by an asterisk. The design allows the insertion of up-to 3 transcriptional units (TU) in one replicon in a single assembly reaction. Stuffer fragment (Sf35) is a short 35bp linker.

To demonstrate the usefulness and greater flexibility of our extended set of vectors we decided to adapt existing a geminiviral expression vector into the GB standard. Only few relatively short sequences are necessary to initiate circularization and rolling circle replication of geminiviral replicons in host cells. These essential parts are long and short intergenic regions (LIR and SIR, respectively), and the Rep/RepA ORF. Typically the T-DNA with the replicon contains tandem copies of LIR that provide the limits of the excised circularized replicon DNA. The SIR sequence is placed in between the LIR copies. Insertion of a Rep/RepA module in the replicon is optional; it can be provided *in cis* on the same plasmid or *in trans* on separate plasmids. If it is present on the same backbone it is typically placed in the antisense orientation between the SIR element and the second copy of the LIR (Hefferon et al., 2004; Regnard et al., 2010). Our domestication strategy (Figure 2) was to create two different versions of the LIR sequence. LIR1, that is used as the 5′ limit of the replicon, has the syntax of the entire TU (GGAG-CGCT, A1-C1 according to GB convention). LIR2 which is used for the 3′ limit of the replicon has the syntax of a standard promoter (GGAG-AATG, A1-B2). The Rep/RepA CDS was cloned as a standard protein coding region (AATG-GCTT, B3-B5). Finally, the SIR region was cloned as the standard terminator (GCTT-CGCT, B6-C1). Only one *Bsa*I site (344 bp from the Rep ATG) had to be mutated in the Rep/RepA gene: fortunately, it is located in a sequence encoding just one reading frame and thus it was possible to introduce a silent mutation.

After domestication and sequence verification the LIR1 was cloned into vectors α 11 to 13. For the c-sense part of the genome (LIR2-Rep/RepA-SIR) we took advantage of pDGB1α2R vector that allows the assembly of TU in antisense orientation. The resulting clone contained a 1 nt mutation before the ATG start to accommodate the GB syntax, a 1 nt silent mutation in the *Bsa*I site, and a 5 nt insertion after the RepA stop codon to accommodate the GB syntax. As a non-replicating control, we made a similar cassette containing the GUS gene (LIR2-GUS-SIR). This flexible design allows the construction of replicating geminivirus-derived vectors carrying up to three standard transcription units (Figure 2). In this work, we have used vectors carrying either one or two transcription units. The assembled vectors were verified using restriction mapping and were then used for infiltration experiments.

### qPCR Quantification of Vector DNA

**FIGURE 3:**
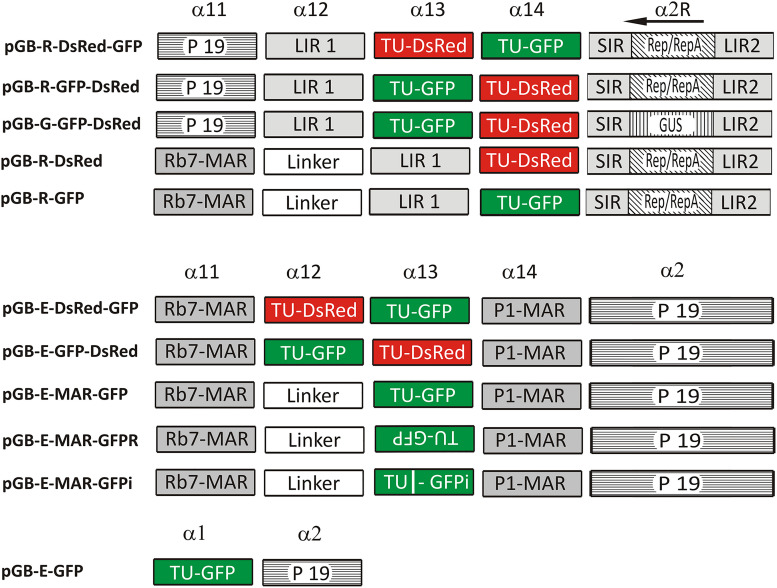
Schematic representation of constructed assemblies. pGB, GoldenBraid plasmids; R, replicating constructs; G, contains GUS gene instead of Rep; E, non-replicating vectors derived from pEAQ; GFP/DsRed, fluorescent reporter genes under 35S promoter, Nos terminator with CPMV derived 5′/3′ UTRs; GFPR, identical GFP fluorescent reporter cassette in reverse orientation; GFPi, GFP reporter cassette in which short intron was inserted between CPMV 5′ NTR and GFP start codon; MAR Rb7/P1, matrix attachment regions; Linker, stuffer fragment is a 35 bp long linker; P 19, silencing suppressor derived from Tomato bushy stunt virus. LIR 1, 2, long intergenic region derived from BeYDV; SIR, short intergenic region derived from BeYDV.

**FIGURE 4:**
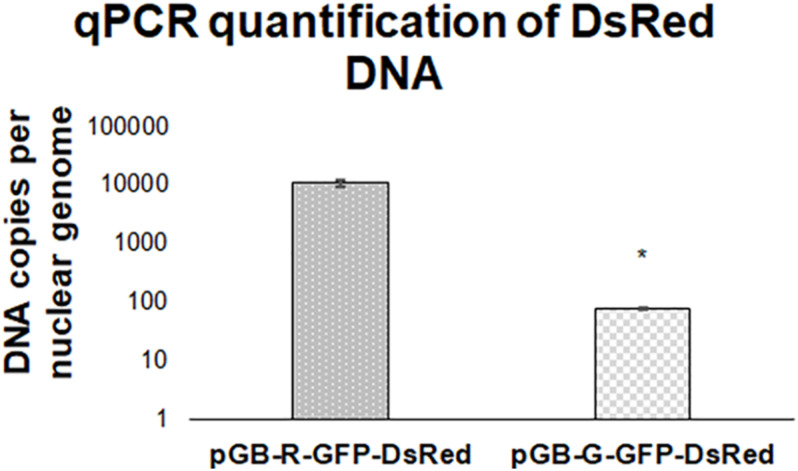
qPCR estimation of the number of vector DNA copies per plant nuclear genome. The plant extracts inoculated with replicating pGB-R-GFP-DsRed construct contain approximately 140 times more copies of DsRed DNA than the extracts inoculated with analogous non-replicating pGB-G-GFP-DsRed construct. Samples were collected at 5 DPI. The graph shows values obtained from one representative biological replicate. Statistically significant differences at *p* = 0.05 are marked by an asterisk.

To verify the ability of the novel GB derived vectors with BeYDV elements to replicate the DNA within the plant cell nucleus we attempted to quantify the vector DNA using qPCR. The plant tissue was infiltrated with two similar constructs: these were pGB-R-GFP-DsRed (which includes Rep) and pGB-G-GFP-DsRed (contains GUS gene instead of Rep, see Figure 3). Based on the qPCR results we estimate that replication-capable constructs accumulated 150–170-fold more copies per nuclear genome than a similar construct without the functional Rep (Figure 4).

### Transient Expression of Reporter Genes Using Replicating and Non-replicating Vectors

Simultaneous expression of two or more genes is important for the production of multimeric proteins such as antibodies. The pEAQ vectors based on hypertranslatable 5′/3′ UTRs from CPMV have been successfully used to express antibodies in plants (Sainsbury and Lomonossoff, 2008; Sainsbury et al., 2009). Replicating geminiviral vectors were also successfully used for the same purpose (Hefferon and Fan, 2004; Huang and Mason, 2004; Regnard et al., 2010). Here we hypothesized that combination of both high transgene copy number provided by the replicating vector with highly efficient 5′/3′ UTRs derived from CPMV could lead to even higher expression levels.

**FIGURE 5:**
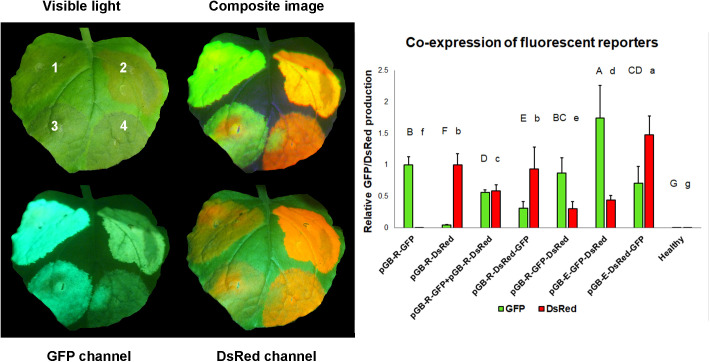
Co-expression of two fluorescent reporter genes. *N. benthamiana* leaves were infiltrated with replicating and non-replicating constructs expressing GFP and DsRed that were either present on one plasmid or on two co-infiltrated plasmids. Relative expression of fluorescent reporters 5 days after infiltration. **Left panel** Representative image of leaf infiltrated by EHA105 carrying: (1) pGB-E-GFP-DsRed; (2) pGB-E-DsRed-GFP; (3) pGB-R-GFP-DsRed; (4) pGB-R-DsRed-GFP and visualized under visible light, GFP channel or DsRed channel. **Right panel** Graphical representation of GFP and DsRed co-expression. The fluorescence obtained with single reporter replicating vectors were used as 1.0. Statistically significant differences at *p* = 0.05 are marked by different letters.

The levels of fluorescent protein expression from the non-replicating constructs were higher than from their replicating counterparts (Figure 5), despite the fact that exactly the same cassettes were used for the expression of the fluorescent marker in both variants. While the replicating vectors lead to early development of leaf necrosis, the non-replicating variants did not show a tendency to cause necrosis even after longer periods (Supplementary Figure S2). To test the longevity of transient protein expression we replaced the GFP gene in pGB-E-MAR-GFP with a mutant version of DsRed called Timer (Takara Bio Inc., Japan). This mutant fluorescent protein produces a green fluorescent signal for several hours after protein synthesis, which then slowly matures to red fluorescence. Even 2 weeks after infiltration we observed cells showing green fluorescence, suggesting that protein expression from hypertranslatable mRNA with CPMV 5′/3′ UTRs did not stop even after such long periods (see Supplementary Figure S1).

Another phenomenon we observed was a stronger expression of the first transcription unit (about 2–3-fold) in both replicating and non-replicating constructs. This is an important finding for future strategy design to express multi-subunit proteins where their proper function depends on correct ratios of individual subunits, such as antibodies.

**FIGURE 6:**
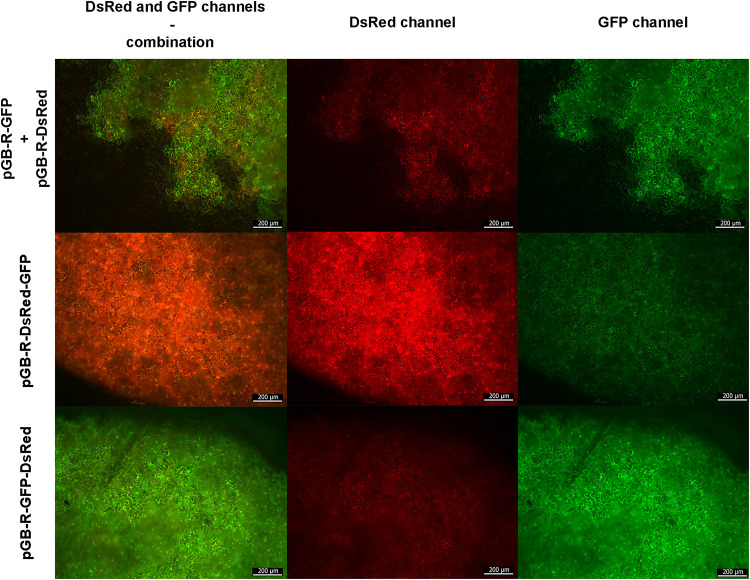
Composite microscope image showing the fluorescence of the replicating constructs 5 DPI. The composite images from both red and green channels are in the left column, DsRed channel in the middle and GFP channel on the right. **Top row** shows the signal from two independent agrobacteria and plasmids, **middle** and **bottom row** are replicating constructs with two TUs (pGB-R-DsRed-GFP and pGB-R-GFP-DsRed, respectively). The scale bar shows 200 μm.

Based on the results summarized in the graph (Figure 5) we hypothesized that in order to achieve the highest co-expression of two proteins, the best strategy would be to use two independent non-replicating plasmids in a mixture of two *Agrobacterium* strains. However, it was impossible to estimate the co-expression at the cellular level using a plate reader, so the infiltrated area was examined under a fluorescent microscope. This analysis indicated cells showing higher GFP or DsRed signal in tissue infiltrated by two independent plasmids (Figure 6).

### Strategies to Mitigate the Necrosing Phenotype

It has been shown previously that the necrosis elicited by BeYDV replication in *N. benthamiana* leaves can be reduced by the use of different strain of *Agrobacterium* (Diamos et al., 2016), and/or insertion of a MAR element downstream from the expression cassette. However, we did not observe a difference between GV3103 and EHA105 strains and saw only a relatively mild effect when RB-7 MAR was used. Other MAR elements (TM2, TM6 derived from tobacco and P1 derived from soybean) showed no effect. Next, we tested the hypothesis that lower density of *A. tumefaciens* during infiltration leads to faster reduction of expression from non-replicating constructs compared to replicating constructs. We tested 2-fold *Agrobacterium* suspension dilution series starting from OD600 = 0.25 to OD600 = 0.015. We also hoped that the dilution of *A. tumefaciens* might reduce or delay the necrosis. Leaves infiltrated with the non-replicating vector showed no signs of necrosis even at the highest bacterial concentration (OD600 = 0.25), and gradual decrease of fluorescence intensity corresponding with decreasing bacterial density. On the other hand, all dilutions of replicating constructs except the most dilute showed necrosis by the fourth DPI (Supplementary Figure S2). Thus, the non-replicating constructs are easier to use, since they are not that sensitive to *Agrobacterium* concentration, and can tolerate later harvest after infiltration.

### Matrix Attachment Region

One of the features of our novel extended set of GB vectors is that successful assembly requires exactly five DNA fragments to be combined. For assembly of just 2 TU modules, preferably the conventional binary GB assembly could be used. For assemblies of 3 or 4 TU parts, however, it is preferable to use vectors from our novel set along with appropriate spacers. For this purpose we have domesticated four matrix attachment regions: these are RB-7 (Allen et al., 1996; Halweg et al., 2005; De Bolle et al., 2007), TM2 (Xue et al., 2005), TM6 (Ji et al., 2013) from tobacco and P1 from soybean (Breyne et al., 1992; Petersen et al., 2002). We have also cloned a 700 bp intron from the pKANNIBAL vector (Wesley et al., 2001) and two shorter random sequences of 35 and 55 bp (for the complete sequences see Supplementary File S1).

**FIGURE 7:**
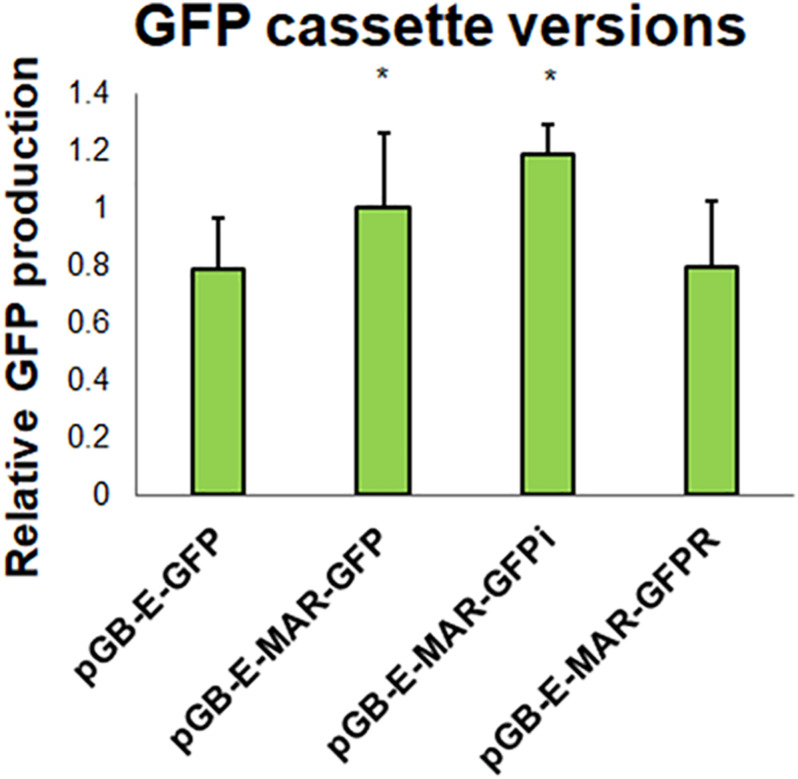
One half of a leaf was infected by pGB-E-MAR-GFP, second half was infected by pGB-E-GFP/pGB-E-MAR-GFPi/pGB-E-MAR-GFPR (*A. tumefaciens* strain EHA105, OD_600_ = 1). These are relative values, so they were normalized to single cassette construct pGB-E-MAR-GFP. The fluorescence of leaf disks containing the expressed GFP was measured on a Tecan-F200 instrument (Tecan, Austria) 5DPI. Graph shows values obtained from one representative biological replicate. Statistically significant differences at *p* = 0.05 are marked by an asterisk.

It has been shown previously that MAR sequences can improve transgene expression and reduce position bias (Halweg et al., 2005), and can also be helpful in transient expression (Diamos et al., 2016). Here we wanted to test the effect of MAR sequences on the expression of non-replicating constructs with a single TU – GFP. As can be seen from Figure 7 the MAR sequences improved the expression of the reporter gene by about 25%. Additional improvement was obtained with pGB-E-MAR-GFPi where the CPMV 5′ UTR contained a short intron (*A. thaliana* ribose 5S EF intron). The relative orientation of the reporter cassette with respect to vector backbone does not seem to play a role.

## Discussion

Here we report an extended set of novel α-level plasmids for the GB vector construction framework. We attempted to combine the advantages of the GB (the infinite loop design without the need of endlinkers) and MoClo systems (its ability to assemble more complex multigene systems in a single reaction). At the same time, we wanted to preserve the compatibility with existing GB binary assemblies and modules. The design-build-test feedback loop requires advanced tools to assemble complex multigenic constructs consisting of multiple combinations of standard parts. DNA assembly of complex or combinational constructs is frequently a rate-limiting step in the testing cycle of synthetic biology. Costs associated with the design of genetic circuits mainly stem from the synthesis of novel DNA fragments and sequence verification of PCR amplified assemblies. DNA synthesis and sequencing also constitute to major time delays in testing novel improved constructs. After the DNA fragment is domesticated for the MoClo and GB assembly methods, it does not require additional sequencing or oligonucleotide synthesis, making the design-build-test loop less expensive and faster.

In this work, we wanted to simplify the process for combinational assembly of more complex genetic circuits and maintain compatibility with the current GB 3.0 system. The recursive use of two restriction enzymes and selection markers in an infinite loop without the need of endlinkers is a great design feature of GB3.0 as it simplifies the reuse of intermediate constructs for new purposes. However, the construction of more complex or combinatorial genetic circuits based on binary assembly as in GB is slow and requires multiple steps consisting of cloning, transformation, isolation, and verification of large number of intermediate plasmids. With increasing number of variable TUs the number of cloning steps and intermediate plasmids becomes more and more limiting, wasting reagents, time and resources. While the MoClo system with its ability to assemble multiple TUs in one reaction is faster, it makes the reuse of multigene MoClo modules for different purposes more complicated. Recently at least two new frameworks combining the strengths of both GB and MoClo platforms have been described: these are Loop assembly (Pollak et al., 2019) and Möbius (Andreou and Nakayama, 2018). Both these methods achieve the assembly of complex structures by infinite switching between two levels of plasmids similar to GB, but instead of binary they use quadruple assembly in each level. Both systems introduce new Type II S enzymes –*Sap*I for Loop assembly and *Aar*I for Möbius. While both these enzymes are rare cutters recognizing 7 bp sequences which should be relatively scarce in existing domesticated MoClo or GB part libraries, their absence cannot be guaranteed. Enzyme *Aar*I is also rather expensive – currently ∼ 45 times more expensive than *Bsa*I (Catalog Thermo Fisher Scientific, United States, December 2019), which might be prohibitive for some users. Our approach is different in that it not only preserves compatibility with all existing GB and most MoClo parts (provided they lack internal *Bsm*BI site) that can be used directly without any modifications, but it provides considerable compatibility also with existing higher level GB modules. From the 4 plasmids (2 α and 2 Ω) in the original GB system, three plasmids can be used without any changes in our new system. Only existing modules that were cloned in α1 plasmids are not directly compatible with the extended version; to achieve compatibility, one additional cloning step for conversion into Ω-level might be required. One of the advantages of our design is that both binary and quintuple assemblies can be combined as needed.

As the extended vector set requires exactly 5 α-level plasmids to be assembled at once, we have anticipated its use will increase the need for linkers or spacers. In this work we have used two types of linkers: these are short random sequences of 30–50 bp, and longer 500–1100 bp spacers containing known MAR sequences. MAR sequences were shown to reduce the event-to-event heterogeneity observed in permanent transformants (Allen et al., 1993), and may significantly improve the expression levels in transient settings (Allen et al., 1996). In our experiments, we have observed a statistically significant positive effect of RB7 and P1 MAR on the expression of GFP in non-replicating vectors (Figure 7).

During more than 3 years of experience with the extended vector set, we have observed a similar rate of cloning success both with binary and quintuple assemblies.

In summary, the set of novel GB backbone plasmids for quintuple assembly bring the following advantages: (a) faster assemblies of vectors with more than 2 TUs; (b) the need of lower number of intermediate plasmids; (c) allows variability in the central part of the assembly, not only on either end; (d) preserves compatibility with all existing GB parts and most GB modules (α2, Ω1 and Ω2).

In this work, we have shown the advantages of our framework to assemble simple replicating vectors derived from BeYDV or non-replicating vectors expressing two fluorescent markers. We have shown that the topology of both replicating and non-replicating multigene vectors plays a role in the expression strength in transient settings. This is in agreement with previous studies performed with the pEAQ system (Sainsbury et al., 2009), where the authors observed a marked increase in total antibody production when the gene for the heavy chain was placed upstream from the gene for light chain, versus the opposite topology. Based on our results the TU positioned nearest to the left border is expressed more strongly than TU located downstream (Figure 5). Recently we have also obtained similar results from plant cell packs derived from tobacco BY-2 cells (Poborilova et al., 2020). Co-ordinated co-expression of two or more transcripts from a single vector is important for the expression of multisubunit proteins such as virus like particles, antibodies or vaccines. The data obtained in this study were used to construct optimized vectors for the expression of several mouse and human monoclonal antibodies (manuscript in preparation).

The extended GB plasmid set described in this work as well as the MAR elements and short linkers are available through Addgene (plasmid numbers #106207–#106216).

## Data Availability Statement

The plasmids generated for this study can be found in the https://www.addgene.org/search/catalog/plasmids/?q=moravec.

## Author Contributions

TM and ER conceived the ideas. HP and TM designed the primers for domestication and sequencing. JD, ZP, KK, CG, RJ, and IH domesticated and constructed the vectors. ON and NC transformed and cultivated *Agrobacterium tumefaciens* for the experiments. TM and JD made the measurement, did microscopy, and evaluation of experiments. All authors contributed critically to the draft and the writing of the manuscript and gave final approval for publication.

## Conflict of Interest

The authors declare that the research was conducted in the absence of any commercial or financial relationships that could be construed as a potential conflict of interest.

## Footnotes

1 www.gbcloning.org
