# Supplementary material for: Extended Set of GoldenBraid Compatible Vectors for Fast Assembly of Multigenic Constructs and Their Use to Create Geminiviral Expression Vectors
Source: Front Plant Sci. 2020 Oct 22;11:522059. doi: 10.3389/fpls.2020.522059 (PMC7641900; doi:10.3389/fpls.2020.522059)
Supplement: Supplementary file 1 [file Data_Sheet_1.docx]

Supplementary Material

# Supplementary Figures and Tables

## Supplementary Tables

**Supplementary Table 1**

| **Primer** | **Sequence** | **Length** |  |
| --- | --- | --- | --- |
|  | ***Cloning primers*** |  |  |
|  | ***Generation of novel GoldenBraid plasmids*** |  |  |
| dB1a-extF | ATTAGAGATCTTGGCAGGATATATTGTGGTGTAAAGAATTCGTCTCA | 47 |  |
| Alfa12-F | GGTGTAAAGAATTCGTCTCACCATGGAGAGAGACCCACAGCTTGT | 45 |  |
| Alfa13-F | GGTGTAAAGAATTCGTCTCAGCTTGGAGAGAGACCCACAGCTTGT | 45 |  |
| Alfa14-F | GGTGTAAAGAATTCGTCTCAGGTAGGAGAGAGACCCACAGCTTGT | 45 |  |
| Alfa11-R | TCCTGTCAGAATTCGTCTCAATGGAGCGAGAGACCCCAGCTGGCA | 45 |  |
| Alfa12-R | TCCTGTCAGAATTCGTCTCAAAGCAGCGAGAGACCCCAGCTGGCA | 45 |  |
| Alfa13-R | TCCTGTCAGAATTCGTCTCATACCAGCGAGAGACCCCAGCTGGCA | 45 |  |
| Alfa13R-F | GGTGTAAAGAATTCGGTCTCAGCTTAGCGAGAGACCCACAGCTTGT | 45 |  |
| Alfa13R-R | TCCTGTCAGAATTCGTCTCATACCGGAGAGAGACCCCAGCTGGCA | 45 |  |
| dB1a-extR | GCGACTTAGTTTACCCGCCAATATATCCTGTCAGAATTCGTCTCA | 45 |  |
|  |  |  |  |
|  |  |  |  |
|  | ***Domestication of BeYDV*** |  |  |
| GB2-Lir1-F | GCGCCGTCTCGCTCGGGAGGTGACTCCGAGGGGTTGCCT | 39 |  |
| GB2-Lir1-R | GCGCCGTCTCGCTCAAGCGAGCAAATACCATCACATCGTATAT | 43 |  |
| GB2-Lir2-F | GCGCCGTCTCGCTCGGGAGAAACTCTAGTCAATACCATCACAT | 43 |  |
| GB2-Lir2-R | GCGCCGTCTCGCTCACATTTTGTTGTGACTCCGAGGGGT | 39 |  |
| GB2-Rep1-F | GCGCCGTCTCGCTCGAATGCCTTCTGCTAGTAAGAAC | 37 |  |
| GB2-Rep1-R | GCGCCGTCTCGATACCTTATGATCTCGGAAATCTC | 35 |  |
| GB2-Rep2-F | GCGCCGTCTCGGTATCTCCTCGCAAATCTGAC | 32 |  |
| GB2-Rep2-R | GCGCCGTCTCGCTCAAAGCTCAGTGACTCGACGATTCCC | 39 |  |
| GB2-SIR-F | GCGCCGTCTCGCTCGGCTTAACGTGCCTCTCCTCATACG | 39 |  |
| GB2-SIR-R | GCGCCGTCTCGCTCAAGCGAATGATTATTTTATGAATATATTTCATTGTGC | 51 |  |
|  |  |  |  |
|  | ***Domestication of CPMV NTRs*** |  |  |
| GB2-pEAQ1F | GCGCCGTCTCGCTCGGGAGGGAAACCTCCTCGGATTCCA | 39 |  |
| GB2-pEAQ1ATGR | GCGCCGTCTCGCTCGCATTACCGGTCGCGAATTTGGGCA | 39 |  |
| GB2-pEAQ1CCATR | GCGCCGTCTCGCTCGATGGACCGGTCGCGAATTTGGGCA | 39 |  |
| GB2-pEAQ2F1 | GCGCCGTCTCGCTCGGCTTCCCGGGCATCACCATCACCA | 39 |  |
| GB2-pEAQ2R1 | GCGCCGTCTCGCGTCGCAAGACCGGCAACAG | 31 |  |
| GB2-pEAQ2F2 | GCGCCGTCTCGGACGATTATCATATAATTTCTGTTGA | 37 |  |
| GB2-pEAQ2R2 | GCGCCGTCTCGCTCGAGCGGATCTAGTAACATAGATGACACC | 42 |  |
|  |  |  |  |
|  | ***Domestication of MAR sequences*** |  |  |
| GB2-NtRB7F | GCGCCGTCTCGCTCGGGAGTCGATTAAAAATCCCAATTATATTTGG | 46 |  |
| GB2-NtRB7R | GCGCCGTCTCGCTCGAGCGACTATTTTCAGAAGAAGTTCCCAA | 43 |  |
| GB2-NtTM2F | GCGCCGTCTCGCTCGGGAGTCGATTAAAAATCCCAATTATTGCG | 44 |  |
| GB2-NtTM2R | GCGCCGTCTCGCTCGAGCGAGGAAGAAGTTCCCAATCTTG | 40 |  |
| GB2-GmP1MARF | GCGCCGTCTCGCTCGGGAGGTGAATTCTAAACTTATATTGAGATATTA | 48 |  |
| GB2-GmP1MARR | GCGCCGTCTCGCTCGAGCGAAGCATTCATCGATGAGGGC | 39 |  |
| GB2-NtTM6F | GCGCCGTCTCGCTCGGGAGTAATATTTAGAAATTTAATTAACATAACCAAG | 51 |  |
| GB2-NtTM6R | GCGCCGTCTCGCTCGAGCGGACATCCTAGGTTCAATCAAATTT | 43 |  |
|  |  |  |  |
|  |  |  |  |
|  |  |  |  |
|  | ***Sequencing primers*** |  |  |
|  | ***Inserts cloned in alpha plasmids*** |  |  |
| GB-alfaF2 | GGGCTTCTGGATTTCCGATC | 20 |  |
| GB-alfaR2 | TTTTGCTCACATGAGATCTC | 20 |  |
|  | ***Inserts cloned in pUPD2 plasmids*** |  |  |
| pUDP2-R2 | GAGGAAGCCTGCATAACG | 18 |  |
| pUPD2-F2 | CCCGATCAACTCGAGTGCCA | 20 |  |
|  | ***Cassettes cloned next to MAR sequence*** |  |  |
| TM6-MAR-5 | GGATACCATATTAGAGTTATTACCG | 25 |  |
| TM6-MAR-3 | TTGCAACAATTACACAAGGA | 20 |  |
| TM2-MAR-5 | TATTGAGCAGCTTTCACTCG | 20 |  |
| TM2-MAR-3 | GGCGGCACAAAAGTAGAGGA | 20 |  |
| P1-MAR-3 | ATTTGGGTAAAATGTGAGAG | 20 |  |
| P1-MAR-5 | AAGAGAGTGCGGAAATTATA | 20 |  |
| RB7-MAR-3 | AAAGAATGGCAGTTTTCCTT | 20 |  |
| RB7-MAR-5 | GGTTCGAATTTGTTTTACTC | 20 |  |
|  | ***ORF cloned between CPMV NTRs*** |  |  |
| pEAQ-R | CACAGAAAACCGCTCACC | 20 |  |
| pEAQ-F | TTCTTCTTCTTGCTGATTGG | 20 |  |
|  |  |  |  |
|  |  |  |  |
|  | ***Primers for qPCR*** |  |  |
| DsRed_qPCR_F | TATATGTCAAGCACCCTGCC | 20 |  |
| DsRed_qPCR_R | CCATCGGAAGGAAAGTTCAC | 20 |  |
|  |  |  |  |
| Nb_actinS | CCGTGGAGAAGAGCTACGAG | 20 |  |
| Nb_actinAS | GATACGGGGAGCTAATGCAG | 20 |  |

**Supplementary Table 2: DNA sequence of presented constructs:**

GFP reporter gene

Dsred reporter gene

GUS reporter gene

P 19 - silencing suppressor

LIR1 – long intergenic region

LIR2 – long intergenic region

Rep – replicase derived from BeYDV

SIR – short intergenic region

**pGB-R-DsRed-GFP**

cgctgtcatgagaccggatcctgacaggatatattggcgggtaaacctaagagaaaagagcgtttattagaataatcggatatttaaaagggcgtgaaaaggtttatccgttcgtccatttgtatgtgcatgccaaccacagggttcccctcgggatcaaagtactttgatccaacccctccgctgctatagtgcagtcggcttctgacgttcagtgcagccgtcatctgaaaacgacatgtcgcacaagtcctaagttacgcgacaggctgccgccctgcccttttcctggcgttttcttgtcgcgtgttttagtcgcataaagtagaatacttgcgactagaaccggagacattacgccatgaacaagagcgccgccgctggcctgctgggctatgcccgcgtcagcaccgacgaccaggacttgaccaaccaacgggccgaactgcacgcggccggctgcaccaagctgttttccgagaagatcaccggcaccaggcgcgaccgcccggagctggccaggatgcttgaccacctacgccctggcgacgttgtgacagtgaccaggctagaccgcctggcccgcagcacccgcgacctactggacattgccgagcgcatccaggaggccggcgcgggcctgcgtagcctggcagagccgtgggccgacaccaccacgccggccggccgcatggtgttgaccgtgttcgccggcattgccgagttcgagcgttccctaatcatcgaccgcacccggagcgggcgcgaggccgccaaggcccgaggcgtgaagtttggcccccgccctaccctcaccccggcacagatcgcgcacgcccgcgagctgatcgaccaggaaggccgcaccgtgaaagaggcggctgcactgcttggcgtgcatcgctcgaccctgtaccgcgcacttgagcgcagcgaggaagtgacgcccaccgaggccaggcggcgcggtgccttccgtgaggacgcattgaccgaggccgacgccctggcggccgccgagaatgaacgccaagaggaacaagcatgaaaccgcaccaggacggccaggacgaaccgtttttcattaccgaagagatcgaggcggagatgatcgcggccgggtacgtgttcgagccgcccgcgcacctctcaaccgtgcggctgcatgaaatcctggccggtttgtctgatgccaagctggcggcctggccggccagcttggccgctgaagaaaccgagcgccgccgtctaaaaaggtgatgtgtatttgagtaaaacagcttgcgtcatgcggtcgctgcgtatatgatccgatgagtaaataaacaaatacgcaaggggaacgcatgaaggttatcgctgtacttaaccagaaaggcgggtcaggcaagacgaccatcggaacccatctagcccgcgccctgcaactcgccggggccgatgttctgttagtcgattccgatccccagggcagtgcccgcgattgggcggccgtgcgggaagatcaaccgctaaccgttgtcggcatcgaccgcccgacgattgaccgcgacgtgaaggccatcggccggcgcgacttcgtagtgatcgacggagcgccccaggcggcggacttggctgtgtccgcgatcaaggcagccgacttcgtgctgattccggtgcagccaagcccttacgacatatgggccaccgccgacctggtggagctggttaagcagcgcattgaggtcacggatggaaggctacaagcggcctttgtcgtgtcgcgggcgatcaaaggcacgcgcatcggcggtgaggttgccgaggcgctggccgggtacgagctgcccattcttgagtcccgtatcacgcagcgcgtgagctacccaggcactgccgccgccggcacaaccgttcttgaatcagaacccgagggcgacgctgcccgcgaggtccaggcgctggccgctgaaattaaatcaaaactcatttgagttaatgaggtaaagagaaaatgagcaaaagcacaaacacgctaagtgccggccgtccgagcgcacgcagcagcaaggctgcaacgttggccagcctggcagacacgccagccatgaagcgggtcaactttcagttgccggcggaggatcacaccaagctgaagatgtacgcggtacgccaaggcaagaccattaccgagctgctatctgaatagatcgcgcagctaccagagtaaatgagcaaatgaataaatgagtagatgaattttagcggctaaaggaggcggcatggaaaatcaagaacaaccaggcaccgacgccgtggaatgccccatgtgtggaggaacgggcggttggccaggcgtaagcggctgggttgtctgccggccctgcaatggcactggaacccccaagcccgaggaatcggcgtgacggtcgcaaaccatccggcccggtacaaatcggcgcggcgctgggtgatgacctggtggagaagttgaaggccgcgcaggccgcccagcggcaacgcatcgaggcagaagcacgccccggtgaatcgtggcaagcggccgctgatcgaatccgcaaagaatcccggcaaccgccggcagccggtgcgccgtcgattaggaagccgcccaagggcgacgagcaaccagattttttcgttccgatgctctatgacgtgggcacccgcgatagtcgcagcatcatggacgtggccgttttccgtctgtcgaagcgtgaccgacgagctggcgaggtgatccgctacgagcttccagacgggcacgtagaggtttccgcagggccggccggcatggccagtgtgtgggattacgacctggtactgatggcggtttcccatctaaccgaatccatgaaccgataccgggaagggaagggagacaagcccggccgcgtgttccgtccacacgttgcggacgtactcaagttctgccggcgagccgatggcggaaagcagaaagacgacctggtagaaacctgcattcggttaaacaccacgcacgttgccatgcagcgtacgaagaaggccaagaacggccgcctggtgacggtatccgagggtgaagccttgattagccgctacaagatcgtaaagagcgaaaccgggcggccggagtacatcgagatcgagctagctgattggatgtaccgcgagatcacagaaggcaagaacccggacgtgctgacggttcaccccgattactttttgatcgatcccggcatcggccgttttctctaccgcctggcacgccgcgccgcaggcaaggcagaagccagatggttgttcaagacgatctacgaacgcagtggcagcgccggagagttcaagaagttctgtttcaccgtgcgcaagctgatcgggtcaaatgacctgccggagtacgatttgaaggaggaggcggggcaggctggcccgatcctagtcatgcgctaccgcaacctgatcgagggcgaagcatccgccggttcctaatgtacggagcagatgctagggcaaattgccctagcaggggaaaaaggtcgaaaaggactctttcctgtggatagcacgtacattgggaacccaaagccgtacattgggaaccggaacccgtacattgggaacccaaagccgtacattgggaaccggtcacacatgtaagtgactgatataaaagagaaaaaaggcgatttttccgcctaaaactctttaaaacttattaaaactcttaaaacccgcctggcctgtgcataactgtctggccagcgcacagccgaagagctgcaaaaagcgcctacccttcggtcgctgcgctccctacgccccgccgcttcgcgtcggcctatcgcggccgctggccgctcaaaaatggctggcctacggccaggcaatctaccagggcgcggacaagccgcgccgtcgccactcgaccgccggcgcccacatcaaggcaccctgcctcgcgcgtttcggtgatgacggtgaaaacctctgacacatgcagctcccggtgacggtcacagcttgtctgtaagcggatgccgggagcagacaagcccgtcagggcgcgtcagcgggtgttggcgggtgtcggggcgcagccatgacccagtcacgtagcgatagcggagtgtatactggcttaactatgcggcatcagagcagattgtactgagagtgcaccatatgcggtgtgaaataccgcacagatgcgtaaggagaaaataccgcatcaggcgctcttccgcttcctcgctcactgactcgctgcgctcggtcgttcggctgcggcgagcggtatcagctcactcaaaggcggtaatacggttatccacagaatcaggggataacgcaggaaagaacatgtgagcaaaaggccagcaaaaggccaggaaccgtaaaaaggccgcgttgctggcgtttttccataggctccgcccccctgacgagcatcacaaaaatcgacgctcaagtcagaggtggcgaaacccgacaggactataaagataccaggcgtttccccctggaagctccctcgtgcgctctcctgttccgaccctgccgcttaccggatacctgtccgcctttctcccttcgggaagcgtggcgctttctcatagctcacgctgtaggtatctcagttcggtgtaggtcgttcgctccaagctgggctgtgtgcacgaaccccccgttcagcccgaccgctgcgccttatccggtaactatcgtcttgagtccaacccggtaagacacgacttatcgccactggcagcagccactggtaacaggattagcagagcgaggtatgtaggcggtgctacagagttcttgaagtggtggcctaactacggctacactagaaggacagtatttggtatctgcgctctgctgaagccagttaccttcggaaaaagagttggtagctcttgatccggcaaacaaaccaccgctggtagcggtggtttttttgtttgcaagcagcagattacgcgcagaaaaaaaggatctcaagaagatcctttgatcttttctacggggtctgacgctcagtggaacgaaaactcacgttaagggattttggtcatgcattctaggtgattatttgccgactaccttggtgatctcgcctttcacgtagtggacaaattcttccaactgatctgcgcgcgaggccaagcgatcttcttcttgtccaagataagcctgtctagcttcaagtatgacgggctgatactgggccggcaggcgctccattgcccagtcggcagcgacatccttcggcgcgattttgccggttactgcgctgtaccaaatgcgggacaacgtaagcactacatttcgctcatcaccagcccagtcgggcggcgagttccatagcgttaaggtttcatttagcgcctcaaatagatcctgttcaggaaccggatcaaagagttcctccgccgctggacctaccaaggcaacgctatgttctcttgcttttgtcagcaagatagccagatcaatgtcgatcgtggctggctcgaagatacctgcaagaatgtcattgcgctgccattctccaaattgcagttcgcgcttagctggataacgccacggaatgatgtcgtcgtgcacaacaatggtgacttctacagcgcggagaatctcgctctctccaggggaagccgaagtttccaaaaggtcgttgatcaaagctcgccgcgttgtttcatcaagccttacggtcaccgtaaccagcaaatcaatatcactgtgtggcttcaggccgccatccactgcggagccgtacaaatgtacggccagcaacgtcggttcgagatggcgctcgatgacgccaactacctctgatagttgagtcgatacttcggcgatcaccgcttccctcataatgtttaactttgttttagggcgactgccctgctgcgtaacatcgttgctgctccataacatcaaacatcgacccacggcgtaacgcgcttgctgcttggatgcccgaggcatagactgtaccccaaaaaaacagtcataacaagccatgaaaaccgccactgcgccgttaccaccgctgcgttcggtcaaggttctggaccagttgcgtgagcgcatacgctacttgcattacagcttacgaaccgaacaggcttatgtccactgggttcgtgccttcatccgtttccacggtgtgcgtcacccggcaaccttgggtagcagcgaagtcgaggcatttctgtcctggctggaacagaacttattatttccttcctcttttctacagtatttaaagataccccaagaagctaattataacaagacgaactccaattcactgttccttgcattctaaaaccttaaataccagaaaacagctttttcaaagttgttttcaaagttggcgtataacatagtatcgacggagccgattttgaaaccgcggtgatcacaggcagcaacgctctgtcatcgttacaatcaacatgctaccctccgcgagatcatccgtgtttcaaacccggcagcttagttgccgttcttccgaatagcatcggtaacatgagcaaagtctgccgccttacaacggctctcccgctgacgccgtcccggactgatgggctgcctgtatcgagtggtgattttgtgccgagctgccggtcggggagctgttggctggctggtggcaggatatattgtggtgtaaacataacggatccggtctcaggagggagggaggtcaacatggtggagcacgacactctggtctactccaaaaatgtcaaagatacagtctcagaagatcaaagggctattgagacttttcaacaaaggataatttcgggaaacctcctcggattccattgcccagctatctgtcacttcatcgaaaggacagtagaaaaggaaggtggctcctacaaatgccatcattgcgataaaggaaaggctatcattcaagatctctctgccgacagtggtcccaaagatggacccccacccacgaggagcatcgtggaaaaagaagaggttccaaccacgtctacaaagcaagtggattgatgtgacatctccactgacgtaagggatgacgcacaatcccactatccttcgcaagacccttcctctatataaggaagttcatttcatttggagaggacacgctcgagtataagagctcatttttacaacaattaccaacaacaacaaacaacaaacaacattacaattacatttacaattatcgatacaatggaacgagctatacaaggaaacgacgctagggaacaagctaacagtgaacgttgggatggaggatcaggaggtaccacttctcccttcaaacttcctgacgaaagtccgagttggactgagtggcggctacataacgatgagactaattcgaatcaagataatccccttggtttcaaggaaagctggggtttcgggaaagttgtatttaagagatatctcagatacgacaggacggaagcttcactgcacagagtccttggatcttggacgggagattcggttaactatgcagcatctcgatttttcggtttcgaccagatcggatgtacctatagtattcggtttcgaggagttagtatcaccgtttctggaggctctcgaactcttcagcatctctgtgagatggcaattcggtctaagcaagaactgctacagcttgccccaatcgaagtggaaagtaatgtatcaagaggatgccctgaaggtactgaaaccttcgaaaaagaaagcgagtgagcttgtcaagcagatcgttcaaacatttggcaataaagtttcttaagattgaatcctgttgccggtcttgcgatgattatcatataatttctgttgaattacgttaagcatgtaataattaacatgtaatgcatgacgttatttatgagatgggtttttatgattagagtcccgcaattatacatttaatacgcgatagaaaacaaaatatagcgcgcaaactaggataaattatcgcgcgcggtgtcatctatgttactagatcgacgctccatggaggtgactccgaggggttgcctcaaactctatcttataaccggcgtggaggcatggaggcaggggtattttggtcattttaatagatagtggaaaatgacgtggaatttacttaaagacgaagtctttgcgacaagggggggcccacgccgaatttaatattaccggcgtggcccccccttatcgcgagtgctttagcacgagcggtccagatttaaagtagaaaatttcccgcccactagggttaaaggtgttcacactataaaagcatatacgatgtgatggtatttgctcgctgcttggagggaaacctcctcggattccattgcccagctatctgtcactttattgagaagatagtggaaaaggaaggtggctcctacaaatgccatcattgcgataaaggaaaggccatcgttgaagatgcctctgccgacagtggtcccaaagatggacccccacccacgaggagcatcgtggaaaaagaagacgttccaaccacgtcttcaaagcaagtggattgatgtgatatctccactgacgtaagggatgacgcacaatcccactatccttcgcaagacccttcctctatataaggaagttcatttcatttggagaggtattaaaatcttaataggttttgataaaagcgaacgtggggaaacccgaaccaaaccttcttctaaactctctctcatctctcttaaagcaaacttctctcttgtctttcttgcgtgagcgatcttcaacgttgtcagatcgtgcttcggcaccagtacaacgttttctttcactgaagcgaaatcaaagatctctttgtggacacgtagtgcggcgccattaaataacgtgtacttgtcctattcttgtcggtgtggtcttgggaaaagaaagcttgctggaggctgctgttcagccccatacattacttgttacgattctgctgactttcggcgggtgcaatatctctacttctgcttgacgaggtattgttgcctgtacttctttcttcttcttcttgctgattggttctataagaaatctagtattttctttgaaacagagttttcccgtggttttcgaacttggagaaagattgttaagcttctgtatattctgcccaaattcgcgaccggtaatggggtcatccaagaatgttatcaaggagttcatgaggtttaaggttcgcatggaaggaacggtcaatgggcacgagtttgaaatagaaggcgaaggagaggggaggccatacgaaggccacaataccgtaaagcttaaggtaaccaaggggggacctttgccatttgcttgggatattttgtcaccacaatttcagtatggaagcaaggtatatgtcaagcaccctgccgacataccagactataaaaagctgtcatttcctgaaggatttaaatgggaaagggtcatgaactttgaagatggtggcgtcgttactgtaacccaggattccagtttgcaggatggctgtttcatctacaaggtcaagttcattggcgtgaactttccttccgatggacctgttatgcaaaagaaaacaatgggctgggaagccagcactgagcgtttgtatcctcgtgatggcgtgttgaaaggagagattcataaggctctgaagctgaaagacggtggtcattacctagttgaattcaaaagtatttacatggcaaagaagcctgtgcagctaccagggtactactatgttgactccaaactggatataacaagccacaacgaagattatacaatcgttgagcagtatgaaagaaccgagggacgccaccatctgttcctttaagctttcccgggcatcaccatcaccatcactagctcgaggcctttaactctggtttcattaaattttctttagtttgaatttactgttattcggtgtgcatttctatgtttggtgagcggttttctgtgctcagagtgtgtttattttatgtaatttaatttctttgtgagctcctgtttagcaggtcgtcccttcagcaaggacacaaaaagattttaattttattaaaaaaaaaaaaaaaaaagaccgggaattcgatatcaagcttatcgacctgcagatcgttcaaacatttggcaataaagtttcttaagattgaatcctgttgccggtcttgcggtgattatcatataatttctgttgaattacgttaagcatgtaataattaacatgtaatgcatgacgttatttatgagatgggtttttatgattagagtcccgcaattatacatttaatacgcgatagaaaacaaaatatagcgcgcaaactaggataaattatcgcgcgcggtgtcatctatgttactagatccgctggtaggagggaaacctcctcggattccattgcccagctatctgtcactttattgagaagatagtggaaaaggaaggtggctcctacaaatgccatcattgcgataaaggaaaggccatcgttgaagatgcctctgccgacagtggtcccaaagatggacccccacccacgaggagcatcgtggaaaaagaagacgttccaaccacgtcttcaaagcaagtggattgatgtgatatctccactgacgtaagggatgacgcacaatcccactatccttcgcaagacccttcctctatataaggaagttcatttcatttggagaggtattaaaatcttaataggttttgataaaagcgaacgtggggaaacccgaaccaaaccttcttctaaactctctctcatctctcttaaagcaaacttctctcttgtctttcttgcgtgagcgatcttcaacgttgtcagatcgtgcttcggcaccagtacaacgttttctttcactgaagcgaaatcaaagatctctttgtggacacgtagtgcggcgccattaaataacgtgtacttgtcctattcttgtcggtgtggtcttgggaaaagaaagcttgctggaggctgctgttcagccccatacattacttgttacgattctgctgactttcggcgggtgcaatatctctacttctgcttgacgaggtattgttgcctgtacttctttcttcttcttcttgctgattggttctataagaaatctagtattttctttgaaacagagttttcccgtggttttcgaacttggagaaagattgttaagcttctgtatattctgcccaaattcgcgaccggtaatggtgagcaagggcgaggagctgttcaccggggtggtgcccatcctggtcgagctggacggcgacgtaaacggccacaagttcagcgtgtccggcgagggcgagggcgatgccacctacggcaagctgaccctgaagttcatctgcaccaccggcaagctgcccgtgccctggcccaccctcgtgaccaccttcagctacggcgtgcagtgcttcagccgctaccccgaccacatgaagcagcacgacttcttcaagtccgccatgcccgaaggctacgtccaggagcgcaccatcttcttcaaggacgacggcaactacaagacccgcgccgaggtgaagttcgagggcgacaccctggtgaaccgcatcgagctgaagggcatcgacttcaaggaggacggcaacatcctggggcacaagctggagtacaactacaacagccacaacgtctatatcatggccgacaagcagaagaacggcatcaaggtgaacttcaagatccgccacaacatcgaggacggcagcgtgcagctcgccgaccactaccagcagaacacccccatcggcgacggccccgtgctgctgcccgacaaccactacctgagcacccagtccgccctgagcaaagaccccaacgagaagcgcgatcacatggtcctgctggagttcgtgaccgccgccgggatcactcacggcatggacgagctgtacaagtaagctttcccgggcatcaccatcaccatcactagctcgaggcctttaactctggtttcattaaattttctttagtttgaatttactgttattcggtgtgcatttctatgtttggtgagcggttttctgtgctcagagtgtgtttattttatgtaatttaatttctttgtgagctcctgtttagcaggtcgtcccttcagcaaggacacaaaaagattttaattttattaaaaaaaaaaaaaaaaaagaccgggaattcgatatcaagcttatcgacctgcagatcgttcaaacatttggcaataaagtttcttaagattgaatcctgttgccggtcttgcggtgattatcatataatttctgttgaattacgttaagcatgtaataattaacatgtaatgcatgacgttatttatgagatgggtttttatgattagagtcccgcaattatacatttaatacgcgatagaaaacaaaatatagcgcgcaaactaggataaattatcgcgcgcggtgtcatctatgttactagatccgctgtcaaaactctagtcaataccatcacatcgtatatgcttgtatagtgtgaacacctttaaccctagtgggcgggaaattttctactttaaatctggaccgctcgtgctaaagcactcgcgataagggggggccacgccggtaatattaaattcggcgtgggcccccccttgtcgcaaagacttcgtctttaagtaaattccacgtcattttccactatctattaaaatgaccaaaatacccctgcctccatgcctccacgccggttataagatagagtttgaggcaacccctcggagtcacaacaaatgccttctgctagtaagaacttcagactccaatctaaatatgttttccttacctatcccaagtgctcatctcaaagagatgatttattccagtttctctgggagaaactcacaccttttcttattttcttccttggtgttgcttctgagcttcatcaagatggcactacccactatcatgctcttctccagcttgataaaaaaccttgtattagggatccttcttttttcgattttgaaggaaatcaccctaatatccagccagctagaaactctaaacaagtccttgattacatatcaaaggacggagatattaaaaccagaggagatttccgagatcataaggtatctcctcgcaaatctgacgcacgatggagaactattatccagactgcaacgtctaaggaggaatatcttgacatgatcaaggaagaattccctcatgaatgggcaacaaaacttcaatggctggaatattcagccaacaaattattccctccacaacctgaaccgtatgtgtcgcccttcacagaatcagatcttcgctgccacgaagatctacactcctggagggaaacccatctataccatgtaagcatagacgcttatacttacatacatcctgtctcataccaacaagctcaatctgaccttgaatggatggccgatttaaccaggacaatggaaggaatggaatccgacaccccagcctctacatctgcggaccaactcgtaccggaaagaccacctgggctagaagtctcggacgacacaactattggaacggtaccatcgatttcaccaactacgatgaacacgccacctataatatcatcgacgacatccccttcaagttcgtcccattgtggaagcaattaataggttgccagtctgatttcactgtcaaccctaaatatggaaaaaagaagaaaataaaaggtgggatcccttctataattctttgcaatcctgacgaagactggatgttatcaatgacaagtcaacagaaggattactttaaagataattgcgtcacccactatatgtgtgacggggagactttttttgctcgggaatcgtcgagtcactgaaacgtgcctctcctcatacgagtttatctaaagtgattattttttttggggtgtttgtttttggattgtctttttttgtttatttcgtgtgttatgtaacatatgtaatttctatctacttgcacaatgaaatatattcataaaataatcatt

**pGB-R-GFP-DsRed**

cgctgtcatgagaccggatcctgacaggatatattggcgggtaaacctaagagaaaagagcgtttattagaataatcggatatttaaaagggcgtgaaaaggtttatccgttcgtccatttgtatgtgcatgccaaccacagggttcccctcgggatcaaagtactttgatccaacccctccgctgctatagtgcagtcggcttctgacgttcagtgcagccgtcatctgaaaacgacatgtcgcacaagtcctaagttacgcgacaggctgccgccctgcccttttcctggcgttttcttgtcgcgtgttttagtcgcataaagtagaatacttgcgactagaaccggagacattacgccatgaacaagagcgccgccgctggcctgctgggctatgcccgcgtcagcaccgacgaccaggacttgaccaaccaacgggccgaactgcacgcggccggctgcaccaagctgttttccgagaagatcaccggcaccaggcgcgaccgcccggagctggccaggatgcttgaccacctacgccctggcgacgttgtgacagtgaccaggctagaccgcctggcccgcagcacccgcgacctactggacattgccgagcgcatccaggaggccggcgcgggcctgcgtagcctggcagagccgtgggccgacaccaccacgccggccggccgcatggtgttgaccgtgttcgccggcattgccgagttcgagcgttccctaatcatcgaccgcacccggagcgggcgcgaggccgccaaggcccgaggcgtgaagtttggcccccgccctaccctcaccccggcacagatcgcgcacgcccgcgagctgatcgaccaggaaggccgcaccgtgaaagaggcggctgcactgcttggcgtgcatcgctcgaccctgtaccgcgcacttgagcgcagcgaggaagtgacgcccaccgaggccaggcggcgcggtgccttccgtgaggacgcattgaccgaggccgacgccctggcggccgccgagaatgaacgccaagaggaacaagcatgaaaccgcaccaggacggccaggacgaaccgtttttcattaccgaagagatcgaggcggagatgatcgcggccgggtacgtgttcgagccgcccgcgcacctctcaaccgtgcggctgcatgaaatcctggccggtttgtctgatgccaagctggcggcctggccggccagcttggccgctgaagaaaccgagcgccgccgtctaaaaaggtgatgtgtatttgagtaaaacagcttgcgtcatgcggtcgctgcgtatatgatccgatgagtaaataaacaaatacgcaaggggaacgcatgaaggttatcgctgtacttaaccagaaaggcgggtcaggcaagacgaccatcggaacccatctagcccgcgccctgcaactcgccggggccgatgttctgttagtcgattccgatccccagggcagtgcccgcgattgggcggccgtgcgggaagatcaaccgctaaccgttgtcggcatcgaccgcccgacgattgaccgcgacgtgaaggccatcggccggcgcgacttcgtagtgatcgacggagcgccccaggcggcggacttggctgtgtccgcgatcaaggcagccgacttcgtgctgattccggtgcagccaagcccttacgacatatgggccaccgccgacctggtggagctggttaagcagcgcattgaggtcacggatggaaggctacaagcggcctttgtcgtgtcgcgggcgatcaaaggcacgcgcatcggcggtgaggttgccgaggcgctggccgggtacgagctgcccattcttgagtcccgtatcacgcagcgcgtgagctacccaggcactgccgccgccggcacaaccgttcttgaatcagaacccgagggcgacgctgcccgcgaggtccaggcgctggccgctgaaattaaatcaaaactcatttgagttaatgaggtaaagagaaaatgagcaaaagcacaaacacgctaagtgccggccgtccgagcgcacgcagcagcaaggctgcaacgttggccagcctggcagacacgccagccatgaagcgggtcaactttcagttgccggcggaggatcacaccaagctgaagatgtacgcggtacgccaaggcaagaccattaccgagctgctatctgaatagatcgcgcagctaccagagtaaatgagcaaatgaataaatgagtagatgaattttagcggctaaaggaggcggcatggaaaatcaagaacaaccaggcaccgacgccgtggaatgccccatgtgtggaggaacgggcggttggccaggcgtaagcggctgggttgtctgccggccctgcaatggcactggaacccccaagcccgaggaatcggcgtgacggtcgcaaaccatccggcccggtacaaatcggcgcggcgctgggtgatgacctggtggagaagttgaaggccgcgcaggccgcccagcggcaacgcatcgaggcagaagcacgccccggtgaatcgtggcaagcggccgctgatcgaatccgcaaagaatcccggcaaccgccggcagccggtgcgccgtcgattaggaagccgcccaagggcgacgagcaaccagattttttcgttccgatgctctatgacgtgggcacccgcgatagtcgcagcatcatggacgtggccgttttccgtctgtcgaagcgtgaccgacgagctggcgaggtgatccgctacgagcttccagacgggcacgtagaggtttccgcagggccggccggcatggccagtgtgtgggattacgacctggtactgatggcggtttcccatctaaccgaatccatgaaccgataccgggaagggaagggagacaagcccggccgcgtgttccgtccacacgttgcggacgtactcaagttctgccggcgagccgatggcggaaagcagaaagacgacctggtagaaacctgcattcggttaaacaccacgcacgttgccatgcagcgtacgaagaaggccaagaacggccgcctggtgacggtatccgagggtgaagccttgattagccgctacaagatcgtaaagagcgaaaccgggcggccggagtacatcgagatcgagctagctgattggatgtaccgcgagatcacagaaggcaagaacccggacgtgctgacggttcaccccgattactttttgatcgatcccggcatcggccgttttctctaccgcctggcacgccgcgccgcaggcaaggcagaagccagatggttgttcaagacgatctacgaacgcagtggcagcgccggagagttcaagaagttctgtttcaccgtgcgcaagctgatcgggtcaaatgacctgccggagtacgatttgaaggaggaggcggggcaggctggcccgatcctagtcatgcgctaccgcaacctgatcgagggcgaagcatccgccggttcctaatgtacggagcagatgctagggcaaattgccctagcaggggaaaaaggtcgaaaaggactctttcctgtggatagcacgtacattgggaacccaaagccgtacattgggaaccggaacccgtacattgggaacccaaagccgtacattgggaaccggtcacacatgtaagtgactgatataaaagagaaaaaaggcgatttttccgcctaaaactctttaaaacttattaaaactcttaaaacccgcctggcctgtgcataactgtctggccagcgcacagccgaagagctgcaaaaagcgcctacccttcggtcgctgcgctccctacgccccgccgcttcgcgtcggcctatcgcggccgctggccgctcaaaaatggctggcctacggccaggcaatctaccagggcgcggacaagccgcgccgtcgccactcgaccgccggcgcccacatcaaggcaccctgcctcgcgcgtttcggtgatgacggtgaaaacctctgacacatgcagctcccggtgacggtcacagcttgtctgtaagcggatgccgggagcagacaagcccgtcagggcgcgtcagcgggtgttggcgggtgtcggggcgcagccatgacccagtcacgtagcgatagcggagtgtatactggcttaactatgcggcatcagagcagattgtactgagagtgcaccatatgcggtgtgaaataccgcacagatgcgtaaggagaaaataccgcatcaggcgctcttccgcttcctcgctcactgactcgctgcgctcggtcgttcggctgcggcgagcggtatcagctcactcaaaggcggtaatacggttatccacagaatcaggggataacgcaggaaagaacatgtgagcaaaaggccagcaaaaggccaggaaccgtaaaaaggccgcgttgctggcgtttttccataggctccgcccccctgacgagcatcacaaaaatcgacgctcaagtcagaggtggcgaaacccgacaggactataaagataccaggcgtttccccctggaagctccctcgtgcgctctcctgttccgaccctgccgcttaccggatacctgtccgcctttctcccttcgggaagcgtggcgctttctcatagctcacgctgtaggtatctcagttcggtgtaggtcgttcgctccaagctgggctgtgtgcacgaaccccccgttcagcccgaccgctgcgccttatccggtaactatcgtcttgagtccaacccggtaagacacgacttatcgccactggcagcagccactggtaacaggattagcagagcgaggtatgtaggcggtgctacagagttcttgaagtggtggcctaactacggctacactagaaggacagtatttggtatctgcgctctgctgaagccagttaccttcggaaaaagagttggtagctcttgatccggcaaacaaaccaccgctggtagcggtggtttttttgtttgcaagcagcagattacgcgcagaaaaaaaggatctcaagaagatcctttgatcttttctacggggtctgacgctcagtggaacgaaaactcacgttaagggattttggtcatgcattctaggtgattatttgccgactaccttggtgatctcgcctttcacgtagtggacaaattcttccaactgatctgcgcgcgaggccaagcgatcttcttcttgtccaagataagcctgtctagcttcaagtatgacgggctgatactgggccggcaggcgctccattgcccagtcggcagcgacatccttcggcgcgattttgccggttactgcgctgtaccaaatgcgggacaacgtaagcactacatttcgctcatcaccagcccagtcgggcggcgagttccatagcgttaaggtttcatttagcgcctcaaatagatcctgttcaggaaccggatcaaagagttcctccgccgctggacctaccaaggcaacgctatgttctcttgcttttgtcagcaagatagccagatcaatgtcgatcgtggctggctcgaagatacctgcaagaatgtcattgcgctgccattctccaaattgcagttcgcgcttagctggataacgccacggaatgatgtcgtcgtgcacaacaatggtgacttctacagcgcggagaatctcgctctctccaggggaagccgaagtttccaaaaggtcgttgatcaaagctcgccgcgttgtttcatcaagccttacggtcaccgtaaccagcaaatcaatatcactgtgtggcttcaggccgccatccactgcggagccgtacaaatgtacggccagcaacgtcggttcgagatggcgctcgatgacgccaactacctctgatagttgagtcgatacttcggcgatcaccgcttccctcataatgtttaactttgttttagggcgactgccctgctgcgtaacatcgttgctgctccataacatcaaacatcgacccacggcgtaacgcgcttgctgcttggatgcccgaggcatagactgtaccccaaaaaaacagtcataacaagccatgaaaaccgccactgcgccgttaccaccgctgcgttcggtcaaggttctggaccagttgcgtgagcgcatacgctacttgcattacagcttacgaaccgaacaggcttatgtccactgggttcgtgccttcatccgtttccacggtgtgcgtcacccggcaaccttgggtagcagcgaagtcgaggcatttctgtcctggctggaacagaacttattatttccttcctcttttctacagtatttaaagataccccaagaagctaattataacaagacgaactccaattcactgttccttgcattctaaaaccttaaataccagaaaacagctttttcaaagttgttttcaaagttggcgtataacatagtatcgacggagccgattttgaaaccgcggtgatcacaggcagcaacgctctgtcatcgttacaatcaacatgctaccctccgcgagatcatccgtgtttcaaacccggcagcttagttgccgttcttccgaatagcatcggtaacatgagcaaagtctgccgccttacaacggctctcccgctgacgccgtcccggactgatgggctgcctgtatcgagtggtgattttgtgccgagctgccggtcggggagctgttggctggctggtggcaggatatattgtggtgtaaacataacggatccggtctcaggagggagggaggtcaacatggtggagcacgacactctggtctactccaaaaatgtcaaagatacagtctcagaagatcaaagggctattgagacttttcaacaaaggataatttcgggaaacctcctcggattccattgcccagctatctgtcacttcatcgaaaggacagtagaaaaggaaggtggctcctacaaatgccatcattgcgataaaggaaaggctatcattcaagatctctctgccgacagtggtcccaaagatggacccccacccacgaggagcatcgtggaaaaagaagaggttccaaccacgtctacaaagcaagtggattgatgtgacatctccactgacgtaagggatgacgcacaatcccactatccttcgcaagacccttcctctatataaggaagttcatttcatttggagaggacacgctcgagtataagagctcatttttacaacaattaccaacaacaacaaacaacaaacaacattacaattacatttacaattatcgatacaatggaacgagctatacaaggaaacgacgctagggaacaagctaacagtgaacgttgggatggaggatcaggaggtaccacttctcccttcaaacttcctgacgaaagtccgagttggactgagtggcggctacataacgatgagactaattcgaatcaagataatccccttggtttcaaggaaagctggggtttcgggaaagttgtatttaagagatatctcagatacgacaggacggaagcttcactgcacagagtccttggatcttggacgggagattcggttaactatgcagcatctcgatttttcggtttcgaccagatcggatgtacctatagtattcggtttcgaggagttagtatcaccgtttctggaggctctcgaactcttcagcatctctgtgagatggcaattcggtctaagcaagaactgctacagcttgccccaatcgaagtggaaagtaatgtatcaagaggatgccctgaaggtactgaaaccttcgaaaaagaaagcgagtgagcttgtcaagcagatcgttcaaacatttggcaataaagtttcttaagattgaatcctgttgccggtcttgcgatgattatcatataatttctgttgaattacgttaagcatgtaataattaacatgtaatgcatgacgttatttatgagatgggtttttatgattagagtcccgcaattatacatttaatacgcgatagaaaacaaaatatagcgcgcaaactaggataaattatcgcgcgcggtgtcatctatgttactagatcgacgctccatggaggtgactccgaggggttgcctcaaactctatcttataaccggcgtggaggcatggaggcaggggtattttggtcattttaatagatagtggaaaatgacgtggaatttacttaaagacgaagtctttgcgacaagggggggcccacgccgaatttaatattaccggcgtggcccccccttatcgcgagtgctttagcacgagcggtccagatttaaagtagaaaatttcccgcccactagggttaaaggtgttcacactataaaagcatatacgatgtgatggtatttgctcgctgcttggagggaaacctcctcggattccattgcccagctatctgtcactttattgagaagatagtggaaaaggaaggtggctcctacaaatgccatcattgcgataaaggaaaggccatcgttgaagatgcctctgccgacagtggtcccaaagatggacccccacccacgaggagcatcgtggaaaaagaagacgttccaaccacgtcttcaaagcaagtggattgatgtgatatctccactgacgtaagggatgacgcacaatcccactatccttcgcaagacccttcctctatataaggaagttcatttcatttggagaggtattaaaatcttaataggttttgataaaagcgaacgtggggaaacccgaaccaaaccttcttctaaactctctctcatctctcttaaagcaaacttctctcttgtctttcttgcgtgagcgatcttcaacgttgtcagatcgtgcttcggcaccagtacaacgttttctttcactgaagcgaaatcaaagatctctttgtggacacgtagtgcggcgccattaaataacgtgtacttgtcctattcttgtcggtgtggtcttgggaaaagaaagcttgctggaggctgctgttcagccccatacattacttgttacgattctgctgactttcggcgggtgcaatatctctacttctgcttgacgaggtattgttgcctgtacttctttcttcttcttcttgctgattggttctataagaaatctagtattttctttgaaacagagttttcccgtggttttcgaacttggagaaagattgttaagcttctgtatattctgcccaaattcgcgaccggtaatggtgagcaagggcgaggagctgttcaccggggtggtgcccatcctggtcgagctggacggcgacgtaaacggccacaagttcagcgtgtccggcgagggcgagggcgatgccacctacggcaagctgaccctgaagttcatctgcaccaccggcaagctgcccgtgccctggcccaccctcgtgaccaccttcagctacggcgtgcagtgcttcagccgctaccccgaccacatgaagcagcacgacttcttcaagtccgccatgcccgaaggctacgtccaggagcgcaccatcttcttcaaggacgacggcaactacaagacccgcgccgaggtgaagttcgagggcgacaccctggtgaaccgcatcgagctgaagggcatcgacttcaaggaggacggcaacatcctggggcacaagctggagtacaactacaacagccacaacgtctatatcatggccgacaagcagaagaacggcatcaaggtgaacttcaagatccgccacaacatcgaggacggcagcgtgcagctcgccgaccactaccagcagaacacccccatcggcgacggccccgtgctgctgcccgacaaccactacctgagcacccagtccgccctgagcaaagaccccaacgagaagcgcgatcacatggtcctgctggagttcgtgaccgccgccgggatcactcacggcatggacgagctgtacaagtaagctttcccgggcatcaccatcaccatcactagctcgaggcctttaactctggtttcattaaattttctttagtttgaatttactgttattcggtgtgcatttctatgtttggtgagcggttttctgtgctcagagtgtgtttattttatgtaatttaatttctttgtgagctcctgtttagcaggtcgtcccttcagcaaggacacaaaaagattttaattttattaaaaaaaaaaaaaaaaaagaccgggaattcgatatcaagcttatcgacctgcagatcgttcaaacatttggcaataaagtttcttaagattgaatcctgttgccggtcttgcggtgattatcatataatttctgttgaattacgttaagcatgtaataattaacatgtaatgcatgacgttatttatgagatgggtttttatgattagagtcccgcaattatacatttaatacgcgatagaaaacaaaatatagcgcgcaaactaggataaattatcgcgcgcggtgtcatctatgttactagatccgctggtaggagggaaacctcctcggattccattgcccagctatctgtcactttattgagaagatagtggaaaaggaaggtggctcctacaaatgccatcattgcgataaaggaaaggccatcgttgaagatgcctctgccgacagtggtcccaaagatggacccccacccacgaggagcatcgtggaaaaagaagacgttccaaccacgtcttcaaagcaagtggattgatgtgatatctccactgacgtaagggatgacgcacaatcccactatccttcgcaagacccttcctctatataaggaagttcatttcatttggagaggtattaaaatcttaataggttttgataaaagcgaacgtggggaaacccgaaccaaaccttcttctaaactctctctcatctctcttaaagcaaacttctctcttgtctttcttgcgtgagcgatcttcaacgttgtcagatcgtgcttcggcaccagtacaacgttttctttcactgaagcgaaatcaaagatctctttgtggacacgtagtgcggcgccattaaataacgtgtacttgtcctattcttgtcggtgtggtcttgggaaaagaaagcttgctggaggctgctgttcagccccatacattacttgttacgattctgctgactttcggcgggtgcaatatctctacttctgcttgacgaggtattgttgcctgtacttctttcttcttcttcttgctgattggttctataagaaatctagtattttctttgaaacagagttttcccgtggttttcgaacttggagaaagattgttaagcttctgtatattctgcccaaattcgcgaccggtaatggggtcatccaagaatgttatcaaggagttcatgaggtttaaggttcgcatggaaggaacggtcaatgggcacgagtttgaaatagaaggcgaaggagaggggaggccatacgaaggccacaataccgtaaagcttaaggtaaccaaggggggacctttgccatttgcttgggatattttgtcaccacaatttcagtatggaagcaaggtatatgtcaagcaccctgccgacataccagactataaaaagctgtcatttcctgaaggatttaaatgggaaagggtcatgaactttgaagatggtggcgtcgttactgtaacccaggattccagtttgcaggatggctgtttcatctacaaggtcaagttcattggcgtgaactttccttccgatggacctgttatgcaaaagaaaacaatgggctgggaagccagcactgagcgtttgtatcctcgtgatggcgtgttgaaaggagagattcataaggctctgaagctgaaagacggtggtcattacctagttgaattcaaaagtatttacatggcaaagaagcctgtgcagctaccagggtactactatgttgactccaaactggatataacaagccacaacgaagattatacaatcgttgagcagtatgaaagaaccgagggacgccaccatctgttcctttaagctttcccgggcatcaccatcaccatcactagctcgaggcctttaactctggtttcattaaattttctttagtttgaatttactgttattcggtgtgcatttctatgtttggtgagcggttttctgtgctcagagtgtgtttattttatgtaatttaatttctttgtgagctcctgtttagcaggtcgtcccttcagcaaggacacaaaaagattttaattttattaaaaaaaaaaaaaaaaaagaccgggaattcgatatcaagcttatcgacctgcagatcgttcaaacatttggcaataaagtttcttaagattgaatcctgttgccggtcttgcggtgattatcatataatttctgttgaattacgttaagcatgtaataattaacatgtaatgcatgacgttatttatgagatgggtttttatgattagagtcccgcaattatacatttaatacgcgatagaaaacaaaatatagcgcgcaaactaggataaattatcgcgcgcggtgtcatctatgttactagatccgctgtcaaaactctagtcaataccatcacatcgtatatgcttgtatagtgtgaacacctttaaccctagtgggcgggaaattttctactttaaatctggaccgctcgtgctaaagcactcgcgataagggggggccacgccggtaatattaaattcggcgtgggcccccccttgtcgcaaagacttcgtctttaagtaaattccacgtcattttccactatctattaaaatgaccaaaatacccctgcctccatgcctccacgccggttataagatagagtttgaggcaacccctcggagtcacaacaaatgccttctgctagtaagaacttcagactccaatctaaatatgttttccttacctatcccaagtgctcatctcaaagagatgatttattccagtttctctgggagaaactcacaccttttcttattttcttccttggtgttgcttctgagcttcatcaagatggcactacccactatcatgctcttctccagcttgataaaaaaccttgtattagggatccttcttttttcgattttgaaggaaatcaccctaatatccagccagctagaaactctaaacaagtccttgattacatatcaaaggacggagatattaaaaccagaggagatttccgagatcataaggtatctcctcgcaaatctgacgcacgatggagaactattatccagactgcaacgtctaaggaggaatatcttgacatgatcaaggaagaattccctcatgaatgggcaacaaaacttcaatggctggaatattcagccaacaaattattccctccacaacctgaaccgtatgtgtcgcccttcacagaatcagatcttcgctgccacgaagatctacactcctggagggaaacccatctataccatgtaagcatagacgcttatacttacatacatcctgtctcataccaacaagctcaatctgaccttgaatggatggccgatttaaccaggacaatggaaggaatggaatccgacaccccagcctctacatctgcggaccaactcgtaccggaaagaccacctgggctagaagtctcggacgacacaactattggaacggtaccatcgatttcaccaactacgatgaacacgccacctataatatcatcgacgacatccccttcaagttcgtcccattgtggaagcaattaataggttgccagtctgatttcactgtcaaccctaaatatggaaaaaagaagaaaataaaaggtgggatcccttctataattctttgcaatcctgacgaagactggatgttatcaatgacaagtcaacagaaggattactttaaagataattgcgtcacccactatatgtgtgacggggagactttttttgctcgggaatcgtcgagtcactgaaacgtgcctctcctcatacgagtttatctaaagtgattattttttttggggtgtttgtttttggattgtctttttttgtttatttcgtgtgttatgtaacatatgtaatttctatctacttgcacaatgaaatatattcataaaataatcatt

**pGB-G-GFP-DsRed**

cgctgtcatgagaccggatcctgacaggatatattggcgggtaaacctaagagaaaagagcgtttattagaataatcggatatttaaaagggcgtgaaaaggtttatccgttcgtccatttgtatgtgcatgccaaccacagggttcccctcgggatcaaagtactttgatccaacccctccgctgctatagtgcagtcggcttctgacgttcagtgcagccgtcatctgaaaacgacatgtcgcacaagtcctaagttacgcgacaggctgccgccctgcccttttcctggcgttttcttgtcgcgtgttttagtcgcataaagtagaatacttgcgactagaaccggagacattacgccatgaacaagagcgccgccgctggcctgctgggctatgcccgcgtcagcaccgacgaccaggacttgaccaaccaacgggccgaactgcacgcggccggctgcaccaagctgttttccgagaagatcaccggcaccaggcgcgaccgcccggagctggccaggatgcttgaccacctacgccctggcgacgttgtgacagtgaccaggctagaccgcctggcccgcagcacccgcgacctactggacattgccgagcgcatccaggaggccggcgcgggcctgcgtagcctggcagagccgtgggccgacaccaccacgccggccggccgcatggtgttgaccgtgttcgccggcattgccgagttcgagcgttccctaatcatcgaccgcacccggagcgggcgcgaggccgccaaggcccgaggcgtgaagtttggcccccgccctaccctcaccccggcacagatcgcgcacgcccgcgagctgatcgaccaggaaggccgcaccgtgaaagaggcggctgcactgcttggcgtgcatcgctcgaccctgtaccgcgcacttgagcgcagcgaggaagtgacgcccaccgaggccaggcggcgcggtgccttccgtgaggacgcattgaccgaggccgacgccctggcggccgccgagaatgaacgccaagaggaacaagcatgaaaccgcaccaggacggccaggacgaaccgtttttcattaccgaagagatcgaggcggagatgatcgcggccgggtacgtgttcgagccgcccgcgcacctctcaaccgtgcggctgcatgaaatcctggccggtttgtctgatgccaagctggcggcctggccggccagcttggccgctgaagaaaccgagcgccgccgtctaaaaaggtgatgtgtatttgagtaaaacagcttgcgtcatgcggtcgctgcgtatatgatccgatgagtaaataaacaaatacgcaaggggaacgcatgaaggttatcgctgtacttaaccagaaaggcgggtcaggcaagacgaccatcggaacccatctagcccgcgccctgcaactcgccggggccgatgttctgttagtcgattccgatccccagggcagtgcccgcgattgggcggccgtgcgggaagatcaaccgctaaccgttgtcggcatcgaccgcccgacgattgaccgcgacgtgaaggccatcggccggcgcgacttcgtagtgatcgacggagcgccccaggcggcggacttggctgtgtccgcgatcaaggcagccgacttcgtgctgattccggtgcagccaagcccttacgacatatgggccaccgccgacctggtggagctggttaagcagcgcattgaggtcacggatggaaggctacaagcggcctttgtcgtgtcgcgggcgatcaaaggcacgcgcatcggcggtgaggttgccgaggcgctggccgggtacgagctgcccattcttgagtcccgtatcacgcagcgcgtgagctacccaggcactgccgccgccggcacaaccgttcttgaatcagaacccgagggcgacgctgcccgcgaggtccaggcgctggccgctgaaattaaatcaaaactcatttgagttaatgaggtaaagagaaaatgagcaaaagcacaaacacgctaagtgccggccgtccgagcgcacgcagcagcaaggctgcaacgttggccagcctggcagacacgccagccatgaagcgggtcaactttcagttgccggcggaggatcacaccaagctgaagatgtacgcggtacgccaaggcaagaccattaccgagctgctatctgaatagatcgcgcagctaccagagtaaatgagcaaatgaataaatgagtagatgaattttagcggctaaaggaggcggcatggaaaatcaagaacaaccaggcaccgacgccgtggaatgccccatgtgtggaggaacgggcggttggccaggcgtaagcggctgggttgtctgccggccctgcaatggcactggaacccccaagcccgaggaatcggcgtgacggtcgcaaaccatccggcccggtacaaatcggcgcggcgctgggtgatgacctggtggagaagttgaaggccgcgcaggccgcccagcggcaacgcatcgaggcagaagcacgccccggtgaatcgtggcaagcggccgctgatcgaatccgcaaagaatcccggcaaccgccggcagccggtgcgccgtcgattaggaagccgcccaagggcgacgagcaaccagattttttcgttccgatgctctatgacgtgggcacccgcgatagtcgcagcatcatggacgtggccgttttccgtctgtcgaagcgtgaccgacgagctggcgaggtgatccgctacgagcttccagacgggcacgtagaggtttccgcagggccggccggcatggccagtgtgtgggattacgacctggtactgatggcggtttcccatctaaccgaatccatgaaccgataccgggaagggaagggagacaagcccggccgcgtgttccgtccacacgttgcggacgtactcaagttctgccggcgagccgatggcggaaagcagaaagacgacctggtagaaacctgcattcggttaaacaccacgcacgttgccatgcagcgtacgaagaaggccaagaacggccgcctggtgacggtatccgagggtgaagccttgattagccgctacaagatcgtaaagagcgaaaccgggcggccggagtacatcgagatcgagctagctgattggatgtaccgcgagatcacagaaggcaagaacccggacgtgctgacggttcaccccgattactttttgatcgatcccggcatcggccgttttctctaccgcctggcacgccgcgccgcaggcaaggcagaagccagatggttgttcaagacgatctacgaacgcagtggcagcgccggagagttcaagaagttctgtttcaccgtgcgcaagctgatcgggtcaaatgacctgccggagtacgatttgaaggaggaggcggggcaggctggcccgatcctagtcatgcgctaccgcaacctgatcgagggcgaagcatccgccggttcctaatgtacggagcagatgctagggcaaattgccctagcaggggaaaaaggtcgaaaaggactctttcctgtggatagcacgtacattgggaacccaaagccgtacattgggaaccggaacccgtacattgggaacccaaagccgtacattgggaaccggtcacacatgtaagtgactgatataaaagagaaaaaaggcgatttttccgcctaaaactctttaaaacttattaaaactcttaaaacccgcctggcctgtgcataactgtctggccagcgcacagccgaagagctgcaaaaagcgcctacccttcggtcgctgcgctccctacgccccgccgcttcgcgtcggcctatcgcggccgctggccgctcaaaaatggctggcctacggccaggcaatctaccagggcgcggacaagccgcgccgtcgccactcgaccgccggcgcccacatcaaggcaccctgcctcgcgcgtttcggtgatgacggtgaaaacctctgacacatgcagctcccggtgacggtcacagcttgtctgtaagcggatgccgggagcagacaagcccgtcagggcgcgtcagcgggtgttggcgggtgtcggggcgcagccatgacccagtcacgtagcgatagcggagtgtatactggcttaactatgcggcatcagagcagattgtactgagagtgcaccatatgcggtgtgaaataccgcacagatgcgtaaggagaaaataccgcatcaggcgctcttccgcttcctcgctcactgactcgctgcgctcggtcgttcggctgcggcgagcggtatcagctcactcaaaggcggtaatacggttatccacagaatcaggggataacgcaggaaagaacatgtgagcaaaaggccagcaaaaggccaggaaccgtaaaaaggccgcgttgctggcgtttttccataggctccgcccccctgacgagcatcacaaaaatcgacgctcaagtcagaggtggcgaaacccgacaggactataaagataccaggcgtttccccctggaagctccctcgtgcgctctcctgttccgaccctgccgcttaccggatacctgtccgcctttctcccttcgggaagcgtggcgctttctcatagctcacgctgtaggtatctcagttcggtgtaggtcgttcgctccaagctgggctgtgtgcacgaaccccccgttcagcccgaccgctgcgccttatccggtaactatcgtcttgagtccaacccggtaagacacgacttatcgccactggcagcagccactggtaacaggattagcagagcgaggtatgtaggcggtgctacagagttcttgaagtggtggcctaactacggctacactagaaggacagtatttggtatctgcgctctgctgaagccagttaccttcggaaaaagagttggtagctcttgatccggcaaacaaaccaccgctggtagcggtggtttttttgtttgcaagcagcagattacgcgcagaaaaaaaggatctcaagaagatcctttgatcttttctacggggtctgacgctcagtggaacgaaaactcacgttaagggattttggtcatgcattctaggtgattatttgccgactaccttggtgatctcgcctttcacgtagtggacaaattcttccaactgatctgcgcgcgaggccaagcgatcttcttcttgtccaagataagcctgtctagcttcaagtatgacgggctgatactgggccggcaggcgctccattgcccagtcggcagcgacatccttcggcgcgattttgccggttactgcgctgtaccaaatgcgggacaacgtaagcactacatttcgctcatcaccagcccagtcgggcggcgagttccatagcgttaaggtttcatttagcgcctcaaatagatcctgttcaggaaccggatcaaagagttcctccgccgctggacctaccaaggcaacgctatgttctcttgcttttgtcagcaagatagccagatcaatgtcgatcgtggctggctcgaagatacctgcaagaatgtcattgcgctgccattctccaaattgcagttcgcgcttagctggataacgccacggaatgatgtcgtcgtgcacaacaatggtgacttctacagcgcggagaatctcgctctctccaggggaagccgaagtttccaaaaggtcgttgatcaaagctcgccgcgttgtttcatcaagccttacggtcaccgtaaccagcaaatcaatatcactgtgtggcttcaggccgccatccactgcggagccgtacaaatgtacggccagcaacgtcggttcgagatggcgctcgatgacgccaactacctctgatagttgagtcgatacttcggcgatcaccgcttccctcataatgtttaactttgttttagggcgactgccctgctgcgtaacatcgttgctgctccataacatcaaacatcgacccacggcgtaacgcgcttgctgcttggatgcccgaggcatagactgtaccccaaaaaaacagtcataacaagccatgaaaaccgccactgcgccgttaccaccgctgcgttcggtcaaggttctggaccagttgcgtgagcgcatacgctacttgcattacagcttacgaaccgaacaggcttatgtccactgggttcgtgccttcatccgtttccacggtgtgcgtcacccggcaaccttgggtagcagcgaagtcgaggcatttctgtcctggctggaacagaacttattatttccttcctcttttctacagtatttaaagataccccaagaagctaattataacaagacgaactccaattcactgttccttgcattctaaaaccttaaataccagaaaacagctttttcaaagttgttttcaaagttggcgtataacatagtatcgacggagccgattttgaaaccgcggtgatcacaggcagcaacgctctgtcatcgttacaatcaacatgctaccctccgcgagatcatccgtgtttcaaacccggcagcttagttgccgttcttccgaatagcatcggtaacatgagcaaagtctgccgccttacaacggctctcccgctgacgccgtcccggactgatgggctgcctgtatcgagtggtgattttgtgccgagctgccggtcggggagctgttggctggctggtggcaggatatattgtggtgtaaacataacggatccggtctcaggagggaggtcaacatggtggagcacgacactctggtctactccaaaaatgtcaaagatacagtctcagaagatcaaagggctattgagacttttcaacaaaggataatttcgggaaacctcctcggattccattgcccagctatctgtcacttcatcgaaaggacagtagaaaaggaaggtggctcctacaaatgccatcattgcgataaaggaaaggctatcattcaagatctctctgccgacagtggtcccaaagatggacccccacccacgaggagcatcgtggaaaaagaagaggttccaaccacgtctacaaagcaagtggattgatgtgacatctccactgacgtaagggatgacgcacaatcccactatccttcgcaagacccttcctctatataaggaagttcatttcatttggagaggacacgctcgagtataagagctcatttttacaacaattaccaacaacaacaaacaacaaacaacattacaattacatttacaattatcgatacaatggaacgagctatacaaggaaacgacgctagggaacaagctaacagtgaacgttgggatggaggatcaggaggtaccacttctcccttcaaacttcctgacgaaagtccgagttggactgagtggcggctacataacgatgagactaattcgaatcaagataatccccttggtttcaaggaaagctggggtttcgggaaagttgtatttaagagatatctcagatacgacaggacggaagcttcactgcacagagtccttggatcttggacgggagattcggttaactatgcagcatctcgatttttcggtttcgaccagatcggatgtacctatagtattcggtttcgaggagttagtatcaccgtttctggaggctctcgaactcttcagcatctctgtgagatggcaattcggtctaagcaagaactgctacagcttgccccaatcgaagtggaaagtaatgtatcaagaggatgccctgaaggtactgaaaccttcgaaaaagaaagcgagtgagcttgtcaagcagatcgttcaaacatttggcaataaagtttcttaagattgaatcctgttgccggtcttgcgatgattatcatataatttctgttgaattacgttaagcatgtaataattaacatgtaatgcatgacgttatttatgagatgggtttttatgattagagtcccgcaattatacatttaatacgcgatagaaaacaaaatatagcgcgcaaactaggataaattatcgcgcgcggtgtcatctatgttactagatcgacgctccatggaggtgactccgaggggttgcctcaaactctatcttataaccggcgtggaggcatggaggcaggggtattttggtcattttaatagatagtggaaaatgacgtggaatttacttaaagacgaagtctttgcgacaagggggggcccacgccgaatttaatattaccggcgtggcccccccttatcgcgagtgctttagcacgagcggtccagatttaaagtagaaaatttcccgcccactagggttaaaggtgttcacactataaaagcatatacgatgtgatggtatttgctcgctgcttggagggaaacctcctcggattccattgcccagctatctgtcactttattgagaagatagtggaaaaggaaggtggctcctacaaatgccatcattgcgataaaggaaaggccatcgttgaagatgcctctgccgacagtggtcccaaagatggacccccacccacgaggagcatcgtggaaaaagaagacgttccaaccacgtcttcaaagcaagtggattgatgtgatatctccactgacgtaagggatgacgcacaatcccactatccttcgcaagacccttcctctatataaggaagttcatttcatttggagaggtattaaaatcttaataggttttgataaaagcgaacgtggggaaacccgaaccaaaccttcttctaaactctctctcatctctcttaaagcaaacttctctcttgtctttcttgcgtgagcgatcttcaacgttgtcagatcgtgcttcggcaccagtacaacgttttctttcactgaagcgaaatcaaagatctctttgtggacacgtagtgcggcgccattaaataacgtgtacttgtcctattcttgtcggtgtggtcttgggaaaagaaagcttgctggaggctgctgttcagccccatacattacttgttacgattctgctgactttcggcgggtgcaatatctctacttctgcttgacgaggtattgttgcctgtacttctttcttcttcttcttgctgattggttctataagaaatctagtattttctttgaaacagagttttcccgtggttttcgaacttggagaaagattgttaagcttctgtatattctgcccaaattcgcgaccggtaatgaatggtgagcaagggcgaggagctgttcaccggggtggtgcccatcctggtcgagctggacggcgacgtaaacggccacaagttcagcgtgtccggcgagggcgagggcgatgccacctacggcaagctgaccctgaagttcatctgcaccaccggcaagctgcccgtgccctggcccaccctcgtgaccaccttcagctacggcgtgcagtgcttcagccgctaccccgaccacatgaagcagcacgacttcttcaagtccgccatgcccgaaggctacgtccaggagcgcaccatcttcttcaaggacgacggcaactacaagacccgcgccgaggtgaagttcgagggcgacaccctggtgaaccgcatcgagctgaagggcatcgacttcaaggaggacggcaacatcctggggcacaagctggagtacaactacaacagccacaacgtctatatcatggccgacaagcagaagaacggcatcaaggtgaacttcaagatccgccacaacatcgaggacggcagcgtgcagctcgccgaccactaccagcagaacacccccatcggcgacggccccgtgctgctgcccgacaaccactacctgagcacccagtccgccctgagcaaagaccccaacgagaagcgcgatcacatggtcctgctggagttcgtgaccgccgccgggatcactcacggcatggacgagctgtacaagtaatgagctttcccgggcatcaccatcaccatcactagctcgaggcctttaactctggtttcattaaattttctttagtttgaatttactgttattcggtgtgcatttctatgtttggtgagcggttttctgtgctcagagtgtgtttattttatgtaatttaatttctttgtgagctcctgtttagcaggtcgtcccttcagcaaggacacaaaaagattttaattttattaaaaaaaaaaaaaaaaaagaccgggaattcgatatcaagcttatcgacctgcagatcgttcaaacatttggcaataaagtttcttaagattgaatcctgttgccggtcttgcggtgattatcatataatttctgttgaattacgttaagcatgtaataattaacatgtaatgcatgacgttatttatgagatgggtttttatgattagagtcccgcaattatacatttaatacgcgatagaaaacaaaatatagcgcgcaaactaggataaattatcgcgcgcggtgtcatctatgttactagatccgctggtaggagggaaacctcctcggattccattgcccagctatctgtcactttattgagaagatagtggaaaaggaaggtggctcctacaaatgccatcattgcgataaaggaaaggccatcgttgaagatgcctctgccgacagtggtcccaaagatggacccccacccacgaggagcatcgtggaaaaagaagacgttccaaccacgtcttcaaagcaagtggattgatgtgatatctccactgacgtaagggatgacgcacaatcccactatccttcgcaagacccttcctctatataaggaagttcatttcatttggagaggtattaaaatcttaataggttttgataaaagcgaacgtggggaaacccgaaccaaaccttcttctaaactctctctcatctctcttaaagcaaacttctctcttgtctttcttgcgtgagcgatcttcaacgttgtcagatcgtgcttcggcaccagtacaacgttttctttcactgaagcgaaatcaaagatctctttgtggacacgtagtgcggcgccattaaataacgtgtacttgtcctattcttgtcggtgtggtcttgggaaaagaaagcttgctggaggctgctgttcagccccatacattacttgttacgattctgctgactttcggcgggtgcaatatctctacttctgcttgacgaggtattgttgcctgtacttctttcttcttcttcttgctgattggttctataagaaatctagtattttctttgaaacagagttttcccgtggttttcgaacttggagaaagattgttaagcttctgtatattctgcccaaattcgcgaccggtaatggggtcatccaagaatgttatcaaggagttcatgaggtttaaggttcgcatggaaggaacggtcaatgggcacgagtttgaaatagaaggcgaaggagaggggaggccatacgaaggccacaataccgtaaagcttaaggtaaccaaggggggacctttgccatttgcttgggatattttgtcaccacaatttcagtatggaagcaaggtatatgtcaagcaccctgccgacataccagactataaaaagctgtcatttcctgaaggatttaaatgggaaagggtcatgaactttgaagatggtggcgtcgttactgtaacccaggattccagtttgcaggatggctgtttcatctacaaggtcaagttcattggcgtgaactttccttccgatggacctgttatgcaaaagaaaacaatgggctgggaagccagcactgagcgtttgtatcctcgtgatggcgtgttgaaaggagagattcataaggctctgaagctgaaagacggtggtcattacctagttgaattcaaaagtatttacatggcaaagaagcctgtgcagctaccagggtactactatgttgactccaaactggatataacaagccacaacgaagattatacaatcgttgagcagtatgaaagaaccgagggacgccaccatctgttcctttaagctttcccgggcatcaccatcaccatcactagctcgaggcctttaactctggtttcattaaattttctttagtttgaatttactgttattcggtgtgcatttctatgtttggtgagcggttttctgtgctcagagtgtgtttattttatgtaatttaatttctttgtgagctcctgtttagcaggtcgtcccttcagcaaggacacaaaaagattttaattttattaaaaaaaaaaaaaaaaaagaccgggaattcgatatcaagcttatcgacctgcagatcgttcaaacatttggcaataaagtttcttaagattgaatcctgttgccggtcttgcggtgattatcatataatttctgttgaattacgttaagcatgtaataattaacatgtaatgcatgacgttatttatgagatgggtttttatgattagagtcccgcaattatacatttaatacgcgatagaaaacaaaatatagcgcgcaaactaggataaattatcgcgcgcggtgtcatctatgttactagatccgctgagaaactctagtcaataccatcacatcgtatatgcttgtatagtgtgaacacctttaaccctagtgggcgggaaattttctactttaaatctggaccgctcgtgctaaagcactcgcgataagggggggccacgccggtaatattaaattcggcgtgggcccccccttgtcgcaaagacttcgtctttaagtaaattccacgtcattttccactatctattaaaatgaccaaaatacccctgcctccatgcctccacgccggttataagatagagtttgaggcaacccctcggagtcacaacaaaatgtcattgtttgcctccctgctgcggtttttcaccgaagttcatgccagtccagcgtttttgcagcagaaaagccgccgacttcggtttgcggtcgcgagtgaagatccctttcttgttaccgccaacgcgcaatatgccttgcgaggtcgcaaaatcggcgaaattccatacctgttcaccgacgacggcgctgacgcgatcaaagacgcggtgatacatatccagccatgcacactgatactcttcactccacatgtcggtgtacattgagtgcagcccggctaacgtatccacgccgtattcggtgatgataatcggctgatgcagtttctcctgccaggccagaagttctttttccagtaccttctctgccgtttccaaatcgccgctttggacataccatccgtaataacggttcaggcacagcacatcaaagagatcgctgatggtatcggtgtgagcgtcgcagaacattacattgacgcaggtgatcggacgcgtcgggtcgagtttacgcgttgcttccgccagtggcgcgaaatattcccgtgcaccttgcggacgggtatccggttcgttggcaatactccacatcaccacgcttgggtggtttttgtcacgcgctatcagctctttaatcgcctgtaagtgcgcttgctgagtttccccgttgactgcctcttcgctgtacagttctttcggcttgttgcccgcttcgaaaccaatgcctaaagagaggttaaagccgacagcagcagtttcatcaatcaccacgatgccatgttcatctgcccagtcgagcatctcttcagcgtaagggtaatgcgaggtacggtaggagttggccccaatccagtccattaatgcgtggtcgtgcaccatcagcacgttatcgaatcctttgccacgcaagtccgcatcttcatgacgaccaaagccagtaaagtagaacggtttgtggttaatcaggaactgttcgcccttcactgccactgaccggatgccgacgcgaagcgggtagatatcacactctgtctggcttttggctgtgacgcacagttcatagagataaccttcacccggttgccagaggtgcggattcaccacttgcaaagtcccgctagtgccttgtccagttgcaaccacctgttgatccgcatcacgcagttcaacgctgacatcaccattggccaccacctgtaaggggaaaaaaattgacaaaaagcaaaggtcaagcaaatgtatggaaatataaaatttcacacagtatctcttaagttacacgttgaagcatgaagtacctgccagtcaacagacgcgtggttacagtcttgcgcgacatgcgtcaccacggtgatatcgtccacccaggtgttcggcgtggtgtagagcattacgctgcgatggattccggcatagttaaagaaatcatggaagtaagactgctttttcttgccgttttcgtcggtaatcaccattcccggcgggatagtctgccagttcagttcgttgttcacacaaacggtgatacgtacacttttcccggcaataacatacggcgtgacatcggcttcaaatggcgtatagccgccctgatgctccatcacttcctgattattgacccacactttgccgtaatgagtgaccgcatcgaaacgcagcacgatacgctggcctgcccaacctgaaaaaagattattagcgtcagtacaaatagtatacttcaaaaaattaaaaatacagaccaaaaaagagagagtaagacttacctttcggtataaagacttcgcgctgataccagacgttgcccgcataattacgaatatctgcatcggcgaactgatcgttaaaactgcctggcacagcaattgcccggctttcttgtaacgcgctttcccaccaacgctgatcaattccacagttttcgcgatccagactgaatgcccacaggccgtcgagttttttgatttcacgggttggggtttctacaggacgtaacataagggactgacccattgcttaacgtgcctctcctcatacgagtttatctaaagtgattattttttttggggtgtttgtttttggattgtctttttttgtttatttcgtgtgttatgtaacatatgtaatttctatctacttgcacaatgaaatatattcataaaataatcattcgcttgac

**pGB-R-DsRed**

cgctgtcatgagaccggatcctgacaggatatattggcgggtaaacctaagagaaaagagcgtttattagaataatcggatatttaaaagggcgtgaaaaggtttatccgttcgtccatttgtatgtgcatgccaaccacagggttcccctcgggatcaaagtactttgatccaacccctccgctgctatagtgcagtcggcttctgacgttcagtgcagccgtcatctgaaaacgacatgtcgcacaagtcctaagttacgcgacaggctgccgccctgcccttttcctggcgttttcttgtcgcgtgttttagtcgcataaagtagaatacttgcgactagaaccggagacattacgccatgaacaagagcgccgccgctggcctgctgggctatgcccgcgtcagcaccgacgaccaggacttgaccaaccaacgggccgaactgcacgcggccggctgcaccaagctgttttccgagaagatcaccggcaccaggcgcgaccgcccggagctggccaggatgcttgaccacctacgccctggcgacgttgtgacagtgaccaggctagaccgcctggcccgcagcacccgcgacctactggacattgccgagcgcatccaggaggccggcgcgggcctgcgtagcctggcagagccgtgggccgacaccaccacgccggccggccgcatggtgttgaccgtgttcgccggcattgccgagttcgagcgttccctaatcatcgaccgcacccggagcgggcgcgaggccgccaaggcccgaggcgtgaagtttggcccccgccctaccctcaccccggcacagatcgcgcacgcccgcgagctgatcgaccaggaaggccgcaccgtgaaagaggcggctgcactgcttggcgtgcatcgctcgaccctgtaccgcgcacttgagcgcagcgaggaagtgacgcccaccgaggccaggcggcgcggtgccttccgtgaggacgcattgaccgaggccgacgccctggcggccgccgagaatgaacgccaagaggaacaagcatgaaaccgcaccaggacggccaggacgaaccgtttttcattaccgaagagatcgaggcggagatgatcgcggccgggtacgtgttcgagccgcccgcgcacctctcaaccgtgcggctgcatgaaatcctggccggtttgtctgatgccaagctggcggcctggccggccagcttggccgctgaagaaaccgagcgccgccgtctaaaaaggtgatgtgtatttgagtaaaacagcttgcgtcatgcggtcgctgcgtatatgatccgatgagtaaataaacaaatacgcaaggggaacgcatgaaggttatcgctgtacttaaccagaaaggcgggtcaggcaagacgaccatcggaacccatctagcccgcgccctgcaactcgccggggccgatgttctgttagtcgattccgatccccagggcagtgcccgcgattgggcggccgtgcgggaagatcaaccgctaaccgttgtcggcatcgaccgcccgacgattgaccgcgacgtgaaggccatcggccggcgcgacttcgtagtgatcgacggagcgccccaggcggcggacttggctgtgtccgcgatcaaggcagccgacttcgtgctgattccggtgcagccaagcccttacgacatatgggccaccgccgacctggtggagctggttaagcagcgcattgaggtcacggatggaaggctacaagcggcctttgtcgtgtcgcgggcgatcaaaggcacgcgcatcggcggtgaggttgccgaggcgctggccgggtacgagctgcccattcttgagtcccgtatcacgcagcgcgtgagctacccaggcactgccgccgccggcacaaccgttcttgaatcagaacccgagggcgacgctgcccgcgaggtccaggcgctggccgctgaaattaaatcaaaactcatttgagttaatgaggtaaagagaaaatgagcaaaagcacaaacacgctaagtgccggccgtccgagcgcacgcagcagcaaggctgcaacgttggccagcctggcagacacgccagccatgaagcgggtcaactttcagttgccggcggaggatcacaccaagctgaagatgtacgcggtacgccaaggcaagaccattaccgagctgctatctgaatagatcgcgcagctaccagagtaaatgagcaaatgaataaatgagtagatgaattttagcggctaaaggaggcggcatggaaaatcaagaacaaccaggcaccgacgccgtggaatgccccatgtgtggaggaacgggcggttggccaggcgtaagcggctgggttgtctgccggccctgcaatggcactggaacccccaagcccgaggaatcggcgtgacggtcgcaaaccatccggcccggtacaaatcggcgcggcgctgggtgatgacctggtggagaagttgaaggccgcgcaggccgcccagcggcaacgcatcgaggcagaagcacgccccggtgaatcgtggcaagcggccgctgatcgaatccgcaaagaatcccggcaaccgccggcagccggtgcgccgtcgattaggaagccgcccaagggcgacgagcaaccagattttttcgttccgatgctctatgacgtgggcacccgcgatagtcgcagcatcatggacgtggccgttttccgtctgtcgaagcgtgaccgacgagctggcgaggtgatccgctacgagcttccagacgggcacgtagaggtttccgcagggccggccggcatggccagtgtgtgggattacgacctggtactgatggcggtttcccatctaaccgaatccatgaaccgataccgggaagggaagggagacaagcccggccgcgtgttccgtccacacgttgcggacgtactcaagttctgccggcgagccgatggcggaaagcagaaagacgacctggtagaaacctgcattcggttaaacaccacgcacgttgccatgcagcgtacgaagaaggccaagaacggccgcctggtgacggtatccgagggtgaagccttgattagccgctacaagatcgtaaagagcgaaaccgggcggccggagtacatcgagatcgagctagctgattggatgtaccgcgagatcacagaaggcaagaacccggacgtgctgacggttcaccccgattactttttgatcgatcccggcatcggccgttttctctaccgcctggcacgccgcgccgcaggcaaggcagaagccagatggttgttcaagacgatctacgaacgcagtggcagcgccggagagttcaagaagttctgtttcaccgtgcgcaagctgatcgggtcaaatgacctgccggagtacgatttgaaggaggaggcggggcaggctggcccgatcctagtcatgcgctaccgcaacctgatcgagggcgaagcatccgccggttcctaatgtacggagcagatgctagggcaaattgccctagcaggggaaaaaggtcgaaaaggactctttcctgtggatagcacgtacattgggaacccaaagccgtacattgggaaccggaacccgtacattgggaacccaaagccgtacattgggaaccggtcacacatgtaagtgactgatataaaagagaaaaaaggcgatttttccgcctaaaactctttaaaacttattaaaactcttaaaacccgcctggcctgtgcataactgtctggccagcgcacagccgaagagctgcaaaaagcgcctacccttcggtcgctgcgctccctacgccccgccgcttcgcgtcggcctatcgcggccgctggccgctcaaaaatggctggcctacggccaggcaatctaccagggcgcggacaagccgcgccgtcgccactcgaccgccggcgcccacatcaaggcaccctgcctcgcgcgtttcggtgatgacggtgaaaacctctgacacatgcagctcccggtgacggtcacagcttgtctgtaagcggatgccgggagcagacaagcccgtcagggcgcgtcagcgggtgttggcgggtgtcggggcgcagccatgacccagtcacgtagcgatagcggagtgtatactggcttaactatgcggcatcagagcagattgtactgagagtgcaccatatgcggtgtgaaataccgcacagatgcgtaaggagaaaataccgcatcaggcgctcttccgcttcctcgctcactgactcgctgcgctcggtcgttcggctgcggcgagcggtatcagctcactcaaaggcggtaatacggttatccacagaatcaggggataacgcaggaaagaacatgtgagcaaaaggccagcaaaaggccaggaaccgtaaaaaggccgcgttgctggcgtttttccataggctccgcccccctgacgagcatcacaaaaatcgacgctcaagtcagaggtggcgaaacccgacaggactataaagataccaggcgtttccccctggaagctccctcgtgcgctctcctgttccgaccctgccgcttaccggatacctgtccgcctttctcccttcgggaagcgtggcgctttctcatagctcacgctgtaggtatctcagttcggtgtaggtcgttcgctccaagctgggctgtgtgcacgaaccccccgttcagcccgaccgctgcgccttatccggtaactatcgtcttgagtccaacccggtaagacacgacttatcgccactggcagcagccactggtaacaggattagcagagcgaggtatgtaggcggtgctacagagttcttgaagtggtggcctaactacggctacactagaaggacagtatttggtatctgcgctctgctgaagccagttaccttcggaaaaagagttggtagctcttgatccggcaaacaaaccaccgctggtagcggtggtttttttgtttgcaagcagcagattacgcgcagaaaaaaaggatctcaagaagatcctttgatcttttctacggggtctgacgctcagtggaacgaaaactcacgttaagggattttggtcatgcattctaggtgattatttgccgactaccttggtgatctcgcctttcacgtagtggacaaattcttccaactgatctgcgcgcgaggccaagcgatcttcttcttgtccaagataagcctgtctagcttcaagtatgacgggctgatactgggccggcaggcgctccattgcccagtcggcagcgacatccttcggcgcgattttgccggttactgcgctgtaccaaatgcgggacaacgtaagcactacatttcgctcatcaccagcccagtcgggcggcgagttccatagcgttaaggtttcatttagcgcctcaaatagatcctgttcaggaaccggatcaaagagttcctccgccgctggacctaccaaggcaacgctatgttctcttgcttttgtcagcaagatagccagatcaatgtcgatcgtggctggctcgaagatacctgcaagaatgtcattgcgctgccattctccaaattgcagttcgcgcttagctggataacgccacggaatgatgtcgtcgtgcacaacaatggtgacttctacagcgcggagaatctcgctctctccaggggaagccgaagtttccaaaaggtcgttgatcaaagctcgccgcgttgtttcatcaagccttacggtcaccgtaaccagcaaatcaatatcactgtgtggcttcaggccgccatccactgcggagccgtacaaatgtacggccagcaacgtcggttcgagatggcgctcgatgacgccaactacctctgatagttgagtcgatacttcggcgatcaccgcttccctcataatgtttaactttgttttagggcgactgccctgctgcgtaacatcgttgctgctccataacatcaaacatcgacccacggcgtaacgcgcttgctgcttggatgcccgaggcatagactgtaccccaaaaaaacagtcataacaagccatgaaaaccgccactgcgccgttaccaccgctgcgttcggtcaaggttctggaccagttgcgtgagcgcatacgctacttgcattacagcttacgaaccgaacaggcttatgtccactgggttcgtgccttcatccgtttccacggtgtgcgtcacccggcaaccttgggtagcagcgaagtcgaggcatttctgtcctggctggaacagaacttattatttccttcctcttttctacagtatttaaagataccccaagaagctaattataacaagacgaactccaattcactgttccttgcattctaaaaccttaaataccagaaaacagctttttcaaagttgttttcaaagttggcgtataacatagtatcgacggagccgattttgaaaccgcggtgatcacaggcagcaacgctctgtcatcgttacaatcaacatgctaccctccgcgagatcatccgtgtttcaaacccggcagcttagttgccgttcttccgaatagcatcggtaacatgagcaaagtctgccgccttacaacggctctcccgctgacgccgtcccggactgatgggctgcctgtatcgagtggtgattttgtgccgagctgccggtcggggagctgttggctggctggtggcaggatatattgtggtgtaaacataacggatccggtctcaggagggagtcgattaaaaatcccaattatatttggtctaatttagtttggtattgagtaaaacaaattcgaaccaaaccaaaatataaatatatagtttttatatatatgcctttaagactttttatagaattttctttaaaaaatatctagaaatatttgcgactcttctggcatgtaatatttcgttaaatatgaagtgctccatttttattaactttaaataattggttgtacgatcactttcttatcaagtgttactaaaatgcgtcaatctctttgttcttccatattcatatgtcaaaatctatcaaaattcttatatatctttttcgaatttgaagtgaaatttcgataatttaaaattaaatagaacatatcattatttaggtatcatattgatttttatacttaattactaaatttggttaactttgaaagtgtacatcaacgaaaaattagtcaaacgactaaaataaataaatatcatgtgttattaagaaaattctcctataagaatattttaatagatcatatgtttgtaaaaaaaattaatttttactaacacatatatttacttatcaaaaatttgacaaagtaagattaaaataatattcatctaacaaaaaaaaaaccagaaaatgctgaaaacccggcaaaaccgaaccaatccaaaccgatatagttggtttggtttgattttgatataaaccgaaccaactcggtccatttgcacccctaatcataatagctttaatatttcaagatattattaagttaacgttgtcaatatcctggaaattttgcaaaatgaatcaagcctatatggctgtaatatgaatttaaaagcagctcgatgtggtggtaatatgtaatttacttgattctaaaaaaatatcccaagtattaataatttctgctaggaagaaggttagctacgatttacagcaaagccagaatacaaagaaccataaagtgattgaagctcgaaatatacgaaggaacaaatatttttaaaaaaatacgcaatgacttggaacaaaagaaagtgatatattttttgttcttaaacaagcatcccctctaaagaatggcagttttcctttgcatgtaactattatgctcccttcgttacaaaaattttggactactattgggaacttcttctgaaaatagtcgctccatggagccctcaattgtactaccatcatttcttgttccgctgcttggaggtgactccgaggggttgcctcaaactctatcttataaccggcgtggaggcatggaggcaggggtattttggtcattttaatagatagtggaaaatgacgtggaatttacttaaagacgaagtctttgcgacaagggggggcccacgccgaatttaatattaccggcgtggcccccccttatcgcgagtgctttagcacgagcggtccagatttaaagtagaaaatttcccgcccactagggttaaaggtgttcacactataaaagcatatacgatgtgatggtatttgctcgctggtaggagggaaacctcctcggattccattgcccagctatctgtcactttattgagaagatagtggaaaaggaaggtggctcctacaaatgccatcattgcgataaaggaaaggccatcgttgaagatgcctctgccgacagtggtcccaaagatggacccccacccacgaggagcatcgtggaaaaagaagacgttccaaccacgtcttcaaagcaagtggattgatgtgatatctccactgacgtaagggatgacgcacaatcccactatccttcgcaagacccttcctctatataaggaagttcatttcatttggagaggtattaaaatcttaataggttttgataaaagcgaacgtggggaaacccgaaccaaaccttcttctaaactctctctcatctctcttaaagcaaacttctctcttgtctttcttgcgtgagcgatcttcaacgttgtcagatcgtgcttcggcaccagtacaacgttttctttcactgaagcgaaatcaaagatctctttgtggacacgtagtgcggcgccattaaataacgtgtacttgtcctattcttgtcggtgtggtcttgggaaaagaaagcttgctggaggctgctgttcagccccatacattacttgttacgattctgctgactttcggcgggtgcaatatctctacttctgcttgacgaggtattgttgcctgtacttctttcttcttcttcttgctgattggttctataagaaatctagtattttctttgaaacagagttttcccgtggttttcgaacttggagaaagattgttaagcttctgtatattctgcccaaattcgcgaccggtaatggggtcatccaagaatgttatcaaggagttcatgaggtttaaggttcgcatggaaggaacggtcaatgggcacgagtttgaaatagaaggcgaaggagaggggaggccatacgaaggccacaataccgtaaagcttaaggtaaccaaggggggacctttgccatttgcttgggatattttgtcaccacaatttcagtatggaagcaaggtatatgtcaagcaccctgccgacataccagactataaaaagctgtcatttcctgaaggatttaaatgggaaagggtcatgaactttgaagatggtggcgtcgttactgtaacccaggattccagtttgcaggatggctgtttcatctacaaggtcaagttcattggcgtgaactttccttccgatggacctgttatgcaaaagaaaacaatgggctgggaagccagcactgagcgtttgtatcctcgtgatggcgtgttgaaaggagagattcataaggctctgaagctgaaagacggtggtcattacctagttgaattcaaaagtatttacatggcaaagaagcctgtgcagctaccagggtactactatgttgactccaaactggatataacaagccacaacgaagattatacaatcgttgagcagtatgaaagaaccgagggacgccaccatctgttcctttaagctttcccgggcatcaccatcaccatcactagctcgaggcctttaactctggtttcattaaattttctttagtttgaatttactgttattcggtgtgcatttctatgtttggtgagcggttttctgtgctcagagtgtgtttattttatgtaatttaatttctttgtgagctcctgtttagcaggtcgtcccttcagcaaggacacaaaaagattttaattttattaaaaaaaaaaaaaaaaaagaccgggaattcgatatcaagcttatcgacctgcagatcgttcaaacatttggcaataaagtttcttaagattgaatcctgttgccggtcttgcggtgattatcatataatttctgttgaattacgttaagcatgtaataattaacatgtaatgcatgacgttatttatgagatgggtttttatgattagagtcccgcaattatacatttaatacgcgatagaaaacaaaatatagcgcgcaaactaggataaattatcgcgcgcggtgtcatctatgttactagatccgctgtcaaaactctagtcaataccatcacatcgtatatgcttgtatagtgtgaacacctttaaccctagtgggcgggaaattttctactttaaatctggaccgctcgtgctaaagcactcgcgataagggggggccacgccggtaatattaaattcggcgtgggcccccccttgtcgcaaagacttcgtctttaagtaaattccacgtcattttccactatctattaaaatgaccaaaatacccctgcctccatgcctccacgccggttataagatagagtttgaggcaacccctcggagtcacaacaaatgccttctgctagtaagaacttcagactccaatctaaatatgttttccttacctatcccaagtgctcatctcaaagagatgatttattccagtttctctgggagaaactcacaccttttcttattttcttccttggtgttgcttctgagcttcatcaagatggcactacccactatcatgctcttctccagcttgataaaaaaccttgtattagggatccttcttttttcgattttgaaggaaatcaccctaatatccagccagctagaaactctaaacaagtccttgattacatatcaaaggacggagatattaaaaccagaggagatttccgagatcataaggtatctcctcgcaaatctgacgcacgatggagaactattatccagactgcaacgtctaaggaggaatatcttgacatgatcaaggaagaattccctcatgaatgggcaacaaaacttcaatggctggaatattcagccaacaaattattccctccacaacctgaaccgtatgtgtcgcccttcacagaatcagatcttcgctgccacgaagatctacactcctggagggaaacccatctataccatgtaagcatagacgcttatacttacatacatcctgtctcataccaacaagctcaatctgaccttgaatggatggccgatttaaccaggacaatggaaggaatggaatccgacaccccagcctctacatctgcggaccaactcgtaccggaaagaccacctgggctagaagtctcggacgacacaactattggaacggtaccatcgatttcaccaactacgatgaacacgccacctataatatcatcgacgacatccccttcaagttcgtcccattgtggaagcaattaataggttgccagtctgatttcactgtcaaccctaaatatggaaaaaagaagaaaataaaaggtgggatcccttctataattctttgcaatcctgacgaagactggatgttatcaatgacaagtcaacagaaggattactttaaagataattgcgtcacccactatatgtgtgacggggagactttttttgctcgggaatcgtcgagtcactgaaacgtgcctctcctcatacgagtttatctaaagtgattattttttttggggtgtttgtttttggattgtctttttttgtttatttcgtgtgttatgtaacatatgtaatttctatctacttgcacaatgaaatatattcataaaataatcatt

**pGB-R-GFP**

cgctgtcatgagaccggatcctgacaggatatattggcgggtaaacctaagagaaaagagcgtttattagaataatcggatatttaaaagggcgtgaaaaggtttatccgttcgtccatttgtatgtgcatgccaaccacagggttcccctcgggatcaaagtactttgatccaacccctccgctgctatagtgcagtcggcttctgacgttcagtgcagccgtcatctgaaaacgacatgtcgcacaagtcctaagttacgcgacaggctgccgccctgcccttttcctggcgttttcttgtcgcgtgttttagtcgcataaagtagaatacttgcgactagaaccggagacattacgccatgaacaagagcgccgccgctggcctgctgggctatgcccgcgtcagcaccgacgaccaggacttgaccaaccaacgggccgaactgcacgcggccggctgcaccaagctgttttccgagaagatcaccggcaccaggcgcgaccgcccggagctggccaggatgcttgaccacctacgccctggcgacgttgtgacagtgaccaggctagaccgcctggcccgcagcacccgcgacctactggacattgccgagcgcatccaggaggccggcgcgggcctgcgtagcctggcagagccgtgggccgacaccaccacgccggccggccgcatggtgttgaccgtgttcgccggcattgccgagttcgagcgttccctaatcatcgaccgcacccggagcgggcgcgaggccgccaaggcccgaggcgtgaagtttggcccccgccctaccctcaccccggcacagatcgcgcacgcccgcgagctgatcgaccaggaaggccgcaccgtgaaagaggcggctgcactgcttggcgtgcatcgctcgaccctgtaccgcgcacttgagcgcagcgaggaagtgacgcccaccgaggccaggcggcgcggtgccttccgtgaggacgcattgaccgaggccgacgccctggcggccgccgagaatgaacgccaagaggaacaagcatgaaaccgcaccaggacggccaggacgaaccgtttttcattaccgaagagatcgaggcggagatgatcgcggccgggtacgtgttcgagccgcccgcgcacctctcaaccgtgcggctgcatgaaatcctggccggtttgtctgatgccaagctggcggcctggccggccagcttggccgctgaagaaaccgagcgccgccgtctaaaaaggtgatgtgtatttgagtaaaacagcttgcgtcatgcggtcgctgcgtatatgatccgatgagtaaataaacaaatacgcaaggggaacgcatgaaggttatcgctgtacttaaccagaaaggcgggtcaggcaagacgaccatcggaacccatctagcccgcgccctgcaactcgccggggccgatgttctgttagtcgattccgatccccagggcagtgcccgcgattgggcggccgtgcgggaagatcaaccgctaaccgttgtcggcatcgaccgcccgacgattgaccgcgacgtgaaggccatcggccggcgcgacttcgtagtgatcgacggagcgccccaggcggcggacttggctgtgtccgcgatcaaggcagccgacttcgtgctgattccggtgcagccaagcccttacgacatatgggccaccgccgacctggtggagctggttaagcagcgcattgaggtcacggatggaaggctacaagcggcctttgtcgtgtcgcgggcgatcaaaggcacgcgcatcggcggtgaggttgccgaggcgctggccgggtacgagctgcccattcttgagtcccgtatcacgcagcgcgtgagctacccaggcactgccgccgccggcacaaccgttcttgaatcagaacccgagggcgacgctgcccgcgaggtccaggcgctggccgctgaaattaaatcaaaactcatttgagttaatgaggtaaagagaaaatgagcaaaagcacaaacacgctaagtgccggccgtccgagcgcacgcagcagcaaggctgcaacgttggccagcctggcagacacgccagccatgaagcgggtcaactttcagttgccggcggaggatcacaccaagctgaagatgtacgcggtacgccaaggcaagaccattaccgagctgctatctgaatagatcgcgcagctaccagagtaaatgagcaaatgaataaatgagtagatgaattttagcggctaaaggaggcggcatggaaaatcaagaacaaccaggcaccgacgccgtggaatgccccatgtgtggaggaacgggcggttggccaggcgtaagcggctgggttgtctgccggccctgcaatggcactggaacccccaagcccgaggaatcggcgtgacggtcgcaaaccatccggcccggtacaaatcggcgcggcgctgggtgatgacctggtggagaagttgaaggccgcgcaggccgcccagcggcaacgcatcgaggcagaagcacgccccggtgaatcgtggcaagcggccgctgatcgaatccgcaaagaatcccggcaaccgccggcagccggtgcgccgtcgattaggaagccgcccaagggcgacgagcaaccagattttttcgttccgatgctctatgacgtgggcacccgcgatagtcgcagcatcatggacgtggccgttttccgtctgtcgaagcgtgaccgacgagctggcgaggtgatccgctacgagcttccagacgggcacgtagaggtttccgcagggccggccggcatggccagtgtgtgggattacgacctggtactgatggcggtttcccatctaaccgaatccatgaaccgataccgggaagggaagggagacaagcccggccgcgtgttccgtccacacgttgcggacgtactcaagttctgccggcgagccgatggcggaaagcagaaagacgacctggtagaaacctgcattcggttaaacaccacgcacgttgccatgcagcgtacgaagaaggccaagaacggccgcctggtgacggtatccgagggtgaagccttgattagccgctacaagatcgtaaagagcgaaaccgggcggccggagtacatcgagatcgagctagctgattggatgtaccgcgagatcacagaaggcaagaacccggacgtgctgacggttcaccccgattactttttgatcgatcccggcatcggccgttttctctaccgcctggcacgccgcgccgcaggcaaggcagaagccagatggttgttcaagacgatctacgaacgcagtggcagcgccggagagttcaagaagttctgtttcaccgtgcgcaagctgatcgggtcaaatgacctgccggagtacgatttgaaggaggaggcggggcaggctggcccgatcctagtcatgcgctaccgcaacctgatcgagggcgaagcatccgccggttcctaatgtacggagcagatgctagggcaaattgccctagcaggggaaaaaggtcgaaaaggactctttcctgtggatagcacgtacattgggaacccaaagccgtacattgggaaccggaacccgtacattgggaacccaaagccgtacattgggaaccggtcacacatgtaagtgactgatataaaagagaaaaaaggcgatttttccgcctaaaactctttaaaacttattaaaactcttaaaacccgcctggcctgtgcataactgtctggccagcgcacagccgaagagctgcaaaaagcgcctacccttcggtcgctgcgctccctacgccccgccgcttcgcgtcggcctatcgcggccgctggccgctcaaaaatggctggcctacggccaggcaatctaccagggcgcggacaagccgcgccgtcgccactcgaccgccggcgcccacatcaaggcaccctgcctcgcgcgtttcggtgatgacggtgaaaacctctgacacatgcagctcccggtgacggtcacagcttgtctgtaagcggatgccgggagcagacaagcccgtcagggcgcgtcagcgggtgttggcgggtgtcggggcgcagccatgacccagtcacgtagcgatagcggagtgtatactggcttaactatgcggcatcagagcagattgtactgagagtgcaccatatgcggtgtgaaataccgcacagatgcgtaaggagaaaataccgcatcaggcgctcttccgcttcctcgctcactgactcgctgcgctcggtcgttcggctgcggcgagcggtatcagctcactcaaaggcggtaatacggttatccacagaatcaggggataacgcaggaaagaacatgtgagcaaaaggccagcaaaaggccaggaaccgtaaaaaggccgcgttgctggcgtttttccataggctccgcccccctgacgagcatcacaaaaatcgacgctcaagtcagaggtggcgaaacccgacaggactataaagataccaggcgtttccccctggaagctccctcgtgcgctctcctgttccgaccctgccgcttaccggatacctgtccgcctttctcccttcgggaagcgtggcgctttctcatagctcacgctgtaggtatctcagttcggtgtaggtcgttcgctccaagctgggctgtgtgcacgaaccccccgttcagcccgaccgctgcgccttatccggtaactatcgtcttgagtccaacccggtaagacacgacttatcgccactggcagcagccactggtaacaggattagcagagcgaggtatgtaggcggtgctacagagttcttgaagtggtggcctaactacggctacactagaaggacagtatttggtatctgcgctctgctgaagccagttaccttcggaaaaagagttggtagctcttgatccggcaaacaaaccaccgctggtagcggtggtttttttgtttgcaagcagcagattacgcgcagaaaaaaaggatctcaagaagatcctttgatcttttctacggggtctgacgctcagtggaacgaaaactcacgttaagggattttggtcatgcattctaggtgattatttgccgactaccttggtgatctcgcctttcacgtagtggacaaattcttccaactgatctgcgcgcgaggccaagcgatcttcttcttgtccaagataagcctgtctagcttcaagtatgacgggctgatactgggccggcaggcgctccattgcccagtcggcagcgacatccttcggcgcgattttgccggttactgcgctgtaccaaatgcgggacaacgtaagcactacatttcgctcatcaccagcccagtcgggcggcgagttccatagcgttaaggtttcatttagcgcctcaaatagatcctgttcaggaaccggatcaaagagttcctccgccgctggacctaccaaggcaacgctatgttctcttgcttttgtcagcaagatagccagatcaatgtcgatcgtggctggctcgaagatacctgcaagaatgtcattgcgctgccattctccaaattgcagttcgcgcttagctggataacgccacggaatgatgtcgtcgtgcacaacaatggtgacttctacagcgcggagaatctcgctctctccaggggaagccgaagtttccaaaaggtcgttgatcaaagctcgccgcgttgtttcatcaagccttacggtcaccgtaaccagcaaatcaatatcactgtgtggcttcaggccgccatccactgcggagccgtacaaatgtacggccagcaacgtcggttcgagatggcgctcgatgacgccaactacctctgatagttgagtcgatacttcggcgatcaccgcttccctcataatgtttaactttgttttagggcgactgccctgctgcgtaacatcgttgctgctccataacatcaaacatcgacccacggcgtaacgcgcttgctgcttggatgcccgaggcatagactgtaccccaaaaaaacagtcataacaagccatgaaaaccgccactgcgccgttaccaccgctgcgttcggtcaaggttctggaccagttgcgtgagcgcatacgctacttgcattacagcttacgaaccgaacaggcttatgtccactgggttcgtgccttcatccgtttccacggtgtgcgtcacccggcaaccttgggtagcagcgaagtcgaggcatttctgtcctggctggaacagaacttattatttccttcctcttttctacagtatttaaagataccccaagaagctaattataacaagacgaactccaattcactgttccttgcattctaaaaccttaaataccagaaaacagctttttcaaagttgttttcaaagttggcgtataacatagtatcgacggagccgattttgaaaccgcggtgatcacaggcagcaacgctctgtcatcgttacaatcaacatgctaccctccgcgagatcatccgtgtttcaaacccggcagcttagttgccgttcttccgaatagcatcggtaacatgagcaaagtctgccgccttacaacggctctcccgctgacgccgtcccggactgatgggctgcctgtatcgagtggtgattttgtgccgagctgccggtcggggagctgttggctggctggtggcaggatatattgtggtgtaaacataacggatccggtctcaggagggagtcgattaaaaatcccaattatatttggtctaatttagtttggtattgagtaaaacaaattcgaaccaaaccaaaatataaatatatagtttttatatatatgcctttaagactttttatagaattttctttaaaaaatatctagaaatatttgcgactcttctggcatgtaatatttcgttaaatatgaagtgctccatttttattaactttaaataattggttgtacgatcactttcttatcaagtgttactaaaatgcgtcaatctctttgttcttccatattcatatgtcaaaatctatcaaaattcttatatatctttttcgaatttgaagtgaaatttcgataatttaaaattaaatagaacatatcattatttaggtatcatattgatttttatacttaattactaaatttggttaactttgaaagtgtacatcaacgaaaaattagtcaaacgactaaaataaataaatatcatgtgttattaagaaaattctcctataagaatattttaatagatcatatgtttgtaaaaaaaattaatttttactaacacatatatttacttatcaaaaatttgacaaagtaagattaaaataatattcatctaacaaaaaaaaaaccagaaaatgctgaaaacccggcaaaaccgaaccaatccaaaccgatatagttggtttggtttgattttgatataaaccgaaccaactcggtccatttgcacccctaatcataatagctttaatatttcaagatattattaagttaacgttgtcaatatcctggaaattttgcaaaatgaatcaagcctatatggctgtaatatgaatttaaaagcagctcgatgtggtggtaatatgtaatttacttgattctaaaaaaatatcccaagtattaataatttctgctaggaagaaggttagctacgatttacagcaaagccagaatacaaagaaccataaagtgattgaagctcgaaatatacgaaggaacaaatatttttaaaaaaatacgcaatgacttggaacaaaagaaagtgatatattttttgttcttaaacaagcatcccctctaaagaatggcagttttcctttgcatgtaactattatgctcccttcgttacaaaaattttggactactattgggaacttcttctgaaaatagtcgctccatggagccctcaattgtactaccatcatttcttgttccgctgcttggaggtgactccgaggggttgcctcaaactctatcttataaccggcgtggaggcatggaggcaggggtattttggtcattttaatagatagtggaaaatgacgtggaatttacttaaagacgaagtctttgcgacaagggggggcccacgccgaatttaatattaccggcgtggcccccccttatcgcgagtgctttagcacgagcggtccagatttaaagtagaaaatttcccgcccactagggttaaaggtgttcacactataaaagcatatacgatgtgatggtatttgctcgctggtaggagggaaacctcctcggattccattgcccagctatctgtcactttattgagaagatagtggaaaaggaaggtggctcctacaaatgccatcattgcgataaaggaaaggccatcgttgaagatgcctctgccgacagtggtcccaaagatggacccccacccacgaggagcatcgtggaaaaagaagacgttccaaccacgtcttcaaagcaagtggattgatgtgatatctccactgacgtaagggatgacgcacaatcccactatccttcgcaagacccttcctctatataaggaagttcatttcatttggagaggtattaaaatcttaataggttttgataaaagcgaacgtggggaaacccgaaccaaaccttcttctaaactctctctcatctctcttaaagcaaacttctctcttgtctttcttgcgtgagcgatcttcaacgttgtcagatcgtgcttcggcaccagtacaacgttttctttcactgaagcgaaatcaaagatctctttgtggacacgtagtgcggcgccattaaataacgtgtacttgtcctattcttgtcggtgtggtcttgggaaaagaaagcttgctggaggctgctgttcagccccatacattacttgttacgattctgctgactttcggcgggtgcaatatctctacttctgcttgacgaggtattgttgcctgtacttctttcttcttcttcttgctgattggttctataagaaatctagtattttctttgaaacagagttttcccgtggttttcgaacttggagaaagattgttaagcttctgtatattctgcccaaattcgcgaccggtaatggtgagcaagggcgaggagctgttcaccggggtggtgcccatcctggtcgagctggacggcgacgtaaacggccacaagttcagcgtgtccggcgagggcgagggcgatgccacctacggcaagctgaccctgaagttcatctgcaccaccggcaagctgcccgtgccctggcccaccctcgtgaccaccttcagctacggcgtgcagtgcttcagccgctaccccgaccacatgaagcagcacgacttcttcaagtccgccatgcccgaaggctacgtccaggagcgcaccatcttcttcaaggacgacggcaactacaagacccgcgccgaggtgaagttcgagggcgacaccctggtgaaccgcatcgagctgaagggcatcgacttcaaggaggacggcaacatcctggggcacaagctggagtacaactacaacagccacaacgtctatatcatggccgacaagcagaagaacggcatcaaggtgaacttcaagatccgccacaacatcgaggacggcagcgtgcagctcgccgaccactaccagcagaacacccccatcggcgacggccccgtgctgctgcccgacaaccactacctgagcacccagtccgccctgagcaaagaccccaacgagaagcgcgatcacatggtcctgctggagttcgtgaccgccgccgggatcactcacggcatggacgagctgtacaagtaagctttcccgggcatcaccatcaccatcactagctcgaggcctttaactctggtttcattaaattttctttagtttgaatttactgttattcggtgtgcatttctatgtttggtgagcggttttctgtgctcagagtgtgtttattttatgtaatttaatttctttgtgagctcctgtttagcaggtcgtcccttcagcaaggacacaaaaagattttaattttattaaaaaaaaaaaaaaaaaagaccgggaattcgatatcaagcttatcgacctgcagatcgttcaaacatttggcaataaagtttcttaagattgaatcctgttgccggtcttgcggtgattatcatataatttctgttgaattacgttaagcatgtaataattaacatgtaatgcatgacgttatttatgagatgggtttttatgattagagtcccgcaattatacatttaatacgcgatagaaaacaaaatatagcgcgcaaactaggataaattatcgcgcgcggtgtcatctatgttactagatccgctgtcaaaactctagtcaataccatcacatcgtatatgcttgtatagtgtgaacacctttaaccctagtgggcgggaaattttctactttaaatctggaccgctcgtgctaaagcactcgcgataagggggggccacgccggtaatattaaattcggcgtgggcccccccttgtcgcaaagacttcgtctttaagtaaattccacgtcattttccactatctattaaaatgaccaaaatacccctgcctccatgcctccacgccggttataagatagagtttgaggcaacccctcggagtcacaacaaatgccttctgctagtaagaacttcagactccaatctaaatatgttttccttacctatcccaagtgctcatctcaaagagatgatttattccagtttctctgggagaaactcacaccttttcttattttcttccttggtgttgcttctgagcttcatcaagatggcactacccactatcatgctcttctccagcttgataaaaaaccttgtattagggatccttcttttttcgattttgaaggaaatcaccctaatatccagccagctagaaactctaaacaagtccttgattacatatcaaaggacggagatattaaaaccagaggagatttccgagatcataaggtatctcctcgcaaatctgacgcacgatggagaactattatccagactgcaacgtctaaggaggaatatcttgacatgatcaaggaagaattccctcatgaatgggcaacaaaacttcaatggctggaatattcagccaacaaattattccctccacaacctgaaccgtatgtgtcgcccttcacagaatcagatcttcgctgccacgaagatctacactcctggagggaaacccatctataccatgtaagcatagacgcttatacttacatacatcctgtctcataccaacaagctcaatctgaccttgaatggatggccgatttaaccaggacaatggaaggaatggaatccgacaccccagcctctacatctgcggaccaactcgtaccggaaagaccacctgggctagaagtctcggacgacacaactattggaacggtaccatcgatttcaccaactacgatgaacacgccacctataatatcatcgacgacatccccttcaagttcgtcccattgtggaagcaattaataggttgccagtctgatttcactgtcaaccctaaatatggaaaaaagaagaaaataaaaggtgggatcccttctataattctttgcaatcctgacgaagactggatgttatcaatgacaagtcaacagaaggattactttaaagataattgcgtcacccactatatgtgtgacggggagactttttttgctcgggaatcgtcgagtcactgaaacgtgcctctcctcatacgagtttatctaaagtgattattttttttggggtgtttgtttttggattgtctttttttgtttatttcgtgtgttatgtaacatatgtaatttctatctacttgcacaatgaaatatattcataaaataatcatt

**pGB-E-DsRed-GFP**

gtcatgagaccggatcctgacaggatatattggcgggtaaacctaagagaaaagagcgtttattagaataatcggatatttaaaagggcgtgaaaaggtttatccgttcgtccatttgtatgtgcatgccaaccacagggttcccctcgggatcaaagtactttgatccaacccctccgctgctatagtgcagtcggcttctgacgttcagtgcagccgtcatctgaaaacgacatgtcgcacaagtcctaagttacgcgacaggctgccgccctgcccttttcctggcgttttcttgtcgcgtgttttagtcgcataaagtagaatacttgcgactagaaccggagacattacgccatgaacaagagcgccgccgctggcctgctgggctatgcccgcgtcagcaccgacgaccaggacttgaccaaccaacgggccgaactgcacgcggccggctgcaccaagctgttttccgagaagatcaccggcaccaggcgcgaccgcccggagctggccaggatgcttgaccacctacgccctggcgacgttgtgacagtgaccaggctagaccgcctggcccgcagcacccgcgacctactggacattgccgagcgcatccaggaggccggcgcgggcctgcgtagcctggcagagccgtgggccgacaccaccacgccggccggccgcatggtgttgaccgtgttcgccggcattgccgagttcgagcgttccctaatcatcgaccgcacccggagcgggcgcgaggccgccaaggcccgaggcgtgaagtttggcccccgccctaccctcaccccggcacagatcgcgcacgcccgcgagctgatcgaccaggaaggccgcaccgtgaaagaggcggctgcactgcttggcgtgcatcgctcgaccctgtaccgcgcacttgagcgcagcgaggaagtgacgcccaccgaggccaggcggcgcggtgccttccgtgaggacgcattgaccgaggccgacgccctggcggccgccgagaatgaacgccaagaggaacaagcatgaaaccgcaccaggacggccaggacgaaccgtttttcattaccgaagagatcgaggcggagatgatcgcggccgggtacgtgttcgagccgcccgcgcacctctcaaccgtgcggctgcatgaaatcctggccggtttgtctgatgccaagctggcggcctggccggccagcttggccgctgaagaaaccgagcgccgccgtctaaaaaggtgatgtgtatttgagtaaaacagcttgcgtcatgcggtcgctgcgtatatgatccgatgagtaaataaacaaatacgcaaggggaacgcatgaaggttatcgctgtacttaaccagaaaggcgggtcaggcaagacgaccatcggaacccatctagcccgcgccctgcaactcgccggggccgatgttctgttagtcgattccgatccccagggcagtgcccgcgattgggcggccgtgcgggaagatcaaccgctaaccgttgtcggcatcgaccgcccgacgattgaccgcgacgtgaaggccatcggccggcgcgacttcgtagtgatcgacggagcgccccaggcggcggacttggctgtgtccgcgatcaaggcagccgacttcgtgctgattccggtgcagccaagcccttacgacatatgggccaccgccgacctggtggagctggttaagcagcgcattgaggtcacggatggaaggctacaagcggcctttgtcgtgtcgcgggcgatcaaaggcacgcgcatcggcggtgaggttgccgaggcgctggccgggtacgagctgcccattcttgagtcccgtatcacgcagcgcgtgagctacccaggcactgccgccgccggcacaaccgttcttgaatcagaacccgagggcgacgctgcccgcgaggtccaggcgctggccgctgaaattaaatcaaaactcatttgagttaatgaggtaaagagaaaatgagcaaaagcacaaacacgctaagtgccggccgtccgagcgcacgcagcagcaaggctgcaacgttggccagcctggcagacacgccagccatgaagcgggtcaactttcagttgccggcggaggatcacaccaagctgaagatgtacgcggtacgccaaggcaagaccattaccgagctgctatctgaatagatcgcgcagctaccagagtaaatgagcaaatgaataaatgagtagatgaattttagcggctaaaggaggcggcatggaaaatcaagaacaaccaggcaccgacgccgtggaatgccccatgtgtggaggaacgggcggttggccaggcgtaagcggctgggttgtctgccggccctgcaatggcactggaacccccaagcccgaggaatcggcgtgacggtcgcaaaccatccggcccggtacaaatcggcgcggcgctgggtgatgacctggtggagaagttgaaggccgcgcaggccgcccagcggcaacgcatcgaggcagaagcacgccccggtgaatcgtggcaagcggccgctgatcgaatccgcaaagaatcccggcaaccgccggcagccggtgcgccgtcgattaggaagccgcccaagggcgacgagcaaccagattttttcgttccgatgctctatgacgtgggcacccgcgatagtcgcagcatcatggacgtggccgttttccgtctgtcgaagcgtgaccgacgagctggcgaggtgatccgctacgagcttccagacgggcacgtagaggtttccgcagggccggccggcatggccagtgtgtgggattacgacctggtactgatggcggtttcccatctaaccgaatccatgaaccgataccgggaagggaagggagacaagcccggccgcgtgttccgtccacacgttgcggacgtactcaagttctgccggcgagccgatggcggaaagcagaaagacgacctggtagaaacctgcattcggttaaacaccacgcacgttgccatgcagcgtacgaagaaggccaagaacggccgcctggtgacggtatccgagggtgaagccttgattagccgctacaagatcgtaaagagcgaaaccgggcggccggagtacatcgagatcgagctagctgattggatgtaccgcgagatcacagaaggcaagaacccggacgtgctgacggttcaccccgattactttttgatcgatcccggcatcggccgttttctctaccgcctggcacgccgcgccgcaggcaaggcagaagccagatggttgttcaagacgatctacgaacgcagtggcagcgccggagagttcaagaagttctgtttcaccgtgcgcaagctgatcgggtcaaatgacctgccggagtacgatttgaaggaggaggcggggcaggctggcccgatcctagtcatgcgctaccgcaacctgatcgagggcgaagcatccgccggttcctaatgtacggagcagatgctagggcaaattgccctagcaggggaaaaaggtcgaaaaggactctttcctgtggatagcacgtacattgggaacccaaagccgtacattgggaaccggaacccgtacattgggaacccaaagccgtacattgggaaccggtcacacatgtaagtgactgatataaaagagaaaaaaggcgatttttccgcctaaaactctttaaaacttattaaaactcttaaaacccgcctggcctgtgcataactgtctggccagcgcacagccgaagagctgcaaaaagcgcctacccttcggtcgctgcgctccctacgccccgccgcttcgcgtcggcctatcgcggccgctggccgctcaaaaatggctggcctacggccaggcaatctaccagggcgcggacaagccgcgccgtcgccactcgaccgccggcgcccacatcaaggcaccctgcctcgcgcgtttcggtgatgacggtgaaaacctctgacacatgcagctcccggtgacggtcacagcttgtctgtaagcggatgccgggagcagacaagcccgtcagggcgcgtcagcgggtgttggcgggtgtcggggcgcagccatgacccagtcacgtagcgatagcggagtgtatactggcttaactatgcggcatcagagcagattgtactgagagtgcaccatatgcggtgtgaaataccgcacagatgcgtaaggagaaaataccgcatcaggcgctcttccgcttcctcgctcactgactcgctgcgctcggtcgttcggctgcggcgagcggtatcagctcactcaaaggcggtaatacggttatccacagaatcaggggataacgcaggaaagaacatgtgagcaaaaggccagcaaaaggccaggaaccgtaaaaaggccgcgttgctggcgtttttccataggctccgcccccctgacgagcatcacaaaaatcgacgctcaagtcagaggtggcgaaacccgacaggactataaagataccaggcgtttccccctggaagctccctcgtgcgctctcctgttccgaccctgccgcttaccggatacctgtccgcctttctcccttcgggaagcgtggcgctttctcatagctcacgctgtaggtatctcagttcggtgtaggtcgttcgctccaagctgggctgtgtgcacgaaccccccgttcagcccgaccgctgcgccttatccggtaactatcgtcttgagtccaacccggtaagacacgacttatcgccactggcagcagccactggtaacaggattagcagagcgaggtatgtaggcggtgctacagagttcttgaagtggtggcctaactacggctacactagaaggacagtatttggtatctgcgctctgctgaagccagttaccttcggaaaaagagttggtagctcttgatccggcaaacaaaccaccgctggtagcggtggtttttttgtttgcaagcagcagattacgcgcagaaaaaaaggatctcaagaagatcctttgatcttttctacggggtctgacgctcagtggaacgaaaactcacgttaagggattttggtcatgcattctaggtgattatttgccgactaccttggtgatctcgcctttcacgtagtggacaaattcttccaactgatctgcgcgcgaggccaagcgatcttcttcttgtccaagataagcctgtctagcttcaagtatgacgggctgatactgggccggcaggcgctccattgcccagtcggcagcgacatccttcggcgcgattttgccggttactgcgctgtaccaaatgcgggacaacgtaagcactacatttcgctcatcaccagcccagtcgggcggcgagttccatagcgttaaggtttcatttagcgcctcaaatagatcctgttcaggaaccggatcaaagagttcctccgccgctggacctaccaaggcaacgctatgttctcttgcttttgtcagcaagatagccagatcaatgtcgatcgtggctggctcgaagatacctgcaagaatgtcattgcgctgccattctccaaattgcagttcgcgcttagctggataacgccacggaatgatgtcgtcgtgcacaacaatggtgacttctacagcgcggagaatctcgctctctccaggggaagccgaagtttccaaaaggtcgttgatcaaagctcgccgcgttgtttcatcaagccttacggtcaccgtaaccagcaaatcaatatcactgtgtggcttcaggccgccatccactgcggagccgtacaaatgtacggccagcaacgtcggttcgagatggcgctcgatgacgccaactacctctgatagttgagtcgatacttcggcgatcaccgcttccctcataatgtttaactttgttttagggcgactgccctgctgcgtaacatcgttgctgctccataacatcaaacatcgacccacggcgtaacgcgcttgctgcttggatgcccgaggcatagactgtaccccaaaaaaacagtcataacaagccatgaaaaccgccactgcgccgttaccaccgctgcgttcggtcaaggttctggaccagttgcgtgagcgcatacgctacttgcattacagcttacgaaccgaacaggcttatgtccactgggttcgtgccttcatccgtttccacggtgtgcgtcacccggcaaccttgggtagcagcgaagtcgaggcatttctgtcctggctggaacagaacttattatttccttcctcttttctacagtatttaaagataccccaagaagctaattataacaagacgaactccaattcactgttccttgcattctaaaaccttaaataccagaaaacagctttttcaaagttgttttcaaagttggcgtataacatagtatcgacggagccgattttgaaaccgcggtgatcacaggcagcaacgctctgtcatcgttacaatcaacatgctaccctccgcgagatcatccgtgtttcaaacccggcagcttagttgccgttcttccgaatagcatcggtaacatgagcaaagtctgccgccttacaacggctctcccgctgacgccgtcccggactgatgggctgcctgtatcgagtggtgattttgtgccgagctgccggtcggggagctgttggctggctggtggcaggatatattgtggtgtaaacataacggatccggtctcaggagtcgattaaaaatcccaattatatttggtctaatttagtttggtattgagtaaaacaaattcgaaccaaaccaaaatataaatatatagtttttatatatatgcctttaagactttttatagaattttctttaaaaaatatctagaaatatttgcgactcttctggcatgtaatatttcgttaaatatgaagtgctccatttttattaactttaaataattggttgtacgatcactttcttatcaagtgttactaaaatgcgtcaatctctttgttcttccatattcatatgtcaaaatctatcaaaattcttatatatctttttcgaatttgaagtgaaatttcgataatttaaaattaaatagaacatatcattatttaggtatcatattgatttttatacttaattactaaatttggttaactttgaaagtgtacatcaacgaaaaattagtcaaacgactaaaataaataaatatcatgtgttattaagaaaattctcctataagaatattttaatagatcatatgtttgtaaaaaaaattaatttttactaacacatatatttacttatcaaaaatttgacaaagtaagattaaaataatattcatctaacaaaaaaaaaaccagaaaatgctgaaaacccggcaaaaccgaaccaatccaaaccgatatagttggtttggtttgattttgatataaaccgaaccaactcggtccatttgcacccctaatcataatagctttaatatttcaagatattattaagttaacgttgtcaatatcctggaaattttgcaaaatgaatcaagcctatatggctgtaatatgaatttaaaagcagctcgatgtggtggtaatatgtaatttacttgattctaaaaaaatatcccaagtattaataatttctgctaggaagaaggttagctacgatttacagcaaagccagaatacaaagaaccataaagtgattgaagctcgaaatatacgaaggaacaaatatttttaaaaaaatacgcaatgacttggaacaaaagaaagtgatatattttttgttcttaaacaagcatcccctctaaagaatggcagttttcctttgcatgtaactattatgctcccttcgttacaaaaattttggactactattgggaacttcttctgaaaatagtcgctccatccatggagggaaacctcctcggattccattgcccagctatctgtcactttattgagaagatagtggaaaaggaaggtggctcctacaaatgccatcattgcgataaaggaaaggccatcgttgaagatgcctctgccgacagtggtcccaaagatggacccccacccacgaggagcatcgtggaaaaagaagacgttccaaccacgtcttcaaagcaagtggattgatgtgatatctccactgacgtaagggatgacgcacaatcccactatccttcgcaagacccttcctctatataaggaagttcatttcatttggagaggtattaaaatcttaataggttttgataaaagcgaacgtggggaaacccgaaccaaaccttcttctaaactctctctcatctctcttaaagcaaacttctctcttgtctttcttgcgtgagcgatcttcaacgttgtcagatcgtgcttcggcaccagtacaacgttttctttcactgaagcgaaatcaaagatctctttgtggacacgtagtgcggcgccattaaataacgtgtacttgtcctattcttgtcggtgtggtcttgggaaaagaaagcttgctggaggctgctgttcagccccatacattacttgttacgattctgctgactttcggcgggtgcaatatctctacttctgcttgacgaggtattgttgcctgtacttctttcttcttcttcttgctgattggttctataagaaatctagtattttctttgaaacagagttttcccgtggttttcgaacttggagaaagattgttaagcttctgtatattctgcccaaattcgcgaccggtaatggggtcatccaagaatgttatcaaggagttcatgaggtttaaggttcgcatggaaggaacggtcaatgggcacgagtttgaaatagaaggcgaaggagaggggaggccatacgaaggccacaataccgtaaagcttaaggtaaccaaggggggacctttgccatttgcttgggatattttgtcaccacaatttcagtatggaagcaaggtatatgtcaagcaccctgccgacataccagactataaaaagctgtcatttcctgaaggatttaaatgggaaagggtcatgaactttgaagatggtggcgtcgttactgtaacccaggattccagtttgcaggatggctgtttcatctacaaggtcaagttcattggcgtgaactttccttccgatggacctgttatgcaaaagaaaacaatgggctgggaagccagcactgagcgtttgtatcctcgtgatggcgtgttgaaaggagagattcataaggctctgaagctgaaagacggtggtcattacctagttgaattcaaaagtatttacatggcaaagaagcctgtgcagctaccagggtactactatgttgactccaaactggatataacaagccacaacgaagattatacaatcgttgagcagtatgaaagaaccgagggacgccaccatctgttcctttaagctttcccgggcatcaccatcaccatcactagctcgaggcctttaactctggtttcattaaattttctttagtttgaatttactgttattcggtgtgcatttctatgtttggtgagcggttttctgtgctcagagtgtgtttattttatgtaatttaatttctttgtgagctcctgtttagcaggtcgtcccttcagcaaggacacaaaaagattttaattttattaaaaaaaaaaaaaaaaaagaccgggaattcgatatcaagcttatcgacctgcagatcgttcaaacatttggcaataaagtttcttaagattgaatcctgttgccggtcttgcggtgattatcatataatttctgttgaattacgttaagcatgtaataattaacatgtaatgcatgacgttatttatgagatgggtttttatgattagagtcccgcaattatacatttaatacgcgatagaaaacaaaatatagcgcgcaaactaggataaattatcgcgcgcggtgtcatctatgttactagatccgctgcttggagggaaacctcctcggattccattgcccagctatctgtcactttattgagaagatagtggaaaaggaaggtggctcctacaaatgccatcattgcgataaaggaaaggccatcgttgaagatgcctctgccgacagtggtcccaaagatggacccccacccacgaggagcatcgtggaaaaagaagacgttccaaccacgtcttcaaagcaagtggattgatgtgatatctccactgacgtaagggatgacgcacaatcccactatccttcgcaagacccttcctctatataaggaagttcatttcatttggagaggtattaaaatcttaataggttttgataaaagcgaacgtggggaaacccgaaccaaaccttcttctaaactctctctcatctctcttaaagcaaacttctctcttgtctttcttgcgtgagcgatcttcaacgttgtcagatcgtgcttcggcaccagtacaacgttttctttcactgaagcgaaatcaaagatctctttgtggacacgtagtgcggcgccattaaataacgtgtacttgtcctattcttgtcggtgtggtcttgggaaaagaaagcttgctggaggctgctgttcagccccatacattacttgttacgattctgctgactttcggcgggtgcaatatctctacttctgcttgacgaggtattgttgcctgtacttctttcttcttcttcttgctgattggttctataagaaatctagtattttctttgaaacagagttttcccgtggttttcgaacttggagaaagattgttaagcttctgtatattctgcccaaattcgcgaccggtaatggtgagcaagggcgaggagctgttcaccggggtggtgcccatcctggtcgagctggacggcgacgtaaacggccacaagttcagcgtgtccggcgagggcgagggcgatgccacctacggcaagctgaccctgaagttcatctgcaccaccggcaagctgcccgtgccctggcccaccctcgtgaccaccttcagctacggcgtgcagtgcttcagccgctaccccgaccacatgaagcagcacgacttcttcaagtccgccatgcccgaaggctacgtccaggagcgcaccatcttcttcaaggacgacggcaactacaagacccgcgccgaggtgaagttcgagggcgacaccctggtgaaccgcatcgagctgaagggcatcgacttcaaggaggacggcaacatcctggggcacaagctggagtacaactacaacagccacaacgtctatatcatggccgacaagcagaagaacggcatcaaggtgaacttcaagatccgccacaacatcgaggacggcagcgtgcagctcgccgaccactaccagcagaacacccccatcggcgacggccccgtgctgctgcccgacaaccactacctgagcacccagtccgccctgagcaaagaccccaacgagaagcgcgatcacatggtcctgctggagttcgtgaccgccgccgggatcactcacggcatggacgagctgtacaagtaagctttcccgggcatcaccatcaccatcactagctcgaggcctttaactctggtttcattaaattttctttagtttgaatttactgttattcggtgtgcatttctatgtttggtgagcggttttctgtgctcagagtgtgtttattttatgtaatttaatttctttgtgagctcctgtttagcaggtcgtcccttcagcaaggacacaaaaagattttaattttattaaaaaaaaaaaaaaaaaagaccgggaattcgatatcaagcttatcgacctgcagatcgttcaaacatttggcaataaagtttcttaagattgaatcctgttgccggtcttgcggtgattatcatataatttctgttgaattacgttaagcatgtaataattaacatgtaatgcatgacgttatttatgagatgggtttttatgattagagtcccgcaattatacatttaatacgcgatagaaaacaaaatatagcgcgcaaactaggataaattatcgcgcgcggtgtcatctatgttactagatccgctggtaggaggtgaattctaaacttatattgagatattagtgtataatataatttccgcactctcttttaaattaataatacaagatttagaaaaaatgaactttaattttgagatattagtgtgtaattctcagtagagaatttcctaagttcacccaaaagtatatcattttcctcttaagaaaatacaaacactacctaattttatcccctataaatatctaaaaatttgcatctcataaaatttaccaattatttattttttaagatattttactaattatctataactattaataatcaaaattatttcattgatgtacatatttcaatagataatttaccccttaatcacttaataaattttaaattttcattatttttatataatttatagtcttttttattaactatatttaaattttattttttattattaaaaaaatttagagagacacatttttccctaattagtcatatataagaaaaataacatttgggtaaaatgtgagagcccaaacgcaattcgtgttgggcctaaagggcccacgaagtagatactaaaggatgccctcatcgatgaatgcttcgctgtcaggagggaggtcaacatggtggagcacgacactctggtctactccaaaaatgtcaaagatacagtctcagaagatcaaagggctattgagacttttcaacaaaggataatttcgggaaacctcctcggattccattgcccagctatctgtcacttcatcgaaaggacagtagaaaaggaaggtggctcctacaaatgccatcattgcgataaaggaaaggctatcattcaagatctctctgccgacagtggtcccaaagatggacccccacccacgaggagcatcgtggaaaaagaagaggttccaaccacgtctacaaagcaagtggattgatgtgacatctccactgacgtaagggatgacgcacaatcccactatccttcgcaagacccttcctctatataaggaagttcatttcatttggagaggacacgctcgagtataagagctcatttttacaacaattaccaacaacaacaaacaacaaacaacattacaattacatttacaattatcgatacaatggaacgagctatacaaggaaacgacgctagggaacaagctaacagtgaacgttgggatggaggatcaggaggtaccacttctcccttcaaacttcctgacgaaagtccgagttggactgagtggcggctacataacgatgagactaattcgaatcaagataatccccttggtttcaaggaaagctggggtttcgggaaagttgtatttaagagatatctcagatacgacaggacggaagcttcactgcacagagtccttggatcttggacgggagattcggttaactatgcagcatctcgatttttcggtttcgaccagatcggatgtacctatagtattcggtttcgaggagttagtatcaccgtttctggaggctctcgaactcttcagcatctctgtgagatggcaattcggtctaagcaagaactgctacagcttgccccaatcgaagtggaaagtaatgtatcaagaggatgccctgaaggtactgaaaccttcgaaaaagaaagcgagtgagcttgtcaagcagatcgttcaaacatttggcaataaagtttcttaagattgaatcctgttgccggtcttgcgatgattatcatataatttctgttgaattacgttaagcatgtaataattaacatgtaatgcatgacgttatttatgagatgggtttttatgattagagtcccgcaattatacatttaatacgcgatagaaaacaaaatatagcgcgcaaactaggataaattatcgcgcgcggtgtcatctatgttactagatcgacgct

**pGB-E-GFP-DsRed**

gtcatgagaccggatcctgacaggatatattggcgggtaaacctaagagaaaagagcgtttattagaataatcggatatttaaaagggcgtgaaaaggtttatccgttcgtccatttgtatgtgcatgccaaccacagggttcccctcgggatcaaagtactttgatccaacccctccgctgctatagtgcagtcggcttctgacgttcagtgcagccgtcatctgaaaacgacatgtcgcacaagtcctaagttacgcgacaggctgccgccctgcccttttcctggcgttttcttgtcgcgtgttttagtcgcataaagtagaatacttgcgactagaaccggagacattacgccatgaacaagagcgccgccgctggcctgctgggctatgcccgcgtcagcaccgacgaccaggacttgaccaaccaacgggccgaactgcacgcggccggctgcaccaagctgttttccgagaagatcaccggcaccaggcgcgaccgcccggagctggccaggatgcttgaccacctacgccctggcgacgttgtgacagtgaccaggctagaccgcctggcccgcagcacccgcgacctactggacattgccgagcgcatccaggaggccggcgcgggcctgcgtagcctggcagagccgtgggccgacaccaccacgccggccggccgcatggtgttgaccgtgttcgccggcattgccgagttcgagcgttccctaatcatcgaccgcacccggagcgggcgcgaggccgccaaggcccgaggcgtgaagtttggcccccgccctaccctcaccccggcacagatcgcgcacgcccgcgagctgatcgaccaggaaggccgcaccgtgaaagaggcggctgcactgcttggcgtgcatcgctcgaccctgtaccgcgcacttgagcgcagcgaggaagtgacgcccaccgaggccaggcggcgcggtgccttccgtgaggacgcattgaccgaggccgacgccctggcggccgccgagaatgaacgccaagaggaacaagcatgaaaccgcaccaggacggccaggacgaaccgtttttcattaccgaagagatcgaggcggagatgatcgcggccgggtacgtgttcgagccgcccgcgcacctctcaaccgtgcggctgcatgaaatcctggccggtttgtctgatgccaagctggcggcctggccggccagcttggccgctgaagaaaccgagcgccgccgtctaaaaaggtgatgtgtatttgagtaaaacagcttgcgtcatgcggtcgctgcgtatatgatccgatgagtaaataaacaaatacgcaaggggaacgcatgaaggttatcgctgtacttaaccagaaaggcgggtcaggcaagacgaccatcggaacccatctagcccgcgccctgcaactcgccggggccgatgttctgttagtcgattccgatccccagggcagtgcccgcgattgggcggccgtgcgggaagatcaaccgctaaccgttgtcggcatcgaccgcccgacgattgaccgcgacgtgaaggccatcggccggcgcgacttcgtagtgatcgacggagcgccccaggcggcggacttggctgtgtccgcgatcaaggcagccgacttcgtgctgattccggtgcagccaagcccttacgacatatgggccaccgccgacctggtggagctggttaagcagcgcattgaggtcacggatggaaggctacaagcggcctttgtcgtgtcgcgggcgatcaaaggcacgcgcatcggcggtgaggttgccgaggcgctggccgggtacgagctgcccattcttgagtcccgtatcacgcagcgcgtgagctacccaggcactgccgccgccggcacaaccgttcttgaatcagaacccgagggcgacgctgcccgcgaggtccaggcgctggccgctgaaattaaatcaaaactcatttgagttaatgaggtaaagagaaaatgagcaaaagcacaaacacgctaagtgccggccgtccgagcgcacgcagcagcaaggctgcaacgttggccagcctggcagacacgccagccatgaagcgggtcaactttcagttgccggcggaggatcacaccaagctgaagatgtacgcggtacgccaaggcaagaccattaccgagctgctatctgaatagatcgcgcagctaccagagtaaatgagcaaatgaataaatgagtagatgaattttagcggctaaaggaggcggcatggaaaatcaagaacaaccaggcaccgacgccgtggaatgccccatgtgtggaggaacgggcggttggccaggcgtaagcggctgggttgtctgccggccctgcaatggcactggaacccccaagcccgaggaatcggcgtgacggtcgcaaaccatccggcccggtacaaatcggcgcggcgctgggtgatgacctggtggagaagttgaaggccgcgcaggccgcccagcggcaacgcatcgaggcagaagcacgccccggtgaatcgtggcaagcggccgctgatcgaatccgcaaagaatcccggcaaccgccggcagccggtgcgccgtcgattaggaagccgcccaagggcgacgagcaaccagattttttcgttccgatgctctatgacgtgggcacccgcgatagtcgcagcatcatggacgtggccgttttccgtctgtcgaagcgtgaccgacgagctggcgaggtgatccgctacgagcttccagacgggcacgtagaggtttccgcagggccggccggcatggccagtgtgtgggattacgacctggtactgatggcggtttcccatctaaccgaatccatgaaccgataccgggaagggaagggagacaagcccggccgcgtgttccgtccacacgttgcggacgtactcaagttctgccggcgagccgatggcggaaagcagaaagacgacctggtagaaacctgcattcggttaaacaccacgcacgttgccatgcagcgtacgaagaaggccaagaacggccgcctggtgacggtatccgagggtgaagccttgattagccgctacaagatcgtaaagagcgaaaccgggcggccggagtacatcgagatcgagctagctgattggatgtaccgcgagatcacagaaggcaagaacccggacgtgctgacggttcaccccgattactttttgatcgatcccggcatcggccgttttctctaccgcctggcacgccgcgccgcaggcaaggcagaagccagatggttgttcaagacgatctacgaacgcagtggcagcgccggagagttcaagaagttctgtttcaccgtgcgcaagctgatcgggtcaaatgacctgccggagtacgatttgaaggaggaggcggggcaggctggcccgatcctagtcatgcgctaccgcaacctgatcgagggcgaagcatccgccggttcctaatgtacggagcagatgctagggcaaattgccctagcaggggaaaaaggtcgaaaaggactctttcctgtggatagcacgtacattgggaacccaaagccgtacattgggaaccggaacccgtacattgggaacccaaagccgtacattgggaaccggtcacacatgtaagtgactgatataaaagagaaaaaaggcgatttttccgcctaaaactctttaaaacttattaaaactcttaaaacccgcctggcctgtgcataactgtctggccagcgcacagccgaagagctgcaaaaagcgcctacccttcggtcgctgcgctccctacgccccgccgcttcgcgtcggcctatcgcggccgctggccgctcaaaaatggctggcctacggccaggcaatctaccagggcgcggacaagccgcgccgtcgccactcgaccgccggcgcccacatcaaggcaccctgcctcgcgcgtttcggtgatgacggtgaaaacctctgacacatgcagctcccggtgacggtcacagcttgtctgtaagcggatgccgggagcagacaagcccgtcagggcgcgtcagcgggtgttggcgggtgtcggggcgcagccatgacccagtcacgtagcgatagcggagtgtatactggcttaactatgcggcatcagagcagattgtactgagagtgcaccatatgcggtgtgaaataccgcacagatgcgtaaggagaaaataccgcatcaggcgctcttccgcttcctcgctcactgactcgctgcgctcggtcgttcggctgcggcgagcggtatcagctcactcaaaggcggtaatacggttatccacagaatcaggggataacgcaggaaagaacatgtgagcaaaaggccagcaaaaggccaggaaccgtaaaaaggccgcgttgctggcgtttttccataggctccgcccccctgacgagcatcacaaaaatcgacgctcaagtcagaggtggcgaaacccgacaggactataaagataccaggcgtttccccctggaagctccctcgtgcgctctcctgttccgaccctgccgcttaccggatacctgtccgcctttctcccttcgggaagcgtggcgctttctcatagctcacgctgtaggtatctcagttcggtgtaggtcgttcgctccaagctgggctgtgtgcacgaaccccccgttcagcccgaccgctgcgccttatccggtaactatcgtcttgagtccaacccggtaagacacgacttatcgccactggcagcagccactggtaacaggattagcagagcgaggtatgtaggcggtgctacagagttcttgaagtggtggcctaactacggctacactagaaggacagtatttggtatctgcgctctgctgaagccagttaccttcggaaaaagagttggtagctcttgatccggcaaacaaaccaccgctggtagcggtggtttttttgtttgcaagcagcagattacgcgcagaaaaaaaggatctcaagaagatcctttgatcttttctacggggtctgacgctcagtggaacgaaaactcacgttaagggattttggtcatgcattctaggtgattatttgccgactaccttggtgatctcgcctttcacgtagtggacaaattcttccaactgatctgcgcgcgaggccaagcgatcttcttcttgtccaagataagcctgtctagcttcaagtatgacgggctgatactgggccggcaggcgctccattgcccagtcggcagcgacatccttcggcgcgattttgccggttactgcgctgtaccaaatgcgggacaacgtaagcactacatttcgctcatcaccagcccagtcgggcggcgagttccatagcgttaaggtttcatttagcgcctcaaatagatcctgttcaggaaccggatcaaagagttcctccgccgctggacctaccaaggcaacgctatgttctcttgcttttgtcagcaagatagccagatcaatgtcgatcgtggctggctcgaagatacctgcaagaatgtcattgcgctgccattctccaaattgcagttcgcgcttagctggataacgccacggaatgatgtcgtcgtgcacaacaatggtgacttctacagcgcggagaatctcgctctctccaggggaagccgaagtttccaaaaggtcgttgatcaaagctcgccgcgttgtttcatcaagccttacggtcaccgtaaccagcaaatcaatatcactgtgtggcttcaggccgccatccactgcggagccgtacaaatgtacggccagcaacgtcggttcgagatggcgctcgatgacgccaactacctctgatagttgagtcgatacttcggcgatcaccgcttccctcataatgtttaactttgttttagggcgactgccctgctgcgtaacatcgttgctgctccataacatcaaacatcgacccacggcgtaacgcgcttgctgcttggatgcccgaggcatagactgtaccccaaaaaaacagtcataacaagccatgaaaaccgccactgcgccgttaccaccgctgcgttcggtcaaggttctggaccagttgcgtgagcgcatacgctacttgcattacagcttacgaaccgaacaggcttatgtccactgggttcgtgccttcatccgtttccacggtgtgcgtcacccggcaaccttgggtagcagcgaagtcgaggcatttctgtcctggctggaacagaacttattatttccttcctcttttctacagtatttaaagataccccaagaagctaattataacaagacgaactccaattcactgttccttgcattctaaaaccttaaataccagaaaacagctttttcaaagttgttttcaaagttggcgtataacatagtatcgacggagccgattttgaaaccgcggtgatcacaggcagcaacgctctgtcatcgttacaatcaacatgctaccctccgcgagatcatccgtgtttcaaacccggcagcttagttgccgttcttccgaatagcatcggtaacatgagcaaagtctgccgccttacaacggctctcccgctgacgccgtcccggactgatgggctgcctgtatcgagtggtgattttgtgccgagctgccggtcggggagctgttggctggctggtggcaggatatattgtggtgtaaacataacggatccggtctcaggagtcgattaaaaatcccaattatatttggtctaatttagtttggtattgagtaaaacaaattcgaaccaaaccaaaatataaatatatagtttttatatatatgcctttaagactttttatagaattttctttaaaaaatatctagaaatatttgcgactcttctggcatgtaatatttcgttaaatatgaagtgctccatttttattaactttaaataattggttgtacgatcactttcttatcaagtgttactaaaatgcgtcaatctctttgttcttccatattcatatgtcaaaatctatcaaaattcttatatatctttttcgaatttgaagtgaaatttcgataatttaaaattaaatagaacatatcattatttaggtatcatattgatttttatacttaattactaaatttggttaactttgaaagtgtacatcaacgaaaaattagtcaaacgactaaaataaataaatatcatgtgttattaagaaaattctcctataagaatattttaatagatcatatgtttgtaaaaaaaattaatttttactaacacatatatttacttatcaaaaatttgacaaagtaagattaaaataatattcatctaacaaaaaaaaaaccagaaaatgctgaaaacccggcaaaaccgaaccaatccaaaccgatatagttggtttggtttgattttgatataaaccgaaccaactcggtccatttgcacccctaatcataatagctttaatatttcaagatattattaagttaacgttgtcaatatcctggaaattttgcaaaatgaatcaagcctatatggctgtaatatgaatttaaaagcagctcgatgtggtggtaatatgtaatttacttgattctaaaaaaatatcccaagtattaataatttctgctaggaagaaggttagctacgatttacagcaaagccagaatacaaagaaccataaagtgattgaagctcgaaatatacgaaggaacaaatatttttaaaaaaatacgcaatgacttggaacaaaagaaagtgatatattttttgttcttaaacaagcatcccctctaaagaatggcagttttcctttgcatgtaactattatgctcccttcgttacaaaaattttggactactattgggaacttcttctgaaaatagtcgctccatccatggagggaaacctcctcggattccattgcccagctatctgtcactttattgagaagatagtggaaaaggaaggtggctcctacaaatgccatcattgcgataaaggaaaggccatcgttgaagatgcctctgccgacagtggtcccaaagatggacccccacccacgaggagcatcgtggaaaaagaagacgttccaaccacgtcttcaaagcaagtggattgatgtgatatctccactgacgtaagggatgacgcacaatcccactatccttcgcaagacccttcctctatataaggaagttcatttcatttggagaggtattaaaatcttaataggttttgataaaagcgaacgtggggaaacccgaaccaaaccttcttctaaactctctctcatctctcttaaagcaaacttctctcttgtctttcttgcgtgagcgatcttcaacgttgtcagatcgtgcttcggcaccagtacaacgttttctttcactgaagcgaaatcaaagatctctttgtggacacgtagtgcggcgccattaaataacgtgtacttgtcctattcttgtcggtgtggtcttgggaaaagaaagcttgctggaggctgctgttcagccccatacattacttgttacgattctgctgactttcggcgggtgcaatatctctacttctgcttgacgaggtattgttgcctgtacttctttcttcttcttcttgctgattggttctataagaaatctagtattttctttgaaacagagttttcccgtggttttcgaacttggagaaagattgttaagcttctgtatattctgcccaaattcgcgaccggtaatggtgagcaagggcgaggagctgttcaccggggtggtgcccatcctggtcgagctggacggcgacgtaaacggccacaagttcagcgtgtccggcgagggcgagggcgatgccacctacggcaagctgaccctgaagttcatctgcaccaccggcaagctgcccgtgccctggcccaccctcgtgaccaccttcagctacggcgtgcagtgcttcagccgctaccccgaccacatgaagcagcacgacttcttcaagtccgccatgcccgaaggctacgtccaggagcgcaccatcttcttcaaggacgacggcaactacaagacccgcgccgaggtgaagttcgagggcgacaccctggtgaaccgcatcgagctgaagggcatcgacttcaaggaggacggcaacatcctggggcacaagctggagtacaactacaacagccacaacgtctatatcatggccgacaagcagaagaacggcatcaaggtgaacttcaagatccgccacaacatcgaggacggcagcgtgcagctcgccgaccactaccagcagaacacccccatcggcgacggccccgtgctgctgcccgacaaccactacctgagcacccagtccgccctgagcaaagaccccaacgagaagcgcgatcacatggtcctgctggagttcgtgaccgccgccgggatcactcacggcatggacgagctgtacaagtaagctttcccgggcatcaccatcaccatcactagctcgaggcctttaactctggtttcattaaattttctttagtttgaatttactgttattcggtgtgcatttctatgtttggtgagcggttttctgtgctcagagtgtgtttattttatgtaatttaatttctttgtgagctcctgtttagcaggtcgtcccttcagcaaggacacaaaaagattttaattttattaaaaaaaaaaaaaaaaaagaccgggaattcgatatcaagcttatcgacctgcagatcgttcaaacatttggcaataaagtttcttaagattgaatcctgttgccggtcttgcggtgattatcatataatttctgttgaattacgttaagcatgtaataattaacatgtaatgcatgacgttatttatgagatgggtttttatgattagagtcccgcaattatacatttaatacgcgatagaaaacaaaatatagcgcgcaaactaggataaattatcgcgcgcggtgtcatctatgttactagatccgctgcttggagggaaacctcctcggattccattgcccagctatctgtcactttattgagaagatagtggaaaaggaaggtggctcctacaaatgccatcattgcgataaaggaaaggccatcgttgaagatgcctctgccgacagtggtcccaaagatggacccccacccacgaggagcatcgtggaaaaagaagacgttccaaccacgtcttcaaagcaagtggattgatgtgatatctccactgacgtaagggatgacgcacaatcccactatccttcgcaagacccttcctctatataaggaagttcatttcatttggagaggtattaaaatcttaataggttttgataaaagcgaacgtggggaaacccgaaccaaaccttcttctaaactctctctcatctctcttaaagcaaacttctctcttgtctttcttgcgtgagcgatcttcaacgttgtcagatcgtgcttcggcaccagtacaacgttttctttcactgaagcgaaatcaaagatctctttgtggacacgtagtgcggcgccattaaataacgtgtacttgtcctattcttgtcggtgtggtcttgggaaaagaaagcttgctggaggctgctgttcagccccatacattacttgttacgattctgctgactttcggcgggtgcaatatctctacttctgcttgacgaggtattgttgcctgtacttctttcttcttcttcttgctgattggttctataagaaatctagtattttctttgaaacagagttttcccgtggttttcgaacttggagaaagattgttaagcttctgtatattctgcccaaattcgcgaccggtaatggggtcatccaagaatgttatcaaggagttcatgaggtttaaggttcgcatggaaggaacggtcaatgggcacgagtttgaaatagaaggcgaaggagaggggaggccatacgaaggccacaataccgtaaagcttaaggtaaccaaggggggacctttgccatttgcttgggatattttgtcaccacaatttcagtatggaagcaaggtatatgtcaagcaccctgccgacataccagactataaaaagctgtcatttcctgaaggatttaaatgggaaagggtcatgaactttgaagatggtggcgtcgttactgtaacccaggattccagtttgcaggatggctgtttcatctacaaggtcaagttcattggcgtgaactttccttccgatggacctgttatgcaaaagaaaacaatgggctgggaagccagcactgagcgtttgtatcctcgtgatggcgtgttgaaaggagagattcataaggctctgaagctgaaagacggtggtcattacctagttgaattcaaaagtatttacatggcaaagaagcctgtgcagctaccagggtactactatgttgactccaaactggatataacaagccacaacgaagattatacaatcgttgagcagtatgaaagaaccgagggacgccaccatctgttcctttaagctttcccgggcatcaccatcaccatcactagctcgaggcctttaactctggtttcattaaattttctttagtttgaatttactgttattcggtgtgcatttctatgtttggtgagcggttttctgtgctcagagtgtgtttattttatgtaatttaatttctttgtgagctcctgtttagcaggtcgtcccttcagcaaggacacaaaaagattttaattttattaaaaaaaaaaaaaaaaaagaccgggaattcgatatcaagcttatcgacctgcagatcgttcaaacatttggcaataaagtttcttaagattgaatcctgttgccggtcttgcggtgattatcatataatttctgttgaattacgttaagcatgtaataattaacatgtaatgcatgacgttatttatgagatgggtttttatgattagagtcccgcaattatacatttaatacgcgatagaaaacaaaatatagcgcgcaaactaggataaattatcgcgcgcggtgtcatctatgttactagatccgctggtaggaggtgaattctaaacttatattgagatattagtgtataatataatttccgcactctcttttaaattaataatacaagatttagaaaaaatgaactttaattttgagatattagtgtgtaattctcagtagagaatttcctaagttcacccaaaagtatatcattttcctcttaagaaaatacaaacactacctaattttatcccctataaatatctaaaaatttgcatctcataaaatttaccaattatttattttttaagatattttactaattatctataactattaataatcaaaattatttcattgatgtacatatttcaatagataatttaccccttaatcacttaataaattttaaattttcattatttttatataatttatagtcttttttattaactatatttaaattttattttttattattaaaaaaatttagagagacacatttttccctaattagtcatatataagaaaaataacatttgggtaaaatgtgagagcccaaacgcaattcgtgttgggcctaaagggcccacgaagtagatactaaaggatgccctcatcgatgaatgcttcgctgtcaggagggaggtcaacatggtggagcacgacactctggtctactccaaaaatgtcaaagatacagtctcagaagatcaaagggctattgagacttttcaacaaaggataatttcgggaaacctcctcggattccattgcccagctatctgtcacttcatcgaaaggacagtagaaaaggaaggtggctcctacaaatgccatcattgcgataaaggaaaggctatcattcaagatctctctgccgacagtggtcccaaagatggacccccacccacgaggagcatcgtggaaaaagaagaggttccaaccacgtctacaaagcaagtggattgatgtgacatctccactgacgtaagggatgacgcacaatcccactatccttcgcaagacccttcctctatataaggaagttcatttcatttggagaggacacgctcgagtataagagctcatttttacaacaattaccaacaacaacaaacaacaaacaacattacaattacatttacaattatcgatacaatggaacgagctatacaaggaaacgacgctagggaacaagctaacagtgaacgttgggatggaggatcaggaggtaccacttctcccttcaaacttcctgacgaaagtccgagttggactgagtggcggctacataacgatgagactaattcgaatcaagataatccccttggtttcaaggaaagctggggtttcgggaaagttgtatttaagagatatctcagatacgacaggacggaagcttcactgcacagagtccttggatcttggacgggagattcggttaactatgcagcatctcgatttttcggtttcgaccagatcggatgtacctatagtattcggtttcgaggagttagtatcaccgtttctggaggctctcgaactcttcagcatctctgtgagatggcaattcggtctaagcaagaactgctacagcttgccccaatcgaagtggaaagtaatgtatcaagaggatgccctgaaggtactgaaaccttcgaaaaagaaagcgagtgagcttgtcaagcagatcgttcaaacatttggcaataaagtttcttaagattgaatcctgttgccggtcttgcgatgattatcatataatttctgttgaattacgttaagcatgtaataattaacatgtaatgcatgacgttatttatgagatgggtttttatgattagagtcccgcaattatacatttaatacgcgatagaaaacaaaatatagcgcgcaaactaggataaattatcgcgcgcggtgtcatctatgttactagatcgacgct

**pGB-E-MAR-GFP**

tgagaccgatatctgacaggatatattggcgggtaaacctaagagaaaagagcgtttattagaataatcggatatttaaaagggcgtgaaaaggtttatccgttcgtccatttgtatgtgcatgccaaccacagggttcccctcgggatcaaagtactttgatccaacccctccgctgctatagtgcagtcggcttctgacgttcagtgcagccgtcatctgaaaacgacatgtcgcacaagtcctaagttacgcgacaggctgccgccctgcccttttcctggcgttttcttgtcgcgtgttttagtcgcataaagtagaatacttgcgactagaaccggagacattacgccatgaacaagagcgccgccgctggcctgctgggctatgcccgcgtcagcaccgacgaccaggacttgaccaaccaacgggccgaactgcacgcggccggctgcaccaagctgttttccgagaagatcaccggcaccaggcgcgaccgcccggagctggccaggatgcttgaccacctacgccctggcgacgttgtgacagtgaccaggctagaccgcctggcccgcagcacccgcgacctactggacattgccgagcgcatccaggaggccggcgcgggcctgcgtagcctggcagagccgtgggccgacaccaccacgccggccggccgcatggtgttgaccgtgttcgccggcattgccgagttcgagcgttccctaatcatcgaccgcacccggagcgggcgcgaggccgccaaggcccgaggcgtgaagtttggcccccgccctaccctcaccccggcacagatcgcgcacgcccgcgagctgatcgaccaggaaggccgcaccgtgaaagaggcggctgcactgcttggcgtgcatcgctcgaccctgtaccgcgcacttgagcgcagcgaggaagtgacgcccaccgaggccaggcggcgcggtgccttccgtgaggacgcattgaccgaggccgacgccctggcggccgccgagaatgaacgccaagaggaacaagcatgaaaccgcaccaggacggccaggacgaaccgtttttcattaccgaagagatcgaggcggagatgatcgcggccgggtacgtgttcgagccgcccgcgcacctctcaaccgtgcggctgcatgaaatcctggccggtttgtctgatgccaagctggcggcctggccggccagcttggccgctgaagaaaccgagcgccgccgtctaaaaaggtgatgtgtatttgagtaaaacagcttgcgtcatgcggtcgctgcgtatatgatccgatgagtaaataaacaaatacgcaaggggaacgcatgaaggttatcgctgtacttaaccagaaaggcgggtcaggcaagacgaccatcggaacccatctagcccgcgccctgcaactcgccggggccgatgttctgttagtcgattccgatccccagggcagtgcccgcgattgggcggccgtgcgggaagatcaaccgctaaccgttgtcggcatcgaccgcccgacgattgaccgcgacgtgaaggccatcggccggcgcgacttcgtagtgatcgacggagcgccccaggcggcggacttggctgtgtccgcgatcaaggcagccgacttcgtgctgattccggtgcagccaagcccttacgacatatgggccaccgccgacctggtggagctggttaagcagcgcattgaggtcacggatggaaggctacaagcggcctttgtcgtgtcgcgggcgatcaaaggcacgcgcatcggcggtgaggttgccgaggcgctggccgggtacgagctgcccattcttgagtcccgtatcacgcagcgcgtgagctacccaggcactgccgccgccggcacaaccgttcttgaatcagaacccgagggcgacgctgcccgcgaggtccaggcgctggccgctgaaattaaatcaaaactcatttgagttaatgaggtaaagagaaaatgagcaaaagcacaaacacgctaagtgccggccgtccgagcgcacgcagcagcaaggctgcaacgttggccagcctggcagacacgccagccatgaagcgggtcaactttcagttgccggcggaggatcacaccaagctgaagatgtacgcggtacgccaaggcaagaccattaccgagctgctatctgaatagatcgcgcagctaccagagtaaatgagcaaatgaataaatgagtagatgaattttagcggctaaaggaggcggcatggaaaatcaagaacaaccaggcaccgacgccgtggaatgccccatgtgtggaggaacgggcggttggccaggcgtaagcggctgggttgtctgccggccctgcaatggcactggaacccccaagcccgaggaatcggcgtgacggtcgcaaaccatccggcccggtacaaatcggcgcggcgctgggtgatgacctggtggagaagttgaaggccgcgcaggccgcccagcggcaacgcatcgaggcagaagcacgccccggtgaatcgtggcaagcggccgctgatcgaatccgcaaagaatcccggcaaccgccggcagccggtgcgccgtcgattaggaagccgcccaagggcgacgagcaaccagattttttcgttccgatgctctatgacgtgggcacccgcgatagtcgcagcatcatggacgtggccgttttccgtctgtcgaagcgtgaccgacgagctggcgaggtgatccgctacgagcttccagacgggcacgtagaggtttccgcagggccggccggcatggccagtgtgtgggattacgacctggtactgatggcggtttcccatctaaccgaatccatgaaccgataccgggaagggaagggagacaagcccggccgcgtgttccgtccacacgttgcggacgtactcaagttctgccggcgagccgatggcggaaagcagaaagacgacctggtagaaacctgcattcggttaaacaccacgcacgttgccatgcagcgtacgaagaaggccaagaacggccgcctggtgacggtatccgagggtgaagccttgattagccgctacaagatcgtaaagagcgaaaccgggcggccggagtacatcgagatcgagctagctgattggatgtaccgcgagatcacagaaggcaagaacccggacgtgctgacggttcaccccgattactttttgatcgatcccggcatcggccgttttctctaccgcctggcacgccgcgccgcaggcaaggcagaagccagatggttgttcaagacgatctacgaacgcagtggcagcgccggagagttcaagaagttctgtttcaccgtgcgcaagctgatcgggtcaaatgacctgccggagtacgatttgaaggaggaggcggggcaggctggcccgatcctagtcatgcgctaccgcaacctgatcgagggcgaagcatccgccggttcctaatgtacggagcagatgctagggcaaattgccctagcaggggaaaaaggtcgaaaaggactctttcctgtggatagcacgtacattgggaacccaaagccgtacattgggaaccggaacccgtacattgggaacccaaagccgtacattgggaaccggtcacacatgtaagtgactgatataaaagagaaaaaaggcgatttttccgcctaaaactctttaaaacttattaaaactcttaaaacccgcctggcctgtgcataactgtctggccagcgcacagccgaagagctgcaaaaagcgcctacccttcggtcgctgcgctccctacgccccgccgcttcgcgtcggcctatcgcggccgctggccgctcaaaaatggctggcctacggccaggcaatctaccagggcgcggacaagccgcgccgtcgccactcgaccgccggcgcccacatcaaggcaccctgcctcgcgcgtttcggtgatgacggtgaaaacctctgacacatgcagctcccggtgacggtcacagcttgtctgtaagcggatgccgggagcagacaagcccgtcagggcgcgtcagcgggtgttggcgggtgtcggggcgcagccatgacccagtcacgtagcgatagcggagtgtatactggcttaactatgcggcatcagagcagattgtactgagagtgcaccatatgcggtgtgaaataccgcacagatgcgtaaggagaaaataccgcatcaggcgctcttccgcttcctcgctcactgactcgctgcgctcggtcgttcggctgcggcgagcggtatcagctcactcaaaggcggtaatacggttatccacagaatcaggggataacgcaggaaagaacatgtgagcaaaaggccagcaaaaggccaggaaccgtaaaaaggccgcgttgctggcgtttttccataggctccgcccccctgacgagcatcacaaaaatcgacgctcaagtcagaggtggcgaaacccgacaggactataaagataccaggcgtttccccctggaagctccctcgtgcgctctcctgttccgaccctgccgcttaccggatacctgtccgcctttctcccttcgggaagcgtggcgctttctcatagctcacgctgtaggtatctcagttcggtgtaggtcgttcgctccaagctgggctgtgtgcacgaaccccccgttcagcccgaccgctgcgccttatccggtaactatcgtcttgagtccaacccggtaagacacgacttatcgccactggcagcagccactggtaacaggattagcagagcgaggtatgtaggcggtgctacagagttcttgaagtggtggcctaactacggctacactagaaggacagtatttggtatctgcgctctgctgaagccagttaccttcggaaaaagagttggtagctcttgatccggcaaacaaaccaccgctggtagcggtggtttttttgtttgcaagcagcagattacgcgcagaaaaaaaggatctcaagaagatcctttgatcttttctacggggtctgacgctcagtggaacgaaaactcacgttaagggattttggtcatgcattctaggtgattatttgccgactaccttggtgatctcgcctttcacgtagtggacaaattcttccaactgatctgcgcgcgaggccaagcgatcttcttcttgtccaagataagcctgtctagcttcaagtatgacgggctgatactgggccggcaggcgctccattgcccagtcggcagcgacatccttcggcgcgattttgccggttactgcgctgtaccaaatgcgggacaacgtaagcactacatttcgctcatcaccagcccagtcgggcggcgagttccatagcgttaaggtttcatttagcgcctcaaatagatcctgttcaggaaccggatcaaagagttcctccgccgctggacctaccaaggcaacgctatgttctcttgcttttgtcagcaagatagccagatcaatgtcgatcgtggctggctcgaagatacctgcaagaatgtcattgcgctgccattctccaaattgcagttcgcgcttagctggataacgccacggaatgatgtcgtcgtgcacaacaatggtgacttctacagcgcggagaatctcgctctctccaggggaagccgaagtttccaaaaggtcgttgatcaaagctcgccgcgttgtttcatcaagccttacggtcaccgtaaccagcaaatcaatatcactgtgtggcttcaggccgccatccactgcggagccgtacaaatgtacggccagcaacgtcggttcgagatggcgctcgatgacgccaactacctctgatagttgagtcgatacttcggcgatcaccgcttccctcataatgtttaactttgttttagggcgactgccctgctgcgtaacatcgttgctgctccataacatcaaacatcgacccacggcgtaacgcgcttgctgcttggatgcccgaggcatagactgtaccccaaaaaaacagtcataacaagccatgaaaaccgccactgcgccgttaccaccgctgcgttcggtcaaggttctggaccagttgcgtgagcgcatacgctacttgcattacagcttacgaaccgaacaggcttatgtccactgggttcgtgccttcatccgtttccacggtgtgcgtcacccggcaaccttgggtagcagcgaagtcgaggcatttctgtcctggctggaacagaacttattatttccttcctcttttctacagtatttaaagataccccaagaagctaattataacaagacgaactccaattcactgttccttgcattctaaaaccttaaataccagaaaacagctttttcaaagttgttttcaaagttggcgtataacatagtatcgacggagccgattttgaaaccgcggtgatcacaggcagcaacgctctgtcatcgttacaatcaacatgctaccctccgcgagatcatccgtgtttcaaacccggcagcttagttgccgttcttccgaatagcatcggtaacatgagcaaagtctgccgccttacaacggctctcccgctgacgccgtcccggactgatgggctgcctgtatcgagtggtgattttgtgccgagctgccggtcggggagctgttggctggctggtggcaggatatattgtggtgtaaacataacgatatcggtctcagtcaggagtcgattaaaaatcccaattatatttggtctaatttagtttggtattgagtaaaacaaattcgaaccaaaccaaaatataaatatatagtttttatatatatgcctttaagactttttatagaattttctttaaaaaatatctagaaatatttgcgactcttctggcatgtaatatttcgttaaatatgaagtgctccatttttattaactttaaataattggttgtacgatcactttcttatcaagtgttactaaaatgcgtcaatctctttgttcttccatattcatatgtcaaaatctatcaaaattcttatatatctttttcgaatttgaagtgaaatttcgataatttaaaattaaatagaacatatcattatttaggtatcatattgatttttatacttaattactaaatttggttaactttgaaagtgtacatcaacgaaaaattagtcaaacgactaaaataaataaatatcatgtgttattaagaaaattctcctataagaatattttaatagatcatatgtttgtaaaaaaaattaatttttactaacacatatatttacttatcaaaaatttgacaaagtaagattaaaataatattcatctaacaaaaaaaaaaccagaaaatgctgaaaacccggcaaaaccgaaccaatccaaaccgatatagttggtttggtttgattttgatataaaccgaaccaactcggtccatttgcacccctaatcataatagctttaatatttcaagatattattaagttaacgttgtcaatatcctggaaattttgcaaaatgaatcaagcctatatggctgtaatatgaatttaaaagcagctcgatgtggtggtaatatgtaatttacttgattctaaaaaaatatcccaagtattaataatttctgctaggaagaaggttagctacgatttacagcaaagccagaatacaaagaaccataaagtgattgaagctcgaaatatacgaaggaacaaatatttttaaaaaaatacgcaatgacttggaacaaaagaaagtgatatattttttgttcttaaacaagcatcccctctaaagaatggcagttttcctttgcatgtaactattatgctcccttcgttacaaaaattttggactactattgggaacttcttctgaaaatagtcgctccatggagccctcaattgtactaccatcatttcttgttccgctgcttggagggaaacctcctcggattccattgcccagctatctgtcactttattgagaagatagtggaaaaggaaggtggctcctacaaatgccatcattgcgataaaggaaaggccatcgttgaagatgcctctgccgacagtggtcccaaagatggacccccacccacgaggagcatcgtggaaaaagaagacgttccaaccacgtcttcaaagcaagtggattgatgtgatatctccactgacgtaagggatgacgcacaatcccactatccttcgcaagacccttcctctatataaggaagttcatttcatttggagaggtattaaaatcttaataggttttgataaaagcgaacgtggggaaacccgaaccaaaccttcttctaaactctctctcatctctcttaaagcaaacttctctcttgtctttcttgcgtgagcgatcttcaacgttgtcagatcgtgcttcggcaccagtacaacgttttctttcactgaagcgaaatcaaagatctctttgtggacacgtagtgcggcgccattaaataacgtgtacttgtcctattcttgtcggtgtggtcttgggaaaagaaagcttgctggaggctgctgttcagccccatacattacttgttacgattctgctgactttcggcgggtgcaatatctctacttctgcttgacgaggtattgttgcctgtacttctttcttcttcttcttgctgattggttctataagaaatctagtattttctttgaaacagagttttcccgtggttttcgaacttggagaaagattgttaagcttctgtatattctgcccaaattcgcgaccggtaatgaatggtgagcaagggcgaggagctgttcaccggggtggtgcccatcctggtcgagctggacggcgacgtaaacggccacaagttcagcgtgtccggcgagggcgagggcgatgccacctacggcaagctgaccctgaagttcatctgcaccaccggcaagctgcccgtgccctggcccaccctcgtgaccaccttcagctacggcgtgcagtgcttcagccgctaccccgaccacatgaagcagcacgacttcttcaagtccgccatgcccgaaggctacgtccaggagcgcaccatcttcttcaaggacgacggcaactacaagacccgcgccgaggtgaagttcgagggcgacaccctggtgaaccgcatcgagctgaagggcatcgacttcaaggaggacggcaacatcctggggcacaagctggagtacaactacaacagccacaacgtctatatcatggccgacaagcagaagaacggcatcaaggtgaacttcaagatccgccacaacatcgaggacggcagcgtgcagctcgccgaccactaccagcagaacacccccatcggcgacggccccgtgctgctgcccgacaaccactacctgagcacccagtccgccctgagcaaagaccccaacgagaagcgcgatcacatggtcctgctggagttcgtgaccgccgccgggatcactcacggcatggacgagctgtacaagtaatgagctttcccgggcatcaccatcaccatcactagctcgaggcctttaactctggtttcattaaattttctttagtttgaatttactgttattcggtgtgcatttctatgtttggtgagcggttttctgtgctcagagtgtgtttattttatgtaatttaatttctttgtgagctcctgtttagcaggtcgtcccttcagcaaggacacaaaaagattttaattttattaaaaaaaaaaaaaaaaaagaccgggaattcgatatcaagcttatcgacctgcagatcgttcaaacatttggcaataaagtttcttaagattgaatcctgttgccggtcttgcggtgattatcatataatttctgttgaattacgttaagcatgtaataattaacatgtaatgcatgacgttatttatgagatgggtttttatgattagagtcccgcaattatacatttaatacgcgatagaaaacaaaatatagcgcgcaaactaggataaattatcgcgcgcggtgtcatctatgttactagatccgctggtaggaggtgaattctaaacttatattgagatattagtgtataatataatttccgcactctcttttaaattaataatacaagatttagaaaaaatgaactttaattttgagatattagtgtgtaattctcagtagagaatttcctaagttcacccaaaagtatatcattttcctcttaagaaaatacaaacactacctaattttatcccctataaatatctaaaaatttgcatctcataaaatttaccaattatttattttttaagatattttactaattatctataactattaataatcaaaattatttcattgatgtacatatttcaatagataatttaccccttaatcacttaataaattttaaattttcattatttttatataatttatagtcttttttattaactatatttaaattttattttttattattaaaaaaatttagagagacacatttttccctaattagtcatatataagaaaaataacatttgggtaaaatgtgagagcccaaacgcaattcgtgttgggcctaaagggcccacgaagtagatactaaaggatgccctcatcgatgaatgcttcgctgtcaggagggagactagagccaagctgatctcctttgccccggagatcaccatggacgactttctctatctctacgatctaggaagaaagttcgacggagaaggtgacgataccatgttcaccaccgataatgagaagattagcctcttcaatttcagaaagaatgctgacccacagatggttagagaggcctacgcggcaggtctgatcaagacgatctacccgagtaataatctccaggagatcaaataccttcccaagaaggttaaagatgcagtcaaaagattcaggactaactgcatcaagaacacagagaaagatatatttctcaagatcagaagtactattccagtatggacgattcaaggcttgcttcataaaccaaggcaagtaatagagattggagtctctaagaaagtagttcctactgaatcaaaggccatggagtcaaaaattcagatcgaggatctaacagaactcgccgtgaagactggcgaacagttcatacagagtcttttacgactcaatgacaagaagaaaatcttcgtcaacatggtggagcacgacactctcgtctactccaagaatatcaaagatacagtctcagaagaccaaagggctattgagacttttcaacaaagggtaatatcgggaaacctcctcggattccattgcccagctatctgtcacttcatcaaaaggacagtagaaaaggaaggtggcacctacaaatgccatcattgcgataaaggaaaggctatcgttcaagatgcctctgccgacagtggtcccaaagatggacccccacccacgaggagcatcgtggaaaaagaagacgttccaaccacgtcttcaaagcaagtggattgatgtgatatctccactgacgtaagggatgacgcacaatcccactatccttcgcaagacccttcctctatataaggaagttcatttcatttggagaggactccggtatttttacaacaataccacaacaaaacaaacaacaaacaacattacaatttactattctagtcgaaatggaacgagctatacaaggaaacgatgctagggaacaagcttatggtgaacgttggaatggaggatcaggaagttccacttctcccttcaaacttcctgacgaaagtccgagttggactgagtggcggctacataacgatgagactatttcgaatcaagataatccccttggtttcaaggaaagctggggtttcgggaaagttgtatttaagagatatctcagatacgacgggacggaaacttcactgcacagagtccttggatcttggacgggagattcggttaactatgcagcatctcgatttctcggtttcgaccagatcggatgtacctatagtattcggtttcgaggagttagtgtcaccatttctggagggtcgcgaactcttcagcatctcagtgaaatggcaattcggtctaagcaagaactgctacagcttaccccagtcaaagtggaaagtgatgtatcaagaggatgccctgaaggtgttgaaaccttcgaagaagaaagcgagtaagcttggaatggatcttcgatcccgatcgttcaaacatttggcaataaagtttcttaagattgaatcctgttgccggtcttgcgacgattatcatataatttctgttgaattacgttaagcatgtaataattaacatgtaatgcatgacgttatttatgagatgggtttttatgattagagtcccgcaattatacatttaatacgcgatagaaaacaaaatatagcgcgcaaactaggataaattatcgcgcdcggtgtcatctatgttactagatcgggaattgccaagctaattcttgaagacgaaagggcctcgtgatacgcctatttttataggttaatgtcatgataataatggtttcttagacgtcaggtggcacttttcggggaaatgtgcgcggaacccctatttgtttatttttctaaatacattcaaatatgtatccgctcatgagacaataaccctgataaatgcttcaataatgggaccgactcgcgct

**pGB-E-MAR-GFPR**

tgagaccgatatctgacaggatatattggcgggtaaacctaagagaaaagagcgtttattagaataatcggatatttaaaagggcgtgaaaaggtttatccgttcgtccatttgtatgtgcatgccaaccacagggttcccctcgggatcaaagtactttgatccaacccctccgctgctatagtgcagtcggcttctgacgttcagtgcagccgtcatctgaaaacgacatgtcgcacaagtcctaagttacgcgacaggctgccgccctgcccttttcctggcgttttcttgtcgcgtgttttagtcgcataaagtagaatacttgcgactagaaccggagacattacgccatgaacaagagcgccgccgctggcctgctgggctatgcccgcgtcagcaccgacgaccaggacttgaccaaccaacgggccgaactgcacgcggccggctgcaccaagctgttttccgagaagatcaccggcaccaggcgcgaccgcccggagctggccaggatgcttgaccacctacgccctggcgacgttgtgacagtgaccaggctagaccgcctggcccgcagcacccgcgacctactggacattgccgagcgcatccaggaggccggcgcgggcctgcgtagcctggcagagccgtgggccgacaccaccacgccggccggccgcatggtgttgaccgtgttcgccggcattgccgagttcgagcgttccctaatcatcgaccgcacccggagcgggcgcgaggccgccaaggcccgaggcgtgaagtttggcccccgccctaccctcaccccggcacagatcgcgcacgcccgcgagctgatcgaccaggaaggccgcaccgtgaaagaggcggctgcactgcttggcgtgcatcgctcgaccctgtaccgcgcacttgagcgcagcgaggaagtgacgcccaccgaggccaggcggcgcggtgccttccgtgaggacgcattgaccgaggccgacgccctggcggccgccgagaatgaacgccaagaggaacaagcatgaaaccgcaccaggacggccaggacgaaccgtttttcattaccgaagagatcgaggcggagatgatcgcggccgggtacgtgttcgagccgcccgcgcacctctcaaccgtgcggctgcatgaaatcctggccggtttgtctgatgccaagctggcggcctggccggccagcttggccgctgaagaaaccgagcgccgccgtctaaaaaggtgatgtgtatttgagtaaaacagcttgcgtcatgcggtcgctgcgtatatgatccgatgagtaaataaacaaatacgcaaggggaacgcatgaaggttatcgctgtacttaaccagaaaggcgggtcaggcaagacgaccatcggaacccatctagcccgcgccctgcaactcgccggggccgatgttctgttagtcgattccgatccccagggcagtgcccgcgattgggcggccgtgcgggaagatcaaccgctaaccgttgtcggcatcgaccgcccgacgattgaccgcgacgtgaaggccatcggccggcgcgacttcgtagtgatcgacggagcgccccaggcggcggacttggctgtgtccgcgatcaaggcagccgacttcgtgctgattccggtgcagccaagcccttacgacatatgggccaccgccgacctggtggagctggttaagcagcgcattgaggtcacggatggaaggctacaagcggcctttgtcgtgtcgcgggcgatcaaaggcacgcgcatcggcggtgaggttgccgaggcgctggccgggtacgagctgcccattcttgagtcccgtatcacgcagcgcgtgagctacccaggcactgccgccgccggcacaaccgttcttgaatcagaacccgagggcgacgctgcccgcgaggtccaggcgctggccgctgaaattaaatcaaaactcatttgagttaatgaggtaaagagaaaatgagcaaaagcacaaacacgctaagtgccggccgtccgagcgcacgcagcagcaaggctgcaacgttggccagcctggcagacacgccagccatgaagcgggtcaactttcagttgccggcggaggatcacaccaagctgaagatgtacgcggtacgccaaggcaagaccattaccgagctgctatctgaatagatcgcgcagctaccagagtaaatgagcaaatgaataaatgagtagatgaattttagcggctaaaggaggcggcatggaaaatcaagaacaaccaggcaccgacgccgtggaatgccccatgtgtggaggaacgggcggttggccaggcgtaagcggctgggttgtctgccggccctgcaatggcactggaacccccaagcccgaggaatcggcgtgacggtcgcaaaccatccggcccggtacaaatcggcgcggcgctgggtgatgacctggtggagaagttgaaggccgcgcaggccgcccagcggcaacgcatcgaggcagaagcacgccccggtgaatcgtggcaagcggccgctgatcgaatccgcaaagaatcccggcaaccgccggcagccggtgcgccgtcgattaggaagccgcccaagggcgacgagcaaccagattttttcgttccgatgctctatgacgtgggcacccgcgatagtcgcagcatcatggacgtggccgttttccgtctgtcgaagcgtgaccgacgagctggcgaggtgatccgctacgagcttccagacgggcacgtagaggtttccgcagggccggccggcatggccagtgtgtgggattacgacctggtactgatggcggtttcccatctaaccgaatccatgaaccgataccgggaagggaagggagacaagcccggccgcgtgttccgtccacacgttgcggacgtactcaagttctgccggcgagccgatggcggaaagcagaaagacgacctggtagaaacctgcattcggttaaacaccacgcacgttgccatgcagcgtacgaagaaggccaagaacggccgcctggtgacggtatccgagggtgaagccttgattagccgctacaagatcgtaaagagcgaaaccgggcggccggagtacatcgagatcgagctagctgattggatgtaccgcgagatcacagaaggcaagaacccggacgtgctgacggttcaccccgattactttttgatcgatcccggcatcggccgttttctctaccgcctggcacgccgcgccgcaggcaaggcagaagccagatggttgttcaagacgatctacgaacgcagtggcagcgccggagagttcaagaagttctgtttcaccgtgcgcaagctgatcgggtcaaatgacctgccggagtacgatttgaaggaggaggcggggcaggctggcccgatcctagtcatgcgctaccgcaacctgatcgagggcgaagcatccgccggttcctaatgtacggagcagatgctagggcaaattgccctagcaggggaaaaaggtcgaaaaggactctttcctgtggatagcacgtacattgggaacccaaagccgtacattgggaaccggaacccgtacattgggaacccaaagccgtacattgggaaccggtcacacatgtaagtgactgatataaaagagaaaaaaggcgatttttccgcctaaaactctttaaaacttattaaaactcttaaaacccgcctggcctgtgcataactgtctggccagcgcacagccgaagagctgcaaaaagcgcctacccttcggtcgctgcgctccctacgccccgccgcttcgcgtcggcctatcgcggccgctggccgctcaaaaatggctggcctacggccaggcaatctaccagggcgcggacaagccgcgccgtcgccactcgaccgccggcgcccacatcaaggcaccctgcctcgcgcgtttcggtgatgacggtgaaaacctctgacacatgcagctcccggtgacggtcacagcttgtctgtaagcggatgccgggagcagacaagcccgtcagggcgcgtcagcgggtgttggcgggtgtcggggcgcagccatgacccagtcacgtagcgatagcggagtgtatactggcttaactatgcggcatcagagcagattgtactgagagtgcaccatatgcggtgtgaaataccgcacagatgcgtaaggagaaaataccgcatcaggcgctcttccgcttcctcgctcactgactcgctgcgctcggtcgttcggctgcggcgagcggtatcagctcactcaaaggcggtaatacggttatccacagaatcaggggataacgcaggaaagaacatgtgagcaaaaggccagcaaaaggccaggaaccgtaaaaaggccgcgttgctggcgtttttccataggctccgcccccctgacgagcatcacaaaaatcgacgctcaagtcagaggtggcgaaacccgacaggactataaagataccaggcgtttccccctggaagctccctcgtgcgctctcctgttccgaccctgccgcttaccggatacctgtccgcctttctcccttcgggaagcgtggcgctttctcatagctcacgctgtaggtatctcagttcggtgtaggtcgttcgctccaagctgggctgtgtgcacgaaccccccgttcagcccgaccgctgcgccttatccggtaactatcgtcttgagtccaacccggtaagacacgacttatcgccactggcagcagccactggtaacaggattagcagagcgaggtatgtaggcggtgctacagagttcttgaagtggtggcctaactacggctacactagaaggacagtatttggtatctgcgctctgctgaagccagttaccttcggaaaaagagttggtagctcttgatccggcaaacaaaccaccgctggtagcggtggtttttttgtttgcaagcagcagattacgcgcagaaaaaaaggatctcaagaagatcctttgatcttttctacggggtctgacgctcagtggaacgaaaactcacgttaagggattttggtcatgcattctaggtgattatttgccgactaccttggtgatctcgcctttcacgtagtggacaaattcttccaactgatctgcgcgcgaggccaagcgatcttcttcttgtccaagataagcctgtctagcttcaagtatgacgggctgatactgggccggcaggcgctccattgcccagtcggcagcgacatccttcggcgcgattttgccggttactgcgctgtaccaaatgcgggacaacgtaagcactacatttcgctcatcaccagcccagtcgggcggcgagttccatagcgttaaggtttcatttagcgcctcaaatagatcctgttcaggaaccggatcaaagagttcctccgccgctggacctaccaaggcaacgctatgttctcttgcttttgtcagcaagatagccagatcaatgtcgatcgtggctggctcgaagatacctgcaagaatgtcattgcgctgccattctccaaattgcagttcgcgcttagctggataacgccacggaatgatgtcgtcgtgcacaacaatggtgacttctacagcgcggagaatctcgctctctccaggggaagccgaagtttccaaaaggtcgttgatcaaagctcgccgcgttgtttcatcaagccttacggtcaccgtaaccagcaaatcaatatcactgtgtggcttcaggccgccatccactgcggagccgtacaaatgtacggccagcaacgtcggttcgagatggcgctcgatgacgccaactacctctgatagttgagtcgatacttcggcgatcaccgcttccctcataatgtttaactttgttttagggcgactgccctgctgcgtaacatcgttgctgctccataacatcaaacatcgacccacggcgtaacgcgcttgctgcttggatgcccgaggcatagactgtaccccaaaaaaacagtcataacaagccatgaaaaccgccactgcgccgttaccaccgctgcgttcggtcaaggttctggaccagttgcgtgagcgcatacgctacttgcattacagcttacgaaccgaacaggcttatgtccactgggttcgtgccttcatccgtttccacggtgtgcgtcacccggcaaccttgggtagcagcgaagtcgaggcatttctgtcctggctggaacagaacttattatttccttcctcttttctacagtatttaaagataccccaagaagctaattataacaagacgaactccaattcactgttccttgcattctaaaaccttaaataccagaaaacagctttttcaaagttgttttcaaagttggcgtataacatagtatcgacggagccgattttgaaaccgcggtgatcacaggcagcaacgctctgtcatcgttacaatcaacatgctaccctccgcgagatcatccgtgtttcaaacccggcagcttagttgccgttcttccgaatagcatcggtaacatgagcaaagtctgccgccttacaacggctctcccgctgacgccgtcccggactgatgggctgcctgtatcgagtggtgattttgtgccgagctgccggtcggggagctgttggctggctggtggcaggatatattgtggtgtaaacataacgatatcggtctcagtcaggagtcgattaaaaatcccaattatatttggtctaatttagtttggtattgagtaaaacaaattcgaaccaaaccaaaatataaatatatagtttttatatatatgcctttaagactttttatagaattttctttaaaaaatatctagaaatatttgcgactcttctggcatgtaatatttcgttaaatatgaagtgctccatttttattaactttaaataattggttgtacgatcactttcttatcaagtgttactaaaatgcgtcaatctctttgttcttccatattcatatgtcaaaatctatcaaaattcttatatatctttttcgaatttgaagtgaaatttcgataatttaaaattaaatagaacatatcattatttaggtatcatattgatttttatacttaattactaaatttggttaactttgaaagtgtacatcaacgaaaaattagtcaaacgactaaaataaataaatatcatgtgttattaagaaaattctcctataagaatattttaatagatcatatgtttgtaaaaaaaattaatttttactaacacatatatttacttatcaaaaatttgacaaagtaagattaaaataatattcatctaacaaaaaaaaaaccagaaaatgctgaaaacccggcaaaaccgaaccaatccaaaccgatatagttggtttggtttgattttgatataaaccgaaccaactcggtccatttgcacccctaatcataatagctttaatatttcaagatattattaagttaacgttgtcaatatcctggaaattttgcaaaatgaatcaagcctatatggctgtaatatgaatttaaaagcagctcgatgtggtggtaatatgtaatttacttgattctaaaaaaatatcccaagtattaataatttctgctaggaagaaggttagctacgatttacagcaaagccagaatacaaagaaccataaagtgattgaagctcgaaatatacgaaggaacaaatatttttaaaaaaatacgcaatgacttggaacaaaagaaagtgatatattttttgttcttaaacaagcatcccctctaaagaatggcagttttcctttgcatgtaactattatgctcccttcgttacaaaaattttggactactattgggaacttcttctgaaaatagtcgctccatggagccctcaattgtactaccatcatttcttgttccgctgcttagcggatctagtaacatagatgacaccgcgcgcgataatttatcctagtttgcgcgctatattttgttttctatcgcgtattaaatgtataattgcgggactctaatcataaaaacccatctcataaataacgtcatgcattacatgttaattattacatgcttaacgtaattcaacagaaattatatgataatcgtcgcaagaccggcaacaggattcaatcttaagaaactttattgccaaatgtttgaacgatctgcaggtcgataagcttgatatcgaattcccggtcttttttttttttttttttaataaaattaaaatctttttgtgtccttgctgaagggacgacctgctaaacaggagctcacaaagaaattaaattacataaaataaacacactctgagcacagaaaaccgctcaccaaacatagaaatgcacaccgaataacagtaaattcaaactaaagaaaatttaatgaaaccagagttaaaggcctcgagctagtgatggtgatggtgatgcccgggaTGATGGTGATGGTGATGCCCGGGaagcttacttgtacagctcgtccatgccgtgagtgatcccggcggcggtcacgaactccagcaggaccatgtgatcgcgcttctcgttggggtctttgctcagggcggactgggtgctcaggtagtggttgtcgggcagcagcacggggccgtcgccgatgggggtgttctgctggtagtggtcggcgagctgcacgctgccgtcctcgatgttgtggcggatcttgaagttcaccttgatgccgttcttctgcttgtcggccatgatatagacgttgtggctgttgtagttgtactccagcttgtgccccaggatgttgccgtcctccttgaagtcgatgcccttcagctcgatgcggttcaccagggtgtcgccctcgaacttcacctcggcgcgggtcttgtagttgccgtcgtccttgaagaagatggtgcgctcctggacgtagccttcgggcatggcggacttgaagaagtcgtgctgcttcatgtggtcggggtagcggctgaagcactgcacgccgtagctgaaggtggtcacgagggtgggccagggcacgggcagcttgccggtggtgcagatgaacttcagggtcagcttgccgtaggtggcatcgccctcgccctcgccggacacgctgaacttgtggccgtttacgtcgccgtccagctcgaccaggatgggcaccaccccggtgaacagctcctcgcccttgctcaccattaccggtcgcgaatttgggcagaatatacagaagcttaacaatctttctccaagttcgaaaaccacgggaaaactctgtttcaaagaaaatactagatttcttatagaaccaatcagcaagaagaagaagaaagaagtacaggcaacaatacctcgtcaagcagaagtagagatattgcacccgccgaaagtcagcagaatcgtaacaagtaatgtatggggctgaacagcagcctccagcaagctttcttttcccaagaccacaccgacaagaataggacaagtacacgttatttaatggcgccgcactacgtgtccacaaagagatctttgatttcgcttcagtgaaagaaaacgttgtactggtgccgaagcacgatctgacaacgttgaagatcgctcacgcaagaaagacaagagagaagtttgctttaagagagatgagagagagtttagaagaaggtttggttcgggtttccccacgttcgcttttatcaaaacctattaagattttaatacctctccaaatgaaatgaacttccttatatagaggaagggtcttgcgaaggatagtgggattgtgcgtcatcccttacgtcagtggagatatcacatcaatccacttgctttgaagacgtggttggaacgtcttctttttccacgatgctcctcgtgggtgggggtccatctttgggaccactgtcggcagaggcatcttcaacgatggcctttcctttatcgcaatgatggcatttgtaggagccaccttccttttccactatcttctcaataaagtgacagatagctgggcaatggaatccgaggaggtttccCTCCGGTAggaggtgaattctaaacttatattgagatattagtgtataatataatttccgcactctcttttaaattaataatacaagatttagaaaaaatgaactttaattttgagatattagtgtgtaattctcagtagagaatttcctaagttcacccaaaagtatatcattttcctcttaagaaaatacaaacactacctaattttatcccctataaatatctaaaaatttgcatctcataaaatttaccaattatttattttttaagatattttactaattatctataactattaataatcaaaattatttcattgatgtacatatttcaatagataatttaccccttaatcacttaataaattttaaattttcattatttttatataatttatagtcttttttattaactatatttaaattttattttttattattaaaaaaatttagagagacacatttttccctaattagtcatatataagaaaaataacatttgggtaaaatgtgagagcccaaacgcaattcgtgttgggcctaaagggcccacgaagtagatactaaaggatgccctcatcgatgaatgcttcgctgtcaggagggagactagagccaagctgatctcctttgccccggagatcaccatggacgactttctctatctctacgatctaggaagaaagttcgacggagaaggtgacgataccatgttcaccaccgataatgagaagattagcctcttcaatttcagaaagaatgctgacccacagatggttagagaggcctacgcggcaggtctgatcaagacgatctacccgagtaataatctccaggagatcaaataccttcccaagaaggttaaagatgcagtcaaaagattcaggactaactgcatcaagaacacagagaaagatatatttctcaagatcagaagtactattccagtatggacgattcaaggcttgcttcataaaccaaggcaagtaatagagattggagtctctaagaaagtagttcctactgaatcaaaggccatggagtcaaaaattcagatcgaggatctaacagaactcgccgtgaagactggcgaacagttcatacagagtcttttacgactcaatgacaagaagaaaatcttcgtcaacatggtggagcacgacactctcgtctactccaagaatatcaaagatacagtctcagaagaccaaagggctattgagacttttcaacaaagggtaatatcgggaaacctcctcggattccattgcccagctatctgtcacttcatcaaaaggacagtagaaaaggaaggtggcacctacaaatgccatcattgcgataaaggaaaggctatcgttcaagatgcctctgccgacagtggtcccaaagatggacccccacccacgaggagcatcgtggaaaaagaagacgttccaaccacgtcttcaaagcaagtggattgatgtgatatctccactgacgtaagggatgacgcacaatcccactatccttcgcaagacccttcctctatataaggaagttcatttcatttggagaggactccggtatttttacaacaataccacaacaaaacaaacaacaaacaacattacaatttactattctagtcgaaatggaacgagctatacaaggaaacgatgctagggaacaagcttatggtgaacgttggaatggaggatcaggaagttccacttctcccttcaaacttcctgacgaaagtccgagttggactgagtggcggctacataacgatgagactatttcgaatcaagataatccccttggtttcaaggaaagctggggtttcgggaaagttgtatttaagagatatctcagatacgacgggacggaaacttcactgcacagagtccttggatcttggacgggagattcggttaactatgcagcatctcgatttctcggtttcgaccagatcggatgtacctatagtattcggtttcgaggagttagtgtcaccatttctggagggtcgcgaactcttcagcatctcagtgaaatggcaattcggtctaagcaagaactgctacagcttaccccagtcaaagtggaaagtgatgtatcaagaggatgccctgaaggtgttgaaaccttcgaagaagaaagcgagtaagcttggaatggatcttcgatcccgatcgttcaaacatttggcaataaagtttcttaagattgaatcctgttgccggtcttgcgacgattatcatataatttctgttgaattacgttaagcatgtaataattaacatgtaatgcatgacgttatttatgagatgggtttttatgattagagtcccgcaattatacatttaatacgcgatagaaaacaaaatatagcgcgcaaactaggataaattatcgcgcdcggtgtcatctatgttactagatcgggaattgccaagctaattcttgaagacgaaagggcctcgtgatacgcctatttttataggttaatgtcatgataataatggtttcttagacgtcaggtggcacttttcggggaaatgtgcgcggaacccctatttgtttatttttctaaatacattcaaatatgtatccgctcatgagacaataaccctgataaatgcttcaataatgggaccgactcgcgct

**pGB-E-MAR-GFPi**

tgagaccgatatctgacaggatatattggcgggtaaacctaagagaaaagagcgtttattagaataatcggatatttaaaagggcgtgaaaaggtttatccgttcgtccatttgtatgtgcatgccaaccacagggttcccctcgggatcaaagtactttgatccaacccctccgctgctatagtgcagtcggcttctgacgttcagtgcagccgtcatctgaaaacgacatgtcgcacaagtcctaagttacgcgacaggctgccgccctgcccttttcctggcgttttcttgtcgcgtgttttagtcgcataaagtagaatacttgcgactagaaccggagacattacgccatgaacaagagcgccgccgctggcctgctgggctatgcccgcgtcagcaccgacgaccaggacttgaccaaccaacgggccgaactgcacgcggccggctgcaccaagctgttttccgagaagatcaccggcaccaggcgcgaccgcccggagctggccaggatgcttgaccacctacgccctggcgacgttgtgacagtgaccaggctagaccgcctggcccgcagcacccgcgacctactggacattgccgagcgcatccaggaggccggcgcgggcctgcgtagcctggcagagccgtgggccgacaccaccacgccggccggccgcatggtgttgaccgtgttcgccggcattgccgagttcgagcgttccctaatcatcgaccgcacccggagcgggcgcgaggccgccaaggcccgaggcgtgaagtttggcccccgccctaccctcaccccggcacagatcgcgcacgcccgcgagctgatcgaccaggaaggccgcaccgtgaaagaggcggctgcactgcttggcgtgcatcgctcgaccctgtaccgcgcacttgagcgcagcgaggaagtgacgcccaccgaggccaggcggcgcggtgccttccgtgaggacgcattgaccgaggccgacgccctggcggccgccgagaatgaacgccaagaggaacaagcatgaaaccgcaccaggacggccaggacgaaccgtttttcattaccgaagagatcgaggcggagatgatcgcggccgggtacgtgttcgagccgcccgcgcacctctcaaccgtgcggctgcatgaaatcctggccggtttgtctgatgccaagctggcggcctggccggccagcttggccgctgaagaaaccgagcgccgccgtctaaaaaggtgatgtgtatttgagtaaaacagcttgcgtcatgcggtcgctgcgtatatgatccgatgagtaaataaacaaatacgcaaggggaacgcatgaaggttatcgctgtacttaaccagaaaggcgggtcaggcaagacgaccatcggaacccatctagcccgcgccctgcaactcgccggggccgatgttctgttagtcgattccgatccccagggcagtgcccgcgattgggcggccgtgcgggaagatcaaccgctaaccgttgtcggcatcgaccgcccgacgattgaccgcgacgtgaaggccatcggccggcgcgacttcgtagtgatcgacggagcgccccaggcggcggacttggctgtgtccgcgatcaaggcagccgacttcgtgctgattccggtgcagccaagcccttacgacatatgggccaccgccgacctggtggagctggttaagcagcgcattgaggtcacggatggaaggctacaagcggcctttgtcgtgtcgcgggcgatcaaaggcacgcgcatcggcggtgaggttgccgaggcgctggccgggtacgagctgcccattcttgagtcccgtatcacgcagcgcgtgagctacccaggcactgccgccgccggcacaaccgttcttgaatcagaacccgagggcgacgctgcccgcgaggtccaggcgctggccgctgaaattaaatcaaaactcatttgagttaatgaggtaaagagaaaatgagcaaaagcacaaacacgctaagtgccggccgtccgagcgcacgcagcagcaaggctgcaacgttggccagcctggcagacacgccagccatgaagcgggtcaactttcagttgccggcggaggatcacaccaagctgaagatgtacgcggtacgccaaggcaagaccattaccgagctgctatctgaatagatcgcgcagctaccagagtaaatgagcaaatgaataaatgagtagatgaattttagcggctaaaggaggcggcatggaaaatcaagaacaaccaggcaccgacgccgtggaatgccccatgtgtggaggaacgggcggttggccaggcgtaagcggctgggttgtctgccggccctgcaatggcactggaacccccaagcccgaggaatcggcgtgacggtcgcaaaccatccggcccggtacaaatcggcgcggcgctgggtgatgacctggtggagaagttgaaggccgcgcaggccgcccagcggcaacgcatcgaggcagaagcacgccccggtgaatcgtggcaagcggccgctgatcgaatccgcaaagaatcccggcaaccgccggcagccggtgcgccgtcgattaggaagccgcccaagggcgacgagcaaccagattttttcgttccgatgctctatgacgtgggcacccgcgatagtcgcagcatcatggacgtggccgttttccgtctgtcgaagcgtgaccgacgagctggcgaggtgatccgctacgagcttccagacgggcacgtagaggtttccgcagggccggccggcatggccagtgtgtgggattacgacctggtactgatggcggtttcccatctaaccgaatccatgaaccgataccgggaagggaagggagacaagcccggccgcgtgttccgtccacacgttgcggacgtactcaagttctgccggcgagccgatggcggaaagcagaaagacgacctggtagaaacctgcattcggttaaacaccacgcacgttgccatgcagcgtacgaagaaggccaagaacggccgcctggtgacggtatccgagggtgaagccttgattagccgctacaagatcgtaaagagcgaaaccgggcggccggagtacatcgagatcgagctagctgattggatgtaccgcgagatcacagaaggcaagaacccggacgtgctgacggttcaccccgattactttttgatcgatcccggcatcggccgttttctctaccgcctggcacgccgcgccgcaggcaaggcagaagccagatggttgttcaagacgatctacgaacgcagtggcagcgccggagagttcaagaagttctgtttcaccgtgcgcaagctgatcgggtcaaatgacctgccggagtacgatttgaaggaggaggcggggcaggctggcccgatcctagtcatgcgctaccgcaacctgatcgagggcgaagcatccgccggttcctaatgtacggagcagatgctagggcaaattgccctagcaggggaaaaaggtcgaaaaggactctttcctgtggatagcacgtacattgggaacccaaagccgtacattgggaaccggaacccgtacattgggaacccaaagccgtacattgggaaccggtcacacatgtaagtgactgatataaaagagaaaaaaggcgatttttccgcctaaaactctttaaaacttattaaaactcttaaaacccgcctggcctgtgcataactgtctggccagcgcacagccgaagagctgcaaaaagcgcctacccttcggtcgctgcgctccctacgccccgccgcttcgcgtcggcctatcgcggccgctggccgctcaaaaatggctggcctacggccaggcaatctaccagggcgcggacaagccgcgccgtcgccactcgaccgccggcgcccacatcaaggcaccctgcctcgcgcgtttcggtgatgacggtgaaaacctctgacacatgcagctcccggtgacggtcacagcttgtctgtaagcggatgccgggagcagacaagcccgtcagggcgcgtcagcgggtgttggcgggtgtcggggcgcagccatgacccagtcacgtagcgatagcggagtgtatactggcttaactatgcggcatcagagcagattgtactgagagtgcaccatatgcggtgtgaaataccgcacagatgcgtaaggagaaaataccgcatcaggcgctcttccgcttcctcgctcactgactcgctgcgctcggtcgttcggctgcggcgagcggtatcagctcactcaaaggcggtaatacggttatccacagaatcaggggataacgcaggaaagaacatgtgagcaaaaggccagcaaaaggccaggaaccgtaaaaaggccgcgttgctggcgtttttccataggctccgcccccctgacgagcatcacaaaaatcgacgctcaagtcagaggtggcgaaacccgacaggactataaagataccaggcgtttccccctggaagctccctcgtgcgctctcctgttccgaccctgccgcttaccggatacctgtccgcctttctcccttcgggaagcgtggcgctttctcatagctcacgctgtaggtatctcagttcggtgtaggtcgttcgctccaagctgggctgtgtgcacgaaccccccgttcagcccgaccgctgcgccttatccggtaactatcgtcttgagtccaacccggtaagacacgacttatcgccactggcagcagccactggtaacaggattagcagagcgaggtatgtaggcggtgctacagagttcttgaagtggtggcctaactacggctacactagaaggacagtatttggtatctgcgctctgctgaagccagttaccttcggaaaaagagttggtagctcttgatccggcaaacaaaccaccgctggtagcggtggtttttttgtttgcaagcagcagattacgcgcagaaaaaaaggatctcaagaagatcctttgatcttttctacggggtctgacgctcagtggaacgaaaactcacgttaagggattttggtcatgcattctaggtgattatttgccgactaccttggtgatctcgcctttcacgtagtggacaaattcttccaactgatctgcgcgcgaggccaagcgatcttcttcttgtccaagataagcctgtctagcttcaagtatgacgggctgatactgggccggcaggcgctccattgcccagtcggcagcgacatccttcggcgcgattttgccggttactgcgctgtaccaaatgcgggacaacgtaagcactacatttcgctcatcaccagcccagtcgggcggcgagttccatagcgttaaggtttcatttagcgcctcaaatagatcctgttcaggaaccggatcaaagagttcctccgccgctggacctaccaaggcaacgctatgttctcttgcttttgtcagcaagatagccagatcaatgtcgatcgtggctggctcgaagatacctgcaagaatgtcattgcgctgccattctccaaattgcagttcgcgcttagctggataacgccacggaatgatgtcgtcgtgcacaacaatggtgacttctacagcgcggagaatctcgctctctccaggggaagccgaagtttccaaaaggtcgttgatcaaagctcgccgcgttgtttcatcaagccttacggtcaccgtaaccagcaaatcaatatcactgtgtggcttcaggccgccatccactgcggagccgtacaaatgtacggccagcaacgtcggttcgagatggcgctcgatgacgccaactacctctgatagttgagtcgatacttcggcgatcaccgcttccctcataatgtttaactttgttttagggcgactgccctgctgcgtaacatcgttgctgctccataacatcaaacatcgacccacggcgtaacgcgcttgctgcttggatgcccgaggcatagactgtaccccaaaaaaacagtcataacaagccatgaaaaccgccactgcgccgttaccaccgctgcgttcggtcaaggttctggaccagttgcgtgagcgcatacgctacttgcattacagcttacgaaccgaacaggcttatgtccactgggttcgtgccttcatccgtttccacggtgtgcgtcacccggcaaccttgggtagcagcgaagtcgaggcatttctgtcctggctggaacagaacttattatttccttcctcttttctacagtatttaaagataccccaagaagctaattataacaagacgaactccaattcactgttccttgcattctaaaaccttaaataccagaaaacagctttttcaaagttgttttcaaagttggcgtataacatagtatcgacggagccgattttgaaaccgcggtgatcacaggcagcaacgctctgtcatcgttacaatcaacatgctaccctccgcgagatcatccgtgtttcaaacccggcagcttagttgccgttcttccgaatagcatcggtaacatgagcaaagtctgccgccttacaacggctctcccgctgacgccgtcccggactgatgggctgcctgtatcgagtggtgattttgtgccgagctgccggtcggggagctgttggctggctggtggcaggatatattgtggtgtaaacataacgatatcggtctcagtcaggagtcgattaaaaatcccaattatatttggtctaatttagtttggtattgagtaaaacaaattcgaaccaaaccaaaatataaatatatagtttttatatatatgcctttaagactttttatagaattttctttaaaaaatatctagaaatatttgcgactcttctggcatgtaatatttcgttaaatatgaagtgctccatttttattaactttaaataattggttgtacgatcactttcttatcaagtgttactaaaatgcgtcaatctctttgttcttccatattcatatgtcaaaatctatcaaaattcttatatatctttttcgaatttgaagtgaaatttcgataatttaaaattaaatagaacatatcattatttaggtatcatattgatttttatacttaattactaaatttggttaactttgaaagtgtacatcaacgaaaaattagtcaaacgactaaaataaataaatatcatgtgttattaagaaaattctcctataagaatattttaatagatcatatgtttgtaaaaaaaattaatttttactaacacatatatttacttatcaaaaatttgacaaagtaagattaaaataatattcatctaacaaaaaaaaaaccagaaaatgctgaaaacccggcaaaaccgaaccaatccaaaccgatatagttggtttggtttgattttgatataaaccgaaccaactcggtccatttgcacccctaatcataatagctttaatatttcaagatattattaagttaacgttgtcaatatcctggaaattttgcaaaatgaatcaagcctatatggctgtaatatgaatttaaaagcagctcgatgtggtggtaatatgtaatttacttgattctaaaaaaatatcccaagtattaataatttctgctaggaagaaggttagctacgatttacagcaaagccagaatacaaagaaccataaagtgattgaagctcgaaatatacgaaggaacaaatatttttaaaaaaatacgcaatgacttggaacaaaagaaagtgatatattttttgttcttaaacaagcatcccctctaaagaatggcagttttcctttgcatgtaactattatgctcccttcgttacaaaaattttggactactattgggaacttcttctgaaaatagtcgctccatggagccctcaattgtactaccatcatttcttgttccgctgcttggagggaaacctcctcggattccattgcccagctatctgtcactttattgagaagatagtggaaaaggaaggtggctcctacaaatgccatcattgcgataaaggaaaggccatcgttgaagatgcctctgccgacagtggtcccaaagatggacccccacccacgaggagcatcgtggaaaaagaagacgttccaaccacgtcttcaaagcaagtggattgatgtgatatctccactgacgtaagggatgacgcacaatcccactatccttcgcaagacccttcctctatataaggaagttcatttcatttggagaggtattaaaatcttaataggttttgataaaagcgaacgtggggaaacccgaaccaaaccttcttctaaactctctctcatctctcttaaagcaaacttctctcttgtctttcttgcgtgagcgatcttcaacgttgtcagatcgtgcttcggcaccagtacaacgttttctttcactgaagcgaaatcaaagatctctttgtggacacgtagtgcggcgccattaaataacgtgtacttgtcctattcttgtcggtgtggtcttgggaaaagaaagcttgctggaggctgctgttcagccccatacattacttgttacgattctgctgactttcggcgggtgcaatatctctacttctgcttgacgaggtattgttgcctgtacttctttcttcttcttcttgctgattggttctataagaaatctagtattttctttgaaacagagttttcccgtggttttcgaacttggagaaagattgttaagcttctgtatattctgcccaaattcgcgaccggtccatAGGTACTTCATGCTTCAACGTGTAACTTAAGAGATACTGTGTGAAATTTTATATTTCCATACATTTGCTTGACCTTTGCTTTTTGTCAATTTTTTTCCCCTTACAGGTAAAAatggtgagcaagggcgaggagctgttcaccggggtggtgcccatcctggtcgagctggacggcgacgtaaacggccacaagttcagcgtgtccggcgagggcgagggcgatgccacctacggcaagctgaccctgaagttcatctgcaccaccggcaagctgcccgtgccctggcccaccctcgtgaccaccttcagctacggcgtgcagtgcttcagccgctaccccgaccacatgaagcagcacgacttcttcaagtccgccatgcccgaaggctacgtccaggagcgcaccatcttcttcaaggacgacggcaactacaagacccgcgccgaggtgaagttcgagggcgacaccctggtgaaccgcatcgagctgaagggcatcgacttcaaggaggacggcaacatcctggggcacaagctggagtacaactacaacagccacaacgtctatatcatggccgacaagcagaagaacggcatcaaggtgaacttcaagatccgccacaacatcgaggacggcagcgtgcagctcgccgaccactaccagcagaacacccccatcggcgacggccccgtgctgctgcccgacaaccactacctgagcacccagtccgccctgagcaaagaccccaacgagaagcgcgatcacatggtcctgctggagttcgtgaccgccgccgggatcactcacggcatggacgagctgtacaagtaactttcccgggcatcaccatcaccatcactagctcgaggcctttaactctggtttcattaaattttctttagtttgaatttactgttattcggtgtgcatttctatgtttggtgagcggttttctgtgctcagagtgtgtttattttatgtaatttaatttctttgtgagctcctgtttagcaggtcgtcccttcagcaaggacacaaaaagattttaattttattaaaaaaaaaaaaaaaaaagaccgggaattcgatatcaagcttatcgacctgcagatcgttcaaacatttggcaataaagtttcttaagattgaatcctgttgccggtcttgcggtgattatcatataatttctgttgaattacgttaagcatgtaataattaacatgtaatgcatgacgttatttatgagatgggtttttatgattagagtcccgcaattatacatttaatacgcgatagaaaacaaaatatagcgcgcaaactaggataaattatcgcgcgcggtgtcatctatgttactagatccgctggtaggaggtgaattctaaacttatattgagatattagtgtataatataatttccgcactctcttttaaattaataatacaagatttagaaaaaatgaactttaattttgagatattagtgtgtaattctcagtagagaatttcctaagttcacccaaaagtatatcattttcctcttaagaaaatacaaacactacctaattttatcccctataaatatctaaaaatttgcatctcataaaatttaccaattatttattttttaagatattttactaattatctataactattaataatcaaaattatttcattgatgtacatatttcaatagataatttaccccttaatcacttaataaattttaaattttcattatttttatataatttatagtcttttttattaactatatttaaattttattttttattattaaaaaaatttagagagacacatttttccctaattagtcatatataagaaaaataacatttgggtaaaatgtgagagcccaaacgcaattcgtgttgggcctaaagggcccacgaagtagatactaaaggatgccctcatcgatgaatgcttcgctgtcaggagggagactagagccaagctgatctcctttgccccggagatcaccatggacgactttctctatctctacgatctaggaagaaagttcgacggagaaggtgacgataccatgttcaccaccgataatgagaagattagcctcttcaatttcagaaagaatgctgacccacagatggttagagaggcctacgcggcaggtctgatcaagacgatctacccgagtaataatctccaggagatcaaataccttcccaagaaggttaaagatgcagtcaaaagattcaggactaactgcatcaagaacacagagaaagatatatttctcaagatcagaagtactattccagtatggacgattcaaggcttgcttcataaaccaaggcaagtaatagagattggagtctctaagaaagtagttcctactgaatcaaaggccatggagtcaaaaattcagatcgaggatctaacagaactcgccgtgaagactggcgaacagttcatacagagtcttttacgactcaatgacaagaagaaaatcttcgtcaacatggtggagcacgacactctcgtctactccaagaatatcaaagatacagtctcagaagaccaaagggctattgagacttttcaacaaagggtaatatcgggaaacctcctcggattccattgcccagctatctgtcacttcatcaaaaggacagtagaaaaggaaggtggcacctacaaatgccatcattgcgataaaggaaaggctatcgttcaagatgcctctgccgacagtggtcccaaagatggacccccacccacgaggagcatcgtggaaaaagaagacgttccaaccacgtcttcaaagcaagtggattgatgtgatatctccactgacgtaagggatgacgcacaatcccactatccttcgcaagacccttcctctatataaggaagttcatttcatttggagaggactccggtatttttacaacaataccacaacaaaacaaacaacaaacaacattacaatttactattctagtcgaaatggaacgagctatacaaggaaacgatgctagggaacaagcttatggtgaacgttggaatggaggatcaggaagttccacttctcccttcaaacttcctgacgaaagtccgagttggactgagtggcggctacataacgatgagactatttcgaatcaagataatccccttggtttcaaggaaagctggggtttcgggaaagttgtatttaagagatatctcagatacgacgggacggaaacttcactgcacagagtccttggatcttggacgggagattcggttaactatgcagcatctcgatttctcggtttcgaccagatcggatgtacctatagtattcggtttcgaggagttagtgtcaccatttctggagggtcgcgaactcttcagcatctcagtgaaatggcaattcggtctaagcaagaactgctacagcttaccccagtcaaagtggaaagtgatgtatcaagaggatgccctgaaggtgttgaaaccttcgaagaagaaagcgagtaagcttggaatggatcttcgatcccgatcgttcaaacatttggcaataaagtttcttaagattgaatcctgttgccggtcttgcgacgattatcatataatttctgttgaattacgttaagcatgtaataattaacatgtaatgcatgacgttatttatgagatgggtttttatgattagagtcccgcaattatacatttaatacgcgatagaaaacaaaatatagcgcgcaaactaggataaattatcgcgcdcggtgtcatctatgttactagatcgggaattgccaagctaattcttgaagacgaaagggcctcgtgatacgcctatttttataggttaatgtcatgataataatggtttcttagacgtcaggtggcacttttcggggaaatgtgcgcggaacccctatttgtttatttttctaaatacattcaaatatgtatccgctcatgagacaataaccctgataaatgcttcaataatgggaccgactcgcgct

**pGB-E-GFP**

tgagaccgatatctgacaggatatattggcgggtaaacctaagagaaaagagcgtttattagaataatcggatatttaaaagggcgtgaaaaggtttatccgttcgtccatttgtatgtgcatgccaaccacagggttcccctcgggatcaaagtactttgatccaacccctccgctgctatagtgcagtcggcttctgacgttcagtgcagccgtcatctgaaaacgacatgtcgcacaagtcctaagttacgcgacaggctgccgccctgcccttttcctggcgttttcttgtcgcgtgttttagtcgcataaagtagaatacttgcgactagaaccggagacattacgccatgaacaagagcgccgccgctggcctgctgggctatgcccgcgtcagcaccgacgaccaggacttgaccaaccaacgggccgaactgcacgcggccggctgcaccaagctgttttccgagaagatcaccggcaccaggcgcgaccgcccggagctggccaggatgcttgaccacctacgccctggcgacgttgtgacagtgaccaggctagaccgcctggcccgcagcacccgcgacctactggacattgccgagcgcatccaggaggccggcgcgggcctgcgtagcctggcagagccgtgggccgacaccaccacgccggccggccgcatggtgttgaccgtgttcgccggcattgccgagttcgagcgttccctaatcatcgaccgcacccggagcgggcgcgaggccgccaaggcccgaggcgtgaagtttggcccccgccctaccctcaccccggcacagatcgcgcacgcccgcgagctgatcgaccaggaaggccgcaccgtgaaagaggcggctgcactgcttggcgtgcatcgctcgaccctgtaccgcgcacttgagcgcagcgaggaagtgacgcccaccgaggccaggcggcgcggtgccttccgtgaggacgcattgaccgaggccgacgccctggcggccgccgagaatgaacgccaagaggaacaagcatgaaaccgcaccaggacggccaggacgaaccgtttttcattaccgaagagatcgaggcggagatgatcgcggccgggtacgtgttcgagccgcccgcgcacctctcaaccgtgcggctgcatgaaatcctggccggtttgtctgatgccaagctggcggcctggccggccagcttggccgctgaagaaaccgagcgccgccgtctaaaaaggtgatgtgtatttgagtaaaacagcttgcgtcatgcggtcgctgcgtatatgatccgatgagtaaataaacaaatacgcaaggggaacgcatgaaggttatcgctgtacttaaccagaaaggcgggtcaggcaagacgaccatcggaacccatctagcccgcgccctgcaactcgccggggccgatgttctgttagtcgattccgatccccagggcagtgcccgcgattgggcggccgtgcgggaagatcaaccgctaaccgttgtcggcatcgaccgcccgacgattgaccgcgacgtgaaggccatcggccggcgcgacttcgtagtgatcgacggagcgccccaggcggcggacttggctgtgtccgcgatcaaggcagccgacttcgtgctgattccggtgcagccaagcccttacgacatatgggccaccgccgacctggtggagctggttaagcagcgcattgaggtcacggatggaaggctacaagcggcctttgtcgtgtcgcgggcgatcaaaggcacgcgcatcggcggtgaggttgccgaggcgctggccgggtacgagctgcccattcttgagtcccgtatcacgcagcgcgtgagctacccaggcactgccgccgccggcacaaccgttcttgaatcagaacccgagggcgacgctgcccgcgaggtccaggcgctggccgctgaaattaaatcaaaactcatttgagttaatgaggtaaagagaaaatgagcaaaagcacaaacacgctaagtgccggccgtccgagcgcacgcagcagcaaggctgcaacgttggccagcctggcagacacgccagccatgaagcgggtcaactttcagttgccggcggaggatcacaccaagctgaagatgtacgcggtacgccaaggcaagaccattaccgagctgctatctgaatagatcgcgcagctaccagagtaaatgagcaaatgaataaatgagtagatgaattttagcggctaaaggaggcggcatggaaaatcaagaacaaccaggcaccgacgccgtggaatgccccatgtgtggaggaacgggcggttggccaggcgtaagcggctgggttgtctgccggccctgcaatggcactggaacccccaagcccgaggaatcggcgtgacggtcgcaaaccatccggcccggtacaaatcggcgcggcgctgggtgatgacctggtggagaagttgaaggccgcgcaggccgcccagcggcaacgcatcgaggcagaagcacgccccggtgaatcgtggcaagcggccgctgatcgaatccgcaaagaatcccggcaaccgccggcagccggtgcgccgtcgattaggaagccgcccaagggcgacgagcaaccagattttttcgttccgatgctctatgacgtgggcacccgcgatagtcgcagcatcatggacgtggccgttttccgtctgtcgaagcgtgaccgacgagctggcgaggtgatccgctacgagcttccagacgggcacgtagaggtttccgcagggccggccggcatggccagtgtgtgggattacgacctggtactgatggcggtttcccatctaaccgaatccatgaaccgataccgggaagggaagggagacaagcccggccgcgtgttccgtccacacgttgcggacgtactcaagttctgccggcgagccgatggcggaaagcagaaagacgacctggtagaaacctgcattcggttaaacaccacgcacgttgccatgcagcgtacgaagaaggccaagaacggccgcctggtgacggtatccgagggtgaagccttgattagccgctacaagatcgtaaagagcgaaaccgggcggccggagtacatcgagatcgagctagctgattggatgtaccgcgagatcacagaaggcaagaacccggacgtgctgacggttcaccccgattactttttgatcgatcccggcatcggccgttttctctaccgcctggcacgccgcgccgcaggcaaggcagaagccagatggttgttcaagacgatctacgaacgcagtggcagcgccggagagttcaagaagttctgtttcaccgtgcgcaagctgatcgggtcaaatgacctgccggagtacgatttgaaggaggaggcggggcaggctggcccgatcctagtcatgcgctaccgcaacctgatcgagggcgaagcatccgccggttcctaatgtacggagcagatgctagggcaaattgccctagcaggggaaaaaggtcgaaaaggactctttcctgtggatagcacgtacattgggaacccaaagccgtacattgggaaccggaacccgtacattgggaacccaaagccgtacattgggaaccggtcacacatgtaagtgactgatataaaagagaaaaaaggcgatttttccgcctaaaactctttaaaacttattaaaactcttaaaacccgcctggcctgtgcataactgtctggccagcgcacagccgaagagctgcaaaaagcgcctacccttcggtcgctgcgctccctacgccccgccgcttcgcgtcggcctatcgcggccgctggccgctcaaaaatggctggcctacggccaggcaatctaccagggcgcggacaagccgcgccgtcgccactcgaccgccggcgcccacatcaaggcaccctgcctcgcgcgtttcggtgatgacggtgaaaacctctgacacatgcagctcccggtgacggtcacagcttgtctgtaagcggatgccgggagcagacaagcccgtcagggcgcgtcagcgggtgttggcgggtgtcggggcgcagccatgacccagtcacgtagcgatagcggagtgtatactggcttaactatgcggcatcagagcagattgtactgagagtgcaccatatgcggtgtgaaataccgcacagatgcgtaaggagaaaataccgcatcaggcgctcttccgcttcctcgctcactgactcgctgcgctcggtcgttcggctgcggcgagcggtatcagctcactcaaaggcggtaatacggttatccacagaatcaggggataacgcaggaaagaacatgtgagcaaaaggccagcaaaaggccaggaaccgtaaaaaggccgcgttgctggcgtttttccataggctccgcccccctgacgagcatcacaaaaatcgacgctcaagtcagaggtggcgaaacccgacaggactataaagataccaggcgtttccccctggaagctccctcgtgcgctctcctgttccgaccctgccgcttaccggatacctgtccgcctttctcccttcgggaagcgtggcgctttctcatagctcacgctgtaggtatctcagttcggtgtaggtcgttcgctccaagctgggctgtgtgcacgaaccccccgttcagcccgaccgctgcgccttatccggtaactatcgtcttgagtccaacccggtaagacacgacttatcgccactggcagcagccactggtaacaggattagcagagcgaggtatgtaggcggtgctacagagttcttgaagtggtggcctaactacggctacactagaaggacagtatttggtatctgcgctctgctgaagccagttaccttcggaaaaagagttggtagctcttgatccggcaaacaaaccaccgctggtagcggtggtttttttgtttgcaagcagcagattacgcgcagaaaaaaaggatctcaagaagatcctttgatcttttctacggggtctgacgctcagtggaacgaaaactcacgttaagggattttggtcatgcattctaggtgattatttgccgactaccttggtgatctcgcctttcacgtagtggacaaattcttccaactgatctgcgcgcgaggccaagcgatcttcttcttgtccaagataagcctgtctagcttcaagtatgacgggctgatactgggccggcaggcgctccattgcccagtcggcagcgacatccttcggcgcgattttgccggttactgcgctgtaccaaatgcgggacaacgtaagcactacatttcgctcatcaccagcccagtcgggcggcgagttccatagcgttaaggtttcatttagcgcctcaaatagatcctgttcaggaaccggatcaaagagttcctccgccgctggacctaccaaggcaacgctatgttctcttgcttttgtcagcaagatagccagatcaatgtcgatcgtggctggctcgaagatacctgcaagaatgtcattgcgctgccattctccaaattgcagttcgcgcttagctggataacgccacggaatgatgtcgtcgtgcacaacaatggtgacttctacagcgcggagaatctcgctctctccaggggaagccgaagtttccaaaaggtcgttgatcaaagctcgccgcgttgtttcatcaagccttacggtcaccgtaaccagcaaatcaatatcactgtgtggcttcaggccgccatccactgcggagccgtacaaatgtacggccagcaacgtcggttcgagatggcgctcgatgacgccaactacctctgatagttgagtcgatacttcggcgatcaccgcttccctcataatgtttaactttgttttagggcgactgccctgctgcgtaacatcgttgctgctccataacatcaaacatcgacccacggcgtaacgcgcttgctgcttggatgcccgaggcatagactgtaccccaaaaaaacagtcataacaagccatgaaaaccgccactgcgccgttaccaccgctgcgttcggtcaaggttctggaccagttgcgtgagcgcatacgctacttgcattacagcttacgaaccgaacaggcttatgtccactgggttcgtgccttcatccgtttccacggtgtgcgtcacccggcaaccttgggtagcagcgaagtcgaggcatttctgtcctggctggaacagaacttattatttccttcctcttttctacagtatttaaagataccccaagaagctaattataacaagacgaactccaattcactgttccttgcattctaaaaccttaaataccagaaaacagctttttcaaagttgttttcaaagttggcgtataacatagtatcgacggagccgattttgaaaccgcggtgatcacaggcagcaacgctctgtcatcgttacaatcaacatgctaccctccgcgagatcatccgtgtttcaaacccggcagcttagttgccgttcttccgaatagcatcggtaacatgagcaaagtctgccgccttacaacggctctcccgctgacgccgtcccggactgatgggctgcctgtatcgagtggtgattttgtgccgagctgccggtcggggagctgttggctggctggtggcaggatatattgtggtgtaaacataacgatatcggtctcagtcaggagggaaacctcctcggattccattgcccagctatctgtcactttattgagaagatagtggaaaaggaaggtggctcctacaaatgccatcattgcgataaaggaaaggccatcgttgaagatgcctctgccgacagtggtcccaaagatggacccccacccacgaggagcatcgtggaaaaagaagacgttccaaccacgtcttcaaagcaagtggattgatgtgatatctccactgacgtaagggatgacgcacaatcccactatccttcgcaagacccttcctctatataaggaagttcatttcatttggagaggtattaaaatcttaataggttttgataaaagcgaacgtggggaaacccgaaccaaaccttcttctaaactctctctcatctctcttaaagcaaacttctctcttgtctttcttgcgtgagcgatcttcaacgttgtcagatcgtgcttcggcaccagtacaacgttttctttcactgaagcgaaatcaaagatctctttgtggacacgtagtgcggcgccattaaataacgtgtacttgtcctattcttgtcggtgtggtcttgggaaaagaaagcttgctggaggctgctgttcagccccatacattacttgttacgattctgctgactttcggcgggtgcaatatctctacttctgcttgacgaggtattgttgcctgtacttctttcttcttcttcttgctgattggttctataagaaatctagtattttctttgaaacagagttttcccgtggttttcgaacttggagaaagattgttaagcttctgtatattctgcccaaattcgcgaccggtaatgaatggtgagcaagggcgaggagctgttcaccggggtggtgcccatcctggtcgagctggacggcgacgtaaacggccacaagttcagcgtgtccggcgagggcgagggcgatgccacctacggcaagctgaccctgaagttcatctgcaccaccggcaagctgcccgtgccctggcccaccctcgtgaccaccttcagctacggcgtgcagtgcttcagccgctaccccgaccacatgaagcagcacgacttcttcaagtccgccatgcccgaaggctacgtccaggagcgcaccatcttcttcaaggacgacggcaactacaagacccgcgccgaggtgaagttcgagggcgacaccctggtgaaccgcatcgagctgaagggcatcgacttcaaggaggacggcaacatcctggggcacaagctggagtacaactacaacagccacaacgtctatatcatggccgacaagcagaagaacggcatcaaggtgaacttcaagatccgccacaacatcgaggacggcagcgtgcagctcgccgaccactaccagcagaacacccccatcggcgacggccccgtgctgctgcccgacaaccactacctgagcacccagtccgccctgagcaaagaccccaacgagaagcgcgatcacatggtcctgctggagttcgtgaccgccgccgggatcactcacggcatggacgagctgtacaagtaatgagctttcccgggcatcaccatcaccatcactagctcgaggcctttaactctggtttcattaaattttctttagtttgaatttactgttattcggtgtgcatttctatgtttggtgagcggttttctgtgctcagagtgtgtttattttatgtaatttaatttctttgtgagctcctgtttagcaggtcgtcccttcagcaaggacacaaaaagattttaattttattaaaaaaaaaaaaaaaaaagaccgggaattcgatatcaagcttatcgacctgcagatcgttcaaacatttggcaataaagtttcttaagattgaatcctgttgccggtcttgcggtgattatcatataatttctgttgaattacgttaagcatgtaataattaacatgtaatgcatgacgttatttatgagatgggtttttatgattagagtcccgcaattatacatttaatacgcgatagaaaacaaaatatagcgcgcaaactaggataaattatcgcgcgcggtgtcatctatgttactagatccgctgtcaggagggagactagagccaagctgatctcctttgccccggagatcaccatggacgactttctctatctctacgatctaggaagaaagttcgacggagaaggtgacgataccatgttcaccaccgataatgagaagattagcctcttcaatttcagaaagaatgctgacccacagatggttagagaggcctacgcggcaggtctgatcaagacgatctacccgagtaataatctccaggagatcaaataccttcccaagaaggttaaagatgcagtcaaaagattcaggactaactgcatcaagaacacagagaaagatatatttctcaagatcagaagtactattccagtatggacgattcaaggcttgcttcataaaccaaggcaagtaatagagattggagtctctaagaaagtagttcctactgaatcaaaggccatggagtcaaaaattcagatcgaggatctaacagaactcgccgtgaagactggcgaacagttcatacagagtcttttacgactcaatgacaagaagaaaatcttcgtcaacatggtggagcacgacactctcgtctactccaagaatatcaaagatacagtctcagaagaccaaagggctattgagacttttcaacaaagggtaatatcgggaaacctcctcggattccattgcccagctatctgtcacttcatcaaaaggacagtagaaaaggaaggtggcacctacaaatgccatcattgcgataaaggaaaggctatcgttcaagatgcctctgccgacagtggtcccaaagatggacccccacccacgaggagcatcgtggaaaaagaagacgttccaaccacgtcttcaaagcaagtggattgatgtgatatctccactgacgtaagggatgacgcacaatcccactatccttcgcaagacccttcctctatataaggaagttcatttcatttggagaggactccggtatttttacaacaataccacaacaaaacaaacaacaaacaacattacaatttactattctagtcgaaatggaacgagctatacaaggaaacgatgctagggaacaagcttatggtgaacgttggaatggaggatcaggaagttccacttctcccttcaaacttcctgacgaaagtccgagttggactgagtggcggctacataacgatgagactatttcgaatcaagataatccccttggtttcaaggaaagctggggtttcgggaaagttgtatttaagagatatctcagatacgacgggacggaaacttcactgcacagagtccttggatcttggacgggagattcggttaactatgcagcatctcgatttctcggtttcgaccagatcggatgtacctatagtattcggtttcgaggagttagtgtcaccatttctggagggtcgcgaactcttcagcatctcagtgaaatggcaattcggtctaagcaagaactgctacagcttaccccagtcaaagtggaaagtgatgtatcaagaggatgccctgaaggtgttgaaaccttcgaagaagaaagcgagtaagcttggaatggatcttcgatcccgatcgttcaaacatttggcaataaagtttcttaagattgaatcctgttgccggtcttgcgacgattatcatataatttctgttgaattacgttaagcatgtaataattaacatgtaatgcatgacgttatttatgagatgggtttttatgattagagtcccgcaattatacatttaatacgcgatagaaaacaaaatatagcgcgcaaactaggataaattatcgcgcdcggtgtcatctatgttactagatcgggaattgccaagctaattcttgaagacgaaagggcctcgtgatacgcctatttttataggttaatgtcatgataataatggtttcttagacgtcaggtggcacttttcggggaaatgtgcgcggaacccctatttgtttatttttctaaatacattcaaatatgtatccgctcatgagacaataaccctgataaatgcttcaataatgggaccgactcgcgct

## Supplementary Figures

**Supplementary Figure 1:** Composite microscopic image showing fluorescence of nascent (GFP channel) and mature (DsRed channel) fluorescent Timer reporter protein. The images were taken 2 weeks after leaf infiltration with the non-replicating construct. The maturation halftime of reporter protein is reported as 3-5 hours.

**Supplementary Figure 2:** **Necrosis on leaves**. Leaves were infiltrated by non-replicating (upper row) and replicating (lower row) *A. tumefaciens* EHA 105 harbouring respective plasmid constructs. Photographs were taken at 12 DPI.
