# Supplementary figures and images for: Extended Set of GoldenBraid Compatible Vectors for Fast Assembly of Multigenic Constructs and Their Use to Create Geminiviral Expression Vectors
Source: Front Plant Sci. 2020 Oct 22;11:522059. doi: 10.3389/fpls.2020.522059 (PMC7641900; doi:10.3389/fpls.2020.522059)

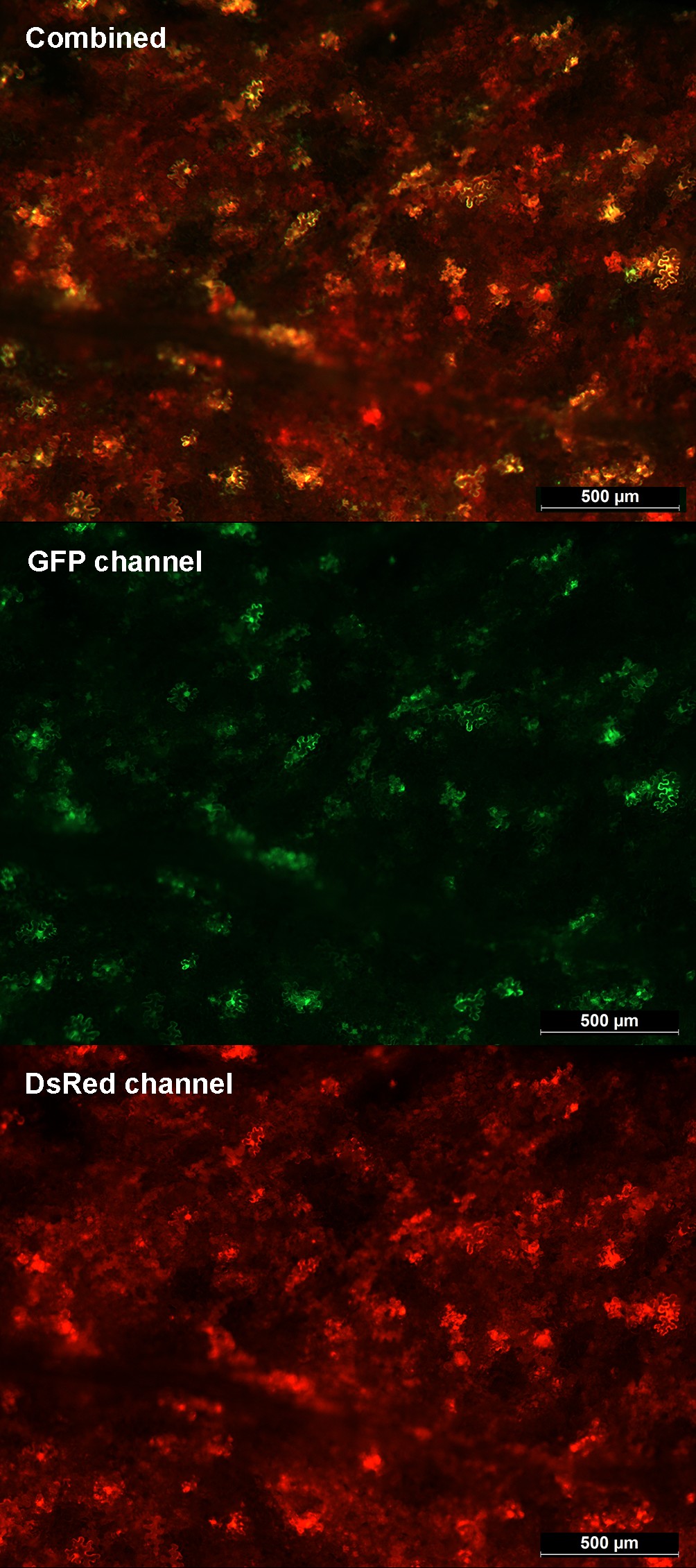

Supplement: Supplementary file 2 [file Image_1.TIF]

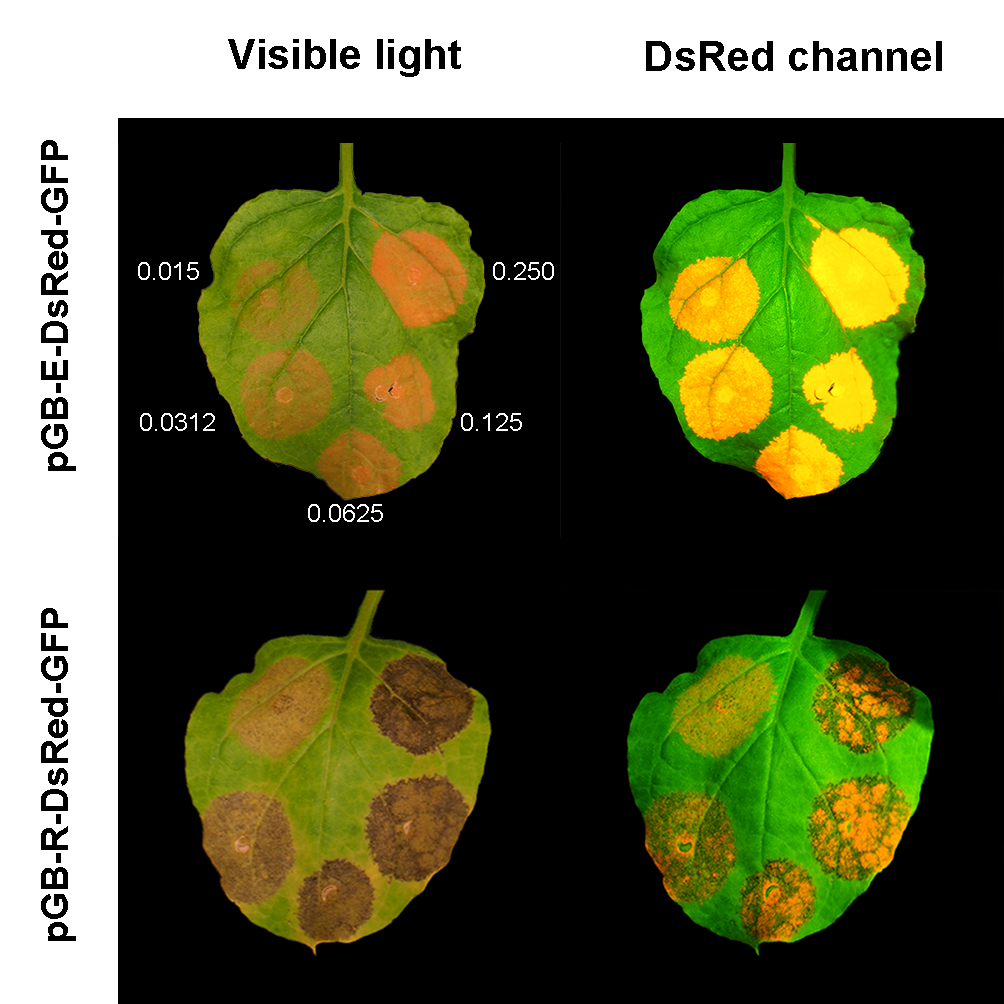

Supplement: Supplementary file 3 [file Image_2.TIF]
